# Near-unity near-infrared emission from Mo_6_ cluster doped hybrid halide glasses via halide-ligand and matrix engineering

**DOI:** 10.1038/s41467-026-75934-5

**Published:** 2026-07-22

**Authors:** Xuhui Feng, Peiqing Cai, Da Lei, Keyuan Liu, Haohan Wang, Hang Yin, Qinhua Wei, Zutao Fan, Zhaoyang Chen, Shala Bi, Zugang Liu

**Affiliations:** 1 College of Optical and Electronic Technology, China Jiliang University, Hangzhou, China; 2 Key Laboratory of Green and High-end Utilization of Salt Lake Resources, Qinghai Institute of Salt Lakes, Chinese Academy of Sciences, Xining, China; 3 Qinghai Provincial Key Laboratory of Resources and Chemistry of Salt Lakes, Qinghai Institute of Salt Lakes, Chinese Academy of Sciences, Xining, China; 4 College of Materials and Chemistry, China Jiliang University, Hangzhou, China; 5 International Collaborative Laboratory of 2D Materials for Optoelectronics Science Technology of Ministry of Education, Institute of Microscale Optoelectronics, Shenzhen University, Shenzhen, China; 6 State Key Laboratory of Functional Materials and Devices for Special Environments Conditions, Xinjiang Technical Institute of Physics and Chemistry of CAS, Urumqi, China; 7 Xinjiang Key Laboratory of Electronic Information Materials and Devices, Xinjiang Technical Institute of Physics and Chemistry of CAS, Urumqi, China

**Keywords:** Inorganic LEDs, Optical materials, Materials for optics

## Abstract

Efficient and air-stable near-infrared emitters are needed for compact optical systems, but translating highly emissive molecular triplet emitters into processable bulk solids remains challenging because aggregation, molecular motion and oxygen deactivate long-lived excited states. Here we show that a molecular hybrid halide glass can serve as a protective and regulating host for the triplet emission of molybdenum cluster. By comparing isostructural cluster salts with chloride, bromide and iodide terminal ligands, we establish how apical halide chemistry controls electronic structure and near-infrared radiative dynamics. The chloride-terminated cluster shows the highest efficiency among the molecular salts. When incorporated into a cadmium chloride-based molecular hybrid glass, the clusters become molecularly dispersed, while matrix-dictated halide redistribution and amorphous confinement suppress aggregation, vibrational deactivation and oxygen accessibility. The optimized glass achieves 96.95% internal quantum efficiency and 91.43% external quantum efficiency in air, enabling compact near-infrared illumination for imaging applications.

## Abstract (web summary)

This study shows that molecular hybrid metal halide glass can protect molybdenum cluster emitters from oxygen quenching, enabling efficient air-stable near-infrared light sources for imaging.

## Introduction

Efficient and air-stable near-infrared (NIR) emitters are essential for compact optical systems used in biomedical imaging, nondestructive inspection, night-vision technologies, optical sensing and photonic communication^1–3^. Compared with visible emitters, NIR-emissive materials are more susceptible to nonradiative energy loss because the reduced emission energy generally enhances multi-phonon relaxation^4^. Therefore, practical NIR light-conversion materials must simultaneously combine high intrinsic photoluminescence efficiency, strong absorption, environmental stability and compatibility with processable solid-state device configurations^5,6^.

Several classes of NIR-emissive materials have been developed to address these requirements. Rare-earth-doped phosphors exhibit excellent photostability and characteristic NIR emission, but their parity-forbidden *f*-*f* transitions usually lead to weak absorption and limited excitation efficiency^7^. Cr^3+^-activated inorganic phosphors have recently emerged as highly efficient broadband NIR emitters through crystal-field and host-lattice engineering, with several systems reaching near-unity internal quantum efficiencies^8–10^. However, the spin- and parity-forbidden nature of Cr^3+^ *d*-*d* transitions still limits absorption strength and can constrain external quantum efficiency, while high-temperature solid-state synthesis may restrict compositional flexibility and low-temperature processing^11,12^. Semiconductor quantum dots and metal nanoclusters provide stronger absorption and higher structural tunability, but their solid-state implementation is frequently limited by aggregation, environmental sensitivity, surface or ligand instability, and device-integration challenges^13–15^. These trade-offs highlight the need for a solid-state NIR-emitter platform that combines strong absorption, high luminescence efficiency, air stability and processability.

Octahedral Mo_6_ halide clusters are representative molecular NIR triplet emitters that meet several of these criteria. Their optical absorption mainly originates from charge-transfer and metal-metal-related transitions, giving rise to relatively strong absorption compared with many parity-forbidden ion-based emitters^16,17^. In addition, the emission energy, lifetime and quantum efficiency of Mo_6_ clusters can be tuned through inner- and apical-ligand engineering. It has been well established that {Mo_6_I_8_}^4+^-based clusters, especially under deoxygenated conditions, can display high photoluminescence quantum yields^18^, and their oxygen-sensitive phosphorescence, temperature-dependent emission and excited-state structures have been systematically investigated^19–21^. However, high molecular efficiency in deoxygenated solution does not directly translate into air-stable and processable solid-state emission. In solution, the long-lived triplet excited states are highly accessible to dissolved oxygen and undergo efficient dynamic quenching. In powders or crystalline salts, cluster–cluster interactions, surface exposure and limited processability can induce aggregation-related luminescent quenching^22^. Polymer matrices can provide physical dilution and film-forming ability, but their chain mobility and free volume may still allow oxygen permeation, and they generally do not regulate the local halide environment of the embedded clusters^23^. Therefore, an effective host for Mo_6_-based solid-state NIR emission should simultaneously isolate the molecular clusters, rigidify their local environment and reduce oxygen accessibility.

Molecular hybrid halide glasses have recently emerged as a class of amorphous ionic materials that integrate the compositional tunability of molecular halides with the transparency, shape processability and structural rigidity of glassy solids^24–28^. Unlike conventional polymer hosts, these glasses are constructed from ionically assembled organic cations and discrete metal-halide units, enabling the local halide environment, lattice softness and excited-state confinement to be adjusted through molecular composition. Recent studies have demonstrated their potential in broadband emission, room-temperature phosphorescence, scintillation and radiation-detection applications^26,29–33^. These features make molecular hybrid halide glasses particularly attractive as ionically interactive and structurally confining hosts for oxygen-sensitive molecular triplet emitters. During melt processing, a halide-rich ionic melt can promote molecular-level dispersion of charged Mo_6_ cluster anions and enable matrix-mediated halide redistribution around the cluster. Upon vitrification, the resulting rigid amorphous matrix can freeze the dispersed emissive centers, suppress cluster aggregation, and reduce the effective accessibility of oxygen to the long-lived triplet excited state^34^.

Here we demonstrate this concept through coupled halide-ligand and glass-matrix engineering of Mo_6_ cluster emitters. A series of isostructural (ETP)_2_[{Mo_6_I_8_}X_6_] (X = Cl, Br and I; ETP = ethyltriphenylphosphonium) molecular salts are synthesized to clarify how apical halide chemistry regulates Mo-X bonding, frontier-orbital distribution, zero-field splitting (ZFS) and NIR triplet emission dynamics. The Cl-terminated cluster shows the highest efficiency among the molecular salts because of stronger Mo-Cl bonding and a more rigid cluster core. This optimized cluster is then embedded into an (ETP)_2_CdCl_4_ molecular hybrid halide glass, where the halide-rich ionic melt enables cluster dispersion and matrix-dictated halide redistribution, while the rigid vitrified matrix suppresses aggregation, vibrational deactivation and oxygen-mediated triplet quenching. The resulting glass achieves near-unity NIR emission efficiency in air and is further integrated into a compact NIR illumination prototype. This work establishes molecular hybrid halide glass as a solid-state platform for protecting and regulating Mo_6_-cluster triplet emission.

## Results and discussion

**Fig. 1:**
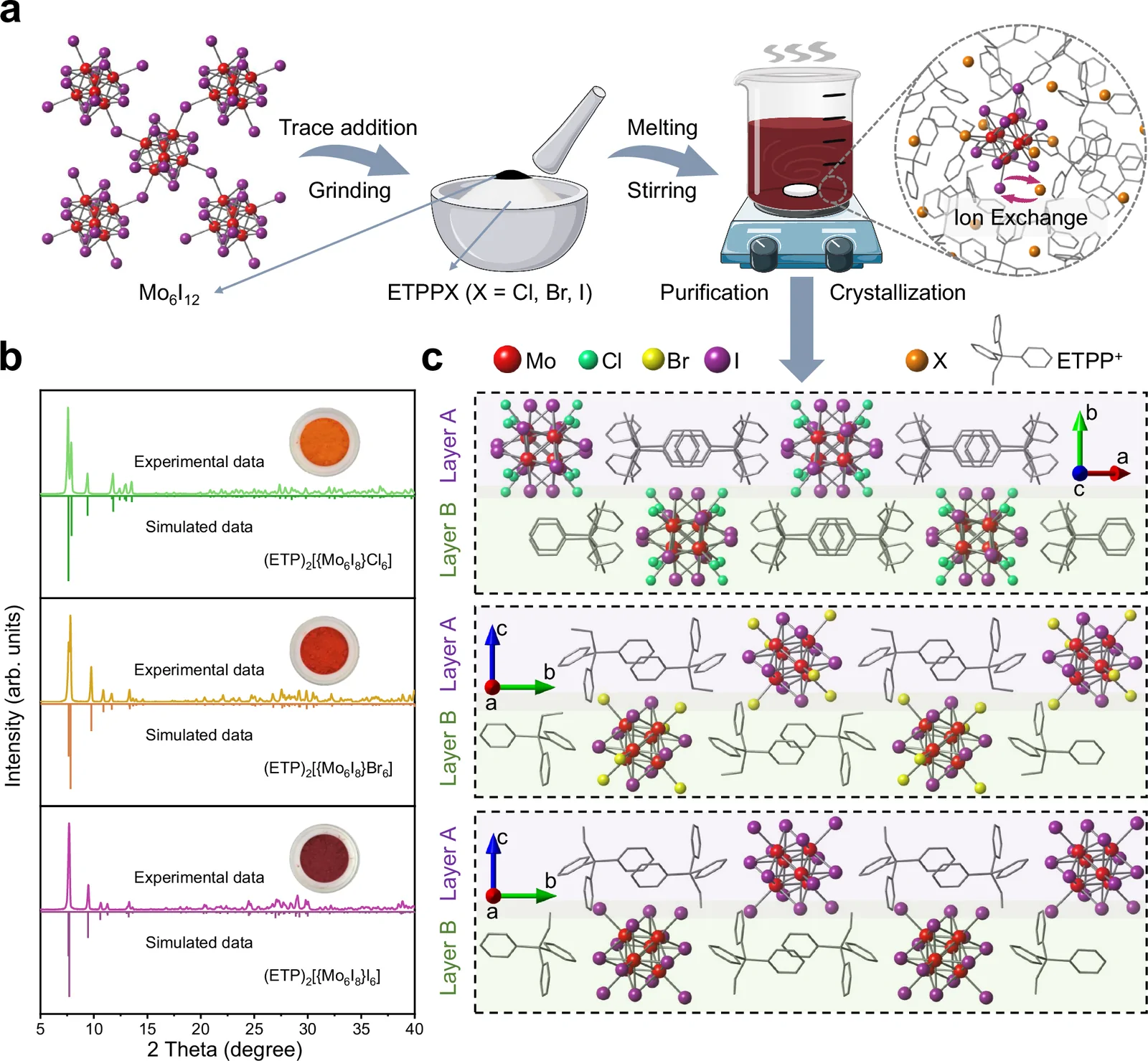
Synthesis and crystal structure of molybdenum cluster compounds. **a** Schematic illustration of the synthetic pathway for (ETP)_2_[{Mo_6_I_8_}X_6_]. **b** Experimental and simulated powder X-ray diffraction (XRD) patterns of the obtained cluster compounds. **c** Layered crystal structure arrangement of (ETP)_2_[{Mo_6_I_8_}X_6_]. Source data are provided as a Source Data file.

Three molybdenum cluster samples were prepared via high-temperature ionic liquid ligand-exchange methodology. Initially, molten ionic liquids ETPPX (X = Cl, Br, I) served as solvents to react with the molybdenum precursor Mo_6_I_12_, yielding (ETP)_2_[{Mo_6_I_8_}X_6_] (ETPP or ETP: ethyl-triphenylphosphonium). As shown in Fig. 1a, the reaction proceeds through the evolution of a layered structure, where two-dimensional Mo_6_I_12_ clusters consist of octahedral {Mo_6_I_8_}^4+^ cores linked via apical iodides (I). Subsequently, Mo_6_I_12_ was mixed with an excess of ETPPX and heated to 240–290 °C, melting ETPPX into an ionic liquid and providing a high-temperature polar ionic environment for complete dissolution of Mo_6_I_12_. Under these conditions, I-I bridging bonds between adjacent {Mo_6_I_8_}^4+^ units are cleaved, and the terminal iodide ligands are replaced by high-concentration X^−^ ions (Cl^−^ or Br^−^) from the environment. The high-temperature polar ionic liquid further facilitates this substitution. After reaction, methanol washing removed excess ETPPX, yielding the methanol-insoluble (ETP)_2_[{Mo_6_I_8_}X_6_] precipitate. Recrystallization from N, N-dimethylformamide (DMF) solution of the (ETP)_2_[{Mo_6_I_8_}X_6_] precipitate under diethyl ether vapor in a glovebox afforded single crystals. The resulting ionic crystals feature electrostatic interactions between ETPP^+^ cations and [{Mo_6_I_8_}X_6_]^2−^ anions. This synthetic route parallels the formation of {[Mo_6_Cl_8_]Cl_6_}^2−^ from Mo_6_Cl_12_ in neutral AlCl_3_-MeEtimCl ionic liquids^35^. Similarly, in ETPPI high-temperature liquid, Mo_6_I_12_ exists as {[Mo_6_I_8_]I_6_}^2−^, and using ETPPCl or ETPPBr as reaction solution leads to apical ligand substitution, generating {[Mo_6_I_8_]Cl_6_}^2−^ or {[Mo_6_I_8_]Br_6_}^2−^ clusters. Notably, the highly stable {Mo_6_I_8_}^4+^ core resists apical ligand substitution under typical conditions^16^.

Single-crystal X-ray diffraction (XRD) analysis revealed that (ETP)_2_[{Mo_6_I_8_}Cl_6_] crystallizes in a monoclinic system with space group C2/c and unit cell parameters of *a* = 27.8690 Å, *b* = 13.5829 Å, *c* = 18.8710 Å, and *β* = 126.701; (ETP)_2_[{Mo_6_I_8_}Br_6_] also adopts a monoclinic system with space group P2_1_/n and unit cell parameters *a* = 9.8610 Å, *b* = 23.1140 Å, *c* = 12.9754 Å, and *β* = 94.058°. For comparison, the previously reported I-terminated analogue, (ETP)_2_[{Mo_6_I_8_}I_6_], similarly belongs to the monoclinic system, space group P2_1_/n, with *a* = 10.2309 Å, *b* = 23.2103 Å, *c* = 13.2272 Å, and *β* = 93.711°. This structure has been deposited at the Cambridge Crystallographic Data Centre under CCDC 2425267 and reported in our previous work^34^. The Cl- and Br-terminated structures newly determined in this study have been deposited under CCDC 2477364 and 2477363, respectively. Detailed crystallographic data for all three structures are summarized in Supplementary Table 1. Additionally, dropping the DMF solutions of the molybdenum clusters into excess diethyl ether afforded the corresponding polycrystalline powders. Powder XRD patterns matched well with the simulated single-crystal patterns, indicating the products are phase-pure (Fig. 1b). The bulk color of the polycrystalline powders changes from yellow to red to deep red as the apical ligands increase in atomic weight, consistent with the diffuse-reflectance-derived absorption spectra (Supplementary Fig. 1).

The obtained (ETP)_2_[{Mo_6_I_8_}Cl_6_], (ETP)_2_[{Mo_6_I_8_}Br_6_], and (ETP)_2_[{Mo_6_I_8_}I_6_] all exhibit a layered ionic crystal structure. As shown in Fig. 1c, the crystals consist of anionic clusters [{Mo_6_I_8_}X_6_]^2−^ and cationic ETPP^+^ ions, which stack orderly via electrostatic interactions. Taking (ETP)_2_[{Mo_6_I_8_}Cl_6_] as an example, viewed along the *z*-axis, the molybdenum clusters and organic cations alternate to form a layered structure, with adjacent layers staggered to construct a three-dimensional crystal framework (Supplementary Fig. 2). The structure exhibits periodic stacking along the *y*-axis, with relatively weak interlayer interactions but overall structural stability. (ETP)_2_[{Mo_6_I_8_}Br_6_] and (ETP)_2_[{Mo_6_I_8_}I_6_] possess similar crystal architectures, with only minor differences in orientation. Energy-dispersive X-ray spectroscopy (EDS) analysis (Supplementary Fig. 3) shows that the surface elements in all three samples are uniformly distributed. The experimentally determined elemental ratios are Mo:I:Cl = 6:8.84:6.22 Mo:I:Br = 6:8.19:6.29, and Mo:I = 6:15.41 (Supplementary Table 2), which are in good agreement with the theoretical values (6:8:6, 6:8:6, 6:14). These results confirm the completeness of halide substitution and verify the successful preparation of phase-pure single crystals.

**Fig. 2:**
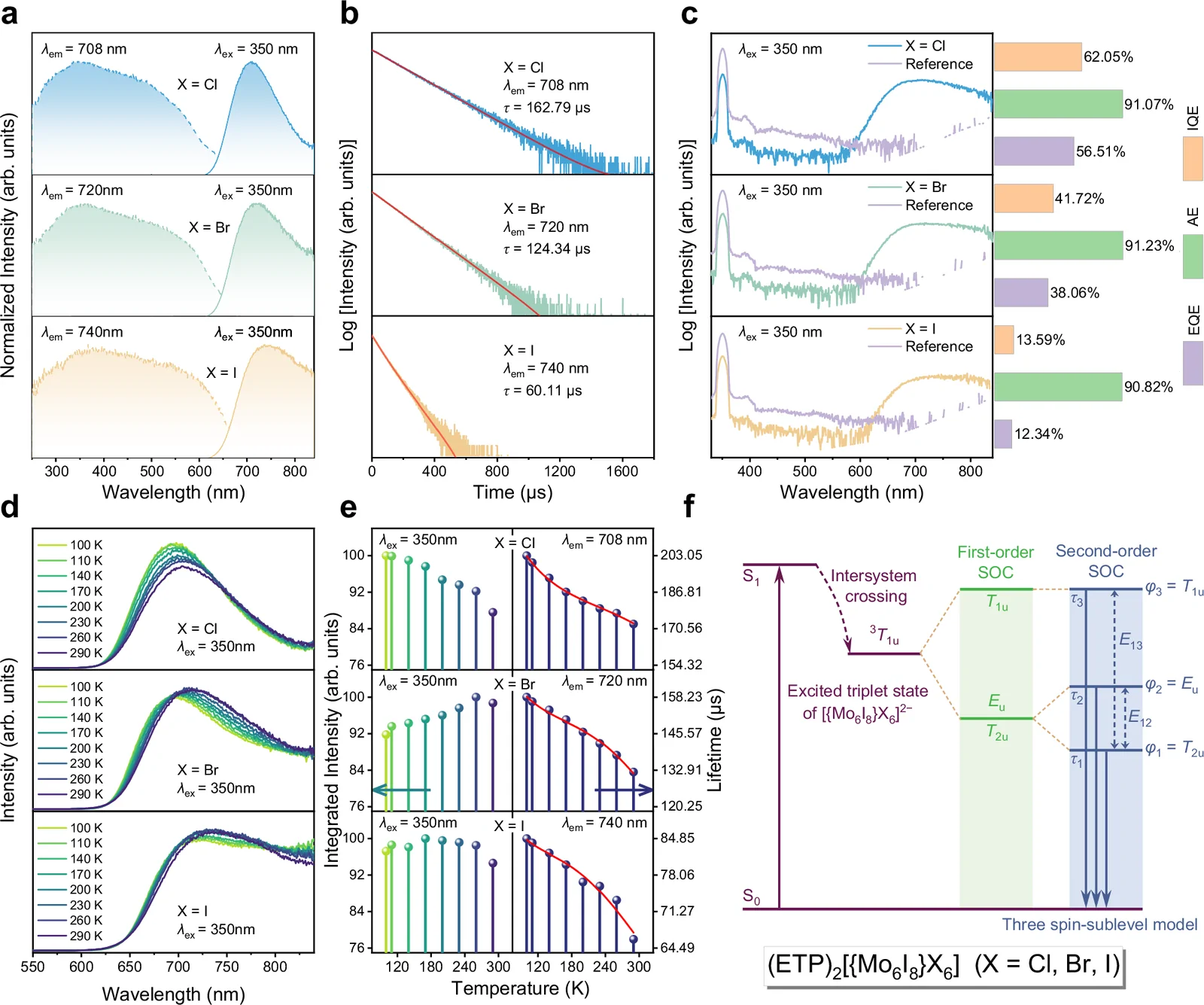
Photophysical properties of (ETP)_2_[{Mo_6_I_8_}X_6_]. **a** Photoluminescence excitation (PLE) and photoluminescence (PL) spectra of (ETP)_2_[{Mo_6_I_8_}X_6_]. **b** PL Decay curves. **c** Internal, absorbed and external quantum efficiencies (IQE, AE and EQE). **d** Temperature-dependent PL spectra. **e** Temperature dependence of integrated PL intensity and lifetime. **f** Excited triplet state three spin-sublevel models for the transition of [{Mo_6_I_8_}X_6_]^2−^ cluster. Source data are provided as a Source Data file.

All three powdered samples exhibit pronounced photoluminescence (PL) properties. As shown in Fig. 2a, their photoluminescence excitation (PLE) spectra display broad bands in the 250–700 nm range, consistent with their absorption spectra in DMF solution (Supplementary Fig. 4). This broad absorption band is attributed to charge-transfer and metal–metal/metal–ligand mixed transitions within the octahedral Mo_6_ core, and exhibits relatively high molar extinction coefficients *ε* (at 350 nm: 5606, 7207, and 24024 cm^−1^·M^−1^ for Cl, Br, and I ligands, respectively)^36^. The marked increase in molar extinction coefficient from Cl to I can be understood from the halogen-dependent orbital characteristics. As the apical ligand changes from Cl to Br and I, the halogen *p* orbitals become higher in energy and more spatially diffuse, which enhances their contribution to the frontier cluster orbitals and increases the transition dipole strength of the charge-transfer/metal–metal-related absorption bands. Therefore, the larger absorption coefficient of the I-terminated cluster is mainly associated with stronger participation of the more diffuse I 5*p* orbitals in the optical transition. Such high extinction coefficients provide a solid foundation for achieving high EQE in these materials.

Under 350 nm excitation, all three compounds show broad emissions in the red-NIR region, with peak wavelengths at 708 nm (Cl), 720 nm (Br), and 740 nm (I). The corresponding emission lifetimes are 162.79 μs, 124.34 μs, and 60.11 μs (Fig. 2b), decreasing with increasing halogen atomic number, in accordance with the heavy-atom effect associated with spin-orbit coupling (SOC) in conventional molecular transitions^37^. The emissions are attributed to the radiative ^3^T_1_ → ^1^S_0_ transitions^17,38^. Although the absorption under Xe lamp excitation during spectroscopic measurements is similar for all three samples (~91%), their quantum efficiencies differ significantly: (ETP)_2_[{Mo_6_I_8_}Cl_6_] shows the highest IQE/EQE (62.05%/56.51%), followed by Br (41.72%/38.06%) and I (13.59%/12.34%) (Fig. 2c). As the apical ligand changes from Cl to I, both the emission lifetime and efficiency decrease markedly, accompanied by red-shifted and broadened emission spectra.

To investigate the origin of this phenomenon, temperature-dependent PL measurements were first carried out on the samples over the 100–290 K range (Fig. 2d). The results show that increasing temperature generally leads to a red shift of the emission peaks for all three samples, but significant differences exist in PL intensity behavior. For (ETP)_2_[{Mo_6_I_8_}Cl_6_], the integrated PL intensity decreases monotonically with rising temperature, whereas (ETP)_2_[{Mo_6_I_8_}Br_6_] and (ETP)_2_[{Mo_6_I_8_}I_6_] exhibit an initial increase followed by a decrease. Notably, the emission lifetimes of all three samples consistently decrease with increasing temperature (Fig. 2e, and Supplementary Fig. 5), a behavior that clearly deviates from conventional Arrhenius-type thermal quenching. According to the simple large-displacement configuration coordinate diagram model under strong electron-phonon coupling, both PL intensity and decay time are expected to decrease in a consistent manner as temperature rises due to enhanced nonradiative relaxation^39,40^. In this study, the temperature-dependent PL results indicate that below room temperature, the contribution of nonradiative transitions to the PL intensity is relatively small, reflecting a high thermal activation energy or weak phonon involvement in the system. However, this is inconsistent with the observed temperature-dependent decay behaviors. Given that the luminescent centers in this system comprise a total of 20 heavy-metal and heavy-halide atoms, the excited-state dynamics must also account for strong SOC and ZFS within the cluster. These perturbations can further split the *T*_1_ level, and the corresponding transition behavior can be interpreted using the three-level model proposed by Azumi and Kitamura (Fig. 2f)^41,42^. According to their study, the ^3^*T*_1u_ state splits into multiple sublevels under SOC (Δ ≈ 620–870 cm^−1^)^41^. Considering the experimental temperature range (>100 K), the luminescent behavior of this system conforms to a three-level thermal population model^42^.

The lowest excited triplet state of the molybdenum cluster is assigned to the ^3^*T*_1u_ state arising from the (t_2g_, t_1u_) configuration, which is considered the key state responsible for its red-NIR emission. Due to the asymmetry of the cluster and the strong SOC induced by the heavy molybdenum atoms, this triplet state undergoes further splitting. Under first-order SOC, the ^3^*T*_1u_ state initially splits into the lower-energy degenerate *E*_u_ and *T*_2u_ levels, and the higher-energy *T*_1u_ and *A*_1u_ levels. Among these, the *E*_u_ and *T*_2u_ levels are degenerate, while the *A*_1u_ level is a transition-forbidden state that contributes negligibly to the emission process and is therefore not shown in the energy diagram nor considered in further discussion. To lift the accidental degeneracy between the *E*_u_ and *T*_2u_ levels, second-order SOC perturbation must be introduced. Considering both first- and second-order SOC effects, the ^3^*T*_1u_ state ultimately splits into three closely spaced sublevels, ordered from low to high energy as: *φ*_1_ = *T*_2u_, *φ*_2_ = *E*_u_, and *φ*_3_ = *T*_1u_. Correspondingly, *τ*_*n*_ (*n* = 1, 2, 3) and *E*_1n_ (*n* = 2, 3) represent the emission lifetimes of each sublevel and the energy gaps relative to the lowest level *E*_1_, respectively. The temperature-dependent emission lifetime can be described by the following equation:

1 $$\tau \left(T\right)=\,\frac{\sum {{{{\rm{g}}}}}_{n}\exp \left(-\frac{{E}_{1n}}{{k}_{B}T}\right)}{\sum \frac{{{{{\rm{g}}}}}_{n}}{{\tau }_{n}}\exp \left(-\frac{{E}_{1n}}{{{{{\rm{k}}}}}_{B}T}\right)}$$

where g_n_ denotes the degeneracy of each sublevel (*g*_1_ = 3, *g*_2_ = 2, *g*_3_ = 3), and *k*_*B*_ is the Boltzmann constant^41^. Using this model, the temperature-dependent emission lifetimes were fitted, with the red curve in Fig. 2e representing the fitting results. The corresponding parameters are listed in Table 1.

**Table 1:** Fitting parameters of (ETP)_2_[{Mo_6_I_8_}X_6_] derived from the three spin-sublevel model

Sample	*E*_13_ (cm^−1^)	*E*_12_ (cm^−1^)	*τ*_1_ (μs)	*τ*_2_ (μs)	*τ*_3_ (μs)	*R*^2^
(ETP)_2_[{Mo_6_I_8_}Cl_6_]	1346	91	241	109	7	0.99
(ETP)_2_[{Mo_6_I_8_}Br_6_]	1012	63	191	97	6	0.99
(ETP)_2_[{Mo_6_I_8_}I_6_]	799	33	119	50	5	0.97

The model-fitting results in Table 1 indicate that the splitting energies decrease as the atomic number of the ligands increases, with (ETP)_2_[{Mo_6_I_8_}Cl_6_] exhibiting the largest ZFS, Δ*E*_13_ = 1346 cm^−1^. The highly electronegative Cl ligands reduce the d-electron density of the {Mo_6_I_8_}^4+^ core, thereby increasing the effective nuclear charge (Z_eff_) of Mo, which enhances the SOC effect and enlarges the ZFS, resulting in a larger energy gap between the split levels^43^. For the low-energy levels *φ*_1_ = *T*_2u_ and *φ*_2_ = *E*_u_, radiative transitions are restricted by spin-selection rules and thus require vibronic coupling to assist the transition, leading to the longer lifetimes *τ*_1_ and *τ*_2_ obtained from the fitting. In contrast, the high-energy level *φ*_3_ = *T*_1u_ corresponds to an allowed radiative transition, resulting in a shorter fitted lifetime *τ*_3_. At low temperatures, emission predominantly originates from *φ*_1_ = *T*_2u_^42,43^. As the temperature rises, the Br and I samples with smaller splitting energies undergo thermal population, and contributions from multiple split sublevels along with their associated vibronic-assisted transitions collectively increase. This causes anomalous broadening of the spectra and enhancement of the PL integrated intensity with increasing temperature, while the proportion of emission from the high-energy sublevels grows, leading to the observed decrease in the overall measured lifetime. The crystallographic analysis further supports this conclusion (Supplementary Table 3). When Cl acts as the apical ligand, the Mo-Mo bonds are the shortest, indicating a more compact Mo_6_ octahedral core. Such a rigid structure effectively suppresses nonradiative transitions, enabling (ETP)_2_[{Mo_6_I_8_}Cl_6_] to achieve the highest quantum efficiency^13,44,45^.

**Fig. 3:**
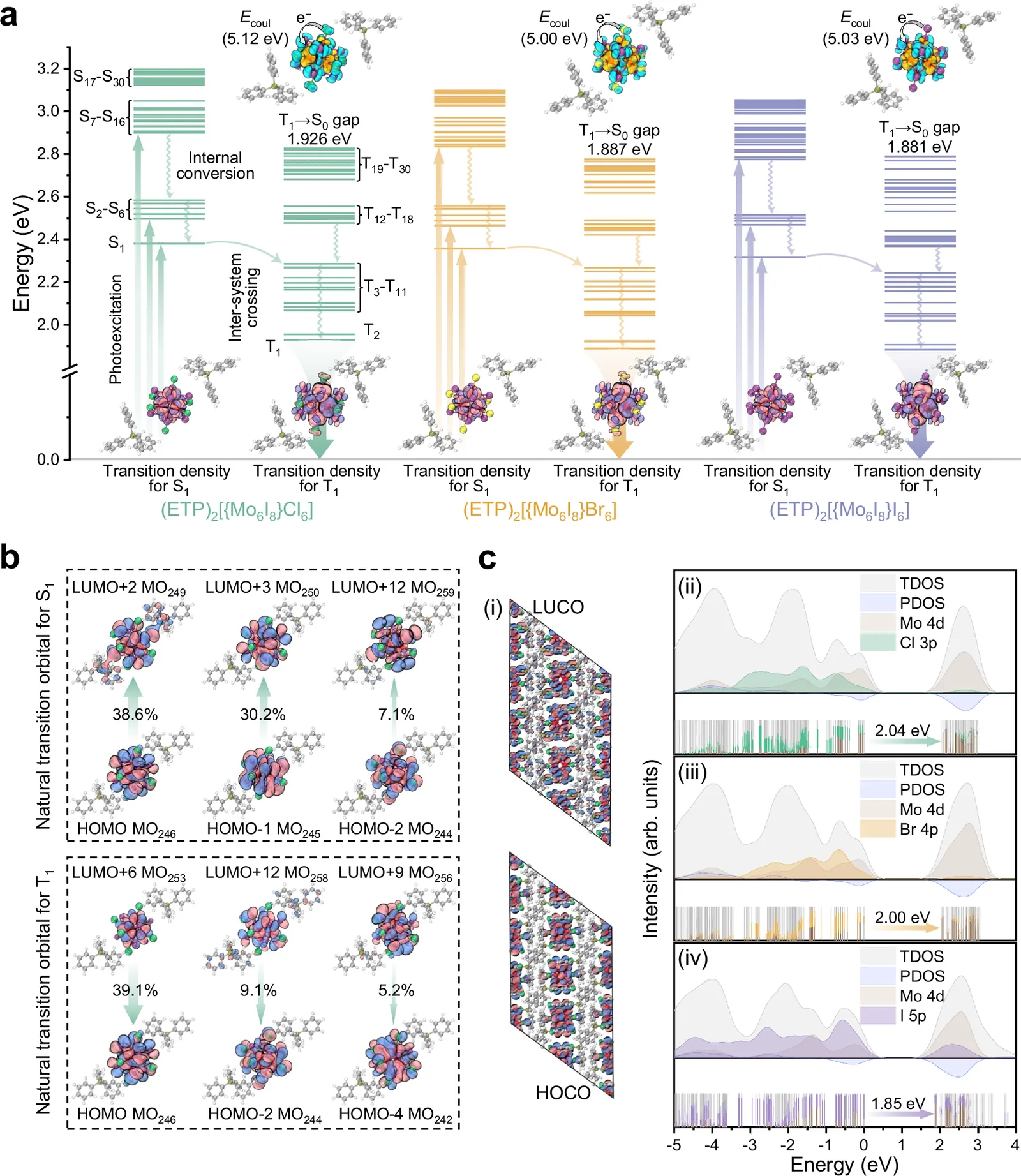
Electronic structures and excited-state characteristics of molybdenum cluster compounds. **a** Singlet and triplet excited-state energy level diagram and transition density plots for the (ETP)_2_[{Mo_6_I_8_}X_6_] clusters. **b** Natural transition orbital analysis of the S_1_ and T_1_ states. **c** The highest occupied cluster orbital and lowest unoccupied cluster orbital distributions (i), and projected density of states (PDOS) from periodic crystalline (ETP)_2_[{Mo_6_I_8_}X_6_] (ii)-(iv). Total density of states (TDOS) is also shown for comparison. Source data are provided as a Source Data file.

To further elucidate the origin of the redshift in the emission peaks of the powder samples (ETP)_2_[{Mo_6_I_8_}X_6_] (X = Cl, Br, I) as the halogen changes from Cl to I, we performed corresponding computational analyzes. Figure 3a shows the singlet and triplet excitation energies of the clusters along with their transition density distributions. The results indicate that upon photoexcitation, the system initially transitions to higher singlet excited states (S_n_), followed by internal conversion (IC) and intersystem crossing (ISC) to reach the lowest triplet state T_1_, which then emits phosphorescence via the *T*_1_ → S_0_ transition back to the ground state. Notably, the transition density of the S_1_ state is mainly localized on the {Mo_6_I_8_}^4+^ cluster core, whereas that of the *T*_1_ state extends to the apical ligands, indicating that the ligands participate in the triplet emission process. Correspondingly, the calculated T_1_ → S_0_ energy gaps slightly decrease from Cl to I (Cl: 1.926 eV, Br: 1.887 eV, I: 1.881 eV), consistent with the experimentally observed redshift of the emission spectra. The Cl-containing cluster exhibits the highest exciton binding energy (5.12 eV), which may partially explain its larger *T*_1_ → S_0_ energy gap.

We further performed Natural Transition Orbital analysis (Fig. 3b) on the S_1_ and *T*_1_ states, focusing on the three orbital pairs with the highest contributions. For S_1_, the contributions are: HOMO → LUMO + 2 (38.6%), HOMO-1 → LUMO + 3 (30.2%), and HOMO-2 → LUMO + 12 (7.1%). For T_1_, the contributions are: HOMO → LUMO + 6 (39.1%), HOMO-2 → LUMO + 12 (9.1%), and HOMO-4 → LUMO + 9 (5.2%). Notably, all *T*_1_ orbitals are localized on the [{Mo_6_I_8_}X_6_]^2-^ cluster, whereas the highest-contributing S_1_ orbitals (LUMO + 2) also involve the organic ETPP^+^ cation, indicating that the organic cation participates in light absorption under photoexcitation, whereas the *T*_1_ phosphorescence is primarily due to the [{Mo_6_I_8_}X_6_]^2-^ cluster. To further understand the halogen-dependent optical shift, we analyzed the electronic structure via projected density of states (PDOS) calculations (Fig. 3c). For all three compounds (X = Cl, Br, I), the valence band maximum (highest occupied cluster orbital, HOCO) and conduction band minimum (lowest unoccupied cluster orbital, LUCO) are mainly composed of Mo 4*d* and halogen *p* orbitals, indicating strong Mo-X orbital hybridization. Specifically, Mo 4*d* orbitals couple with apical halogen *p* orbitals (Cl 3*p*, Br 4*p*, I 5*p*) to form the frontier orbitals. Both HOCO and LUCO are significantly influenced by this Mo-X mixing, meaning that while the band edges are cluster-centered, they are strongly affected by Mo-X bonding. As the halogen changes from Cl to I, the energy and diffusivity of the halogen *p* orbitals increase (due to lower electronegativity), raising the HOCO level and slightly shifting the LUCO, leading to a narrower bandgap. This trend explains the emission redshift from 708 nm (Cl) to 740 nm (I). Essentially, the stronger Mo-Cl bonds (with lower Cl 3*p* orbital energies) stabilize the HOCO and widen the gap, whereas Mo-I bonds (with higher I 5*p* orbital energies) raise the HOCO and reduce the gap. Therefore, the degree of Mo-halogen bonding directly determines the bandgap and optical transition energy of these cluster compounds, explaining the halogen-dependent emission behavior.

The reason why the powder sample of (ETP)_2_[{Mo_6_I_8_}Cl_6_] does not achieve 100% IQE at room temperature is likely related to quenching of the triplet emission by ^1^O_2_ in air as well as ACQ of the sample itself. If the clusters are uniformly dispersed in a medium, the emission lifetime and IQE of the sample are expected to increase significantly^38,46^. To verify this hypothesis, the powder sample was dissolved in DMF and deoxygenated, resulting in an increase of the emission lifetime from 162.79 to 196.28 μs (Supplementary Fig. 6a). Subsequently, upon exposure to air, the molybdenum clusters interacted with oxygen, causing the lifetime to drop sharply to 8.07% of its initial value within 6 min (Supplementary Fig. 6b), while the PL intensity decreased to 4.86% (Supplementary Fig. 7). To further investigate the nature of this quenching process, nitrogen was bubbled through the solution after it had been fully exposed to air to remove oxygen (Supplementary Fig. 8a). As shown in Supplementary Fig. 8b, under UV illumination, the red emission gradually recovered as nitrogen was introduced. More quantitatively, after 180 s of nitrogen bubbling, the emission intensity recovered to 98.06% of the initial value (Supplementary Fig. 9). This experiment clearly demonstrates that in an oxygen-free solution, both ^1^O_2_ quenching and ACQ are effectively suppressed, and that ^1^O_2_ quenching is a highly reversible process.

**Fig. 4:**
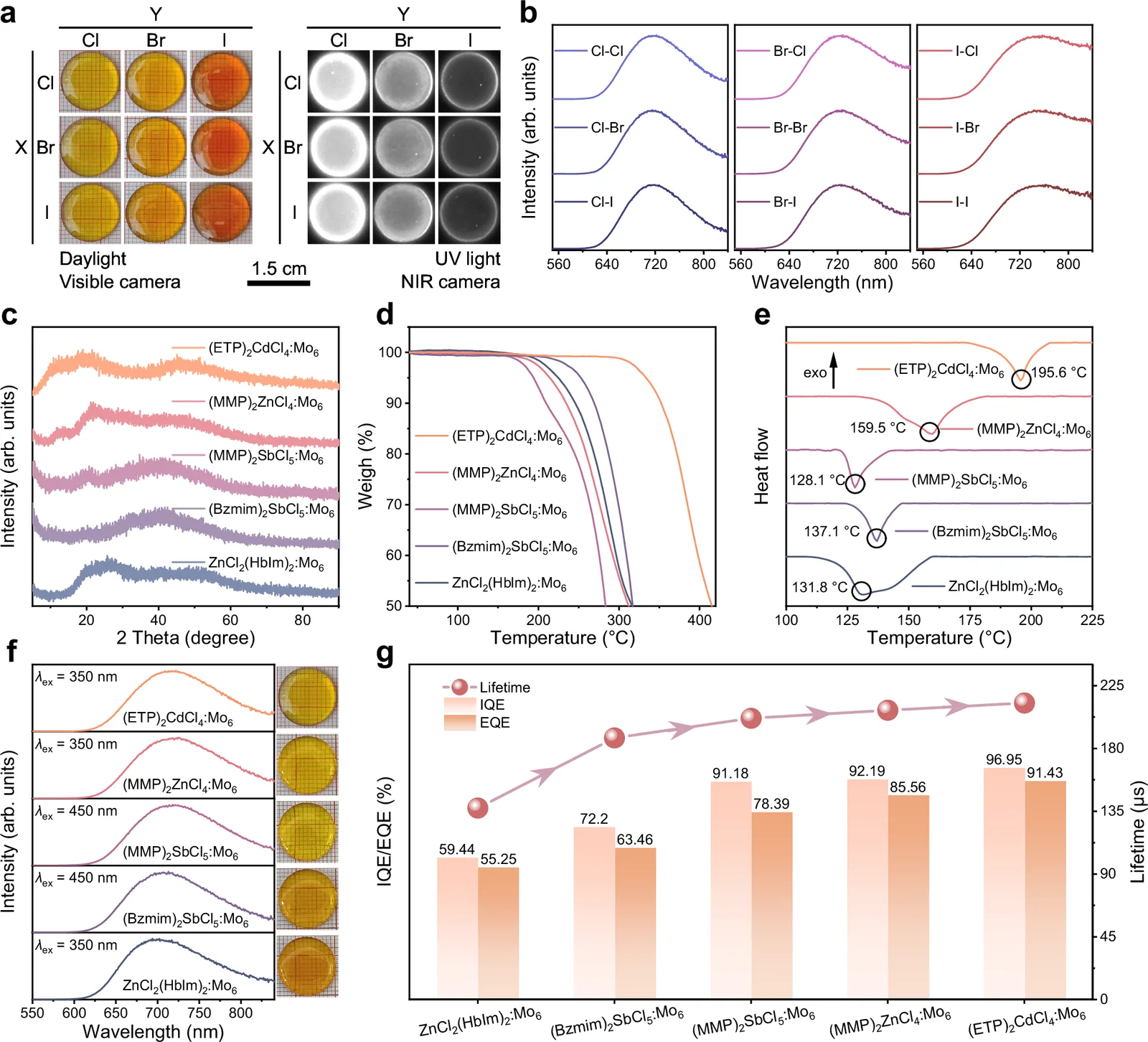
Incorporation of molybdenum cluster emitters into molecular hybrid halide glasses. **a** Photographs of glass samples under daylight recorded with a visible camera and under ultraviolet excitation recorded with a near-infrared camera. **b** PL spectra of (ETP)_2_CdY_4_:[{Mo_6_I_8_}X_6_]^2−^ glass, abbreviated as Y-X. **c** XRD patterns, **d** Thermogravimetric curves, and **e** Differential scanning calorimetry curves of five glass matrices doped with (ETP)_2_[{Mo_6_I_8_}Cl_6_]. **f** PL spectra and corresponding photographs. **g** Lifetimes, IQEs and EQEs. Source data are provided as a Source Data file.

Our previous studies have shown that dispersing molybdenum clusters in a hybrid glass matrix can effectively suppress the quenching effects described above, thereby improving quantum yield^34^. However, the specific influence of the glass matrix on the luminescence behavior of the clusters has not yet been systematically investigated. By mixing ETPPY:CdY_2_:(ETP)_2_[{Mo_6_I_8_}X_6_] in a mass ratio of 2:1:0.17%, followed by heating, melting, and cooling, we successfully prepared transparent (ETP)_2_CdY_4_:[{Mo_6_I_8_}X_6_]^2−^ glasses (abbreviated as Y-X, Y = Cl, Br, I, and X = Cl, Br, I). The nine resulting glass samples exhibited a key phenomenon: for a given matrix (i.e., fixed Y), despite different initial cluster ligands (X), all glasses showed the same bulk color, and under UV excitation, their NIR emission brightness was identical (Fig. 4a, and Supplementary Fig. 10). XRD measurements (Supplementary Fig. 11) confirmed that doping with different molybdenum clusters did not alter the amorphous structure of the glass matrix, as diffraction patterns for the same matrix were completely consistent. More importantly, spectroscopic tests revealed that for the same matrix, regardless of the initially doped cluster, the resulting emission spectra (Fig. 4b), luminescence lifetimes, and quantum efficiencies were highly similar (Supplementary Figs. 12–15). These results strongly indicate that during glass formation, the original ligands X^−^ of the doped molybdenum clusters are completely replaced by the high-concentration Y^−^ ions in the matrix. Supplementary Fig. 16 visually illustrates this behavior using (ETP)_2_CdCl_4_ (Y = Cl) glass as an example. Consequently, the emission properties are ultimately determined by the formation of [{Mo_6_I_8_}Y_6_]^2−^ within the glass matrix, rather than by the initially doped clusters [{Mo_6_I_8_}X_6_]^2−^. This mechanism is further supported by the observed trend: as the matrix halide changes from Cl to Br to I, the glass PL spectra redshift and broaden, while lifetimes and quantum efficiencies decrease (Supplementary Table 4), consistent with the trends observed in the powder samples when the apical ligands are varied. According to this mechanism, compared with the corresponding powder samples, the Cl-Cl, Br-Br, and I-I series of glasses show significantly enhanced lifetimes and quantum efficiencies (Supplementary Table 5). This confirms that the glass matrix effectively disperses the molybdenum clusters, suppressing both aggregation and ^1^O_2_ quenching, and that increased sample thickness also extends the optical path, enhancing absorption of the excitation light.

Further investigations demonstrated that this dispersion effect is also applicable to other hybrid glass systems. When the same concentration of (ETP)_2_[{Mo_6_I_8_}Cl_6_] was doped into (MMP)_2_ZnCl_4_, (MMP)_2_SbCl_5_^47^, (Bzmim)_2_SbCl_5_^48^, and ZnCl_2_(HbIm)_2_^49^ glasses (MMP: (methoxymethyl)triphenylphosphonium, Bzmim: 1-benzyl-3-methylimidazolium, HbIm: benzimidazole), the resulting samples (MMP)_2_ZnCl_4_:Mo_6_, (MMP)_2_SbCl_5_:Mo_6_, (Bzmim)_2_SbCl_5_:Mo_6_, and ZnCl_2_(HbIm)_2_:Mo_6_, exhibited amorphous characteristics, similar to the previously studied (ETP)_2_CdCl_4_:Mo_6_ (Cl-Cl) glass (Fig. 4c), indicating that doping did not disrupt the glass structures. Thermogravimetric analysis (TGA) and Differential Scanning Calorimetry (DSC) measurements (Fig. 4d, e, and Supplementary Table 6) showed that the decomposition temperatures (*T*_d_) of all samples were higher than their melting points (*T*_m_), meeting the thermal stability requirements for melt-quenching glass preparation^27^. The emission peaks of all five glasses were located in the NIR region (Fig. 4f), and their bulk colors and emission intensities were similar, confirming that the emission centers in all cases were [{Mo_6_I_8_}Cl_6_]^2−^. To further verify the singularity of the emission center, PL spectra were measured under various excitation wavelengths (300–500 nm, in 25 nm intervals) for each sample (Supplementary Fig. 17). The results revealed that in most matrices, only the characteristic emissions from the molybdenum clusters were observed. Although in some excitation conditions the [SbCl_5_]^2−^ matrix introduced additional peaks, excitation at 450 nm produced emission solely from the molybdenum cluster centers. This demonstrates that [{Mo_6_I_8_}Cl_6_]^2−^ acts as a stable and single dominant emission center across different glass environments. Raman spectra (Supplementary Fig. 18) showed that the characteristic peak at 255 cm^−1^ in the (ETP)_2_CdCl_4_:Mo_6_ glass had the narrowest full-width at half-maximum among the compared systems. Since Raman peak width reflects structural vibration heterogeneity, this indicates that this glass matrix possesses the highest structural rigidity.

Among these glasses, (ETP)_2_CdCl_4_:Mo_6_ also exhibited the best overall performance, achieving the highest emission lifetime, IQE, and EQE (Fig. 4g, and Supplementary Figs. 19–21). This is attributed to the matrix’s higher rigidity, which effectively suppresses cluster vibrations and nonradiative transitions, while its weak competitive absorption of excitation light allows more energy to excite the molybdenum clusters. Repeated measurements on independently prepared (ETP)_2_CdCl_4_:Mo_6_ glass samples further confirm the reproducibility of this high-efficiency emission. Based on 18 repeated measurements, the average IQE, absorption efficiency and EQE are 96.85 ± 1.00%, 93.11 ± 0.43%, and 90.18 ± 1.07%, respectively (Supplementary Tables 7 and 8). Temperature-dependent PL measurements (Supplementary Fig. 22) provide direct evidence of low nonradiative rates: from 80 K to 290 K, the emission intensity only slightly decreased from 100% to 97.34%. When the temperature rises above room temperature, emission intensity drops sharply, likely due to enhanced nonradiative relaxation from increased molecular vibrations of the organic components. The 96.95% IQE achieved in this work compares favorably with those of previously reported high-performance {Mo_6_I_8_}^4+^ cluster systems, as summarized in Supplementary Table 9.

**Fig. 5:**
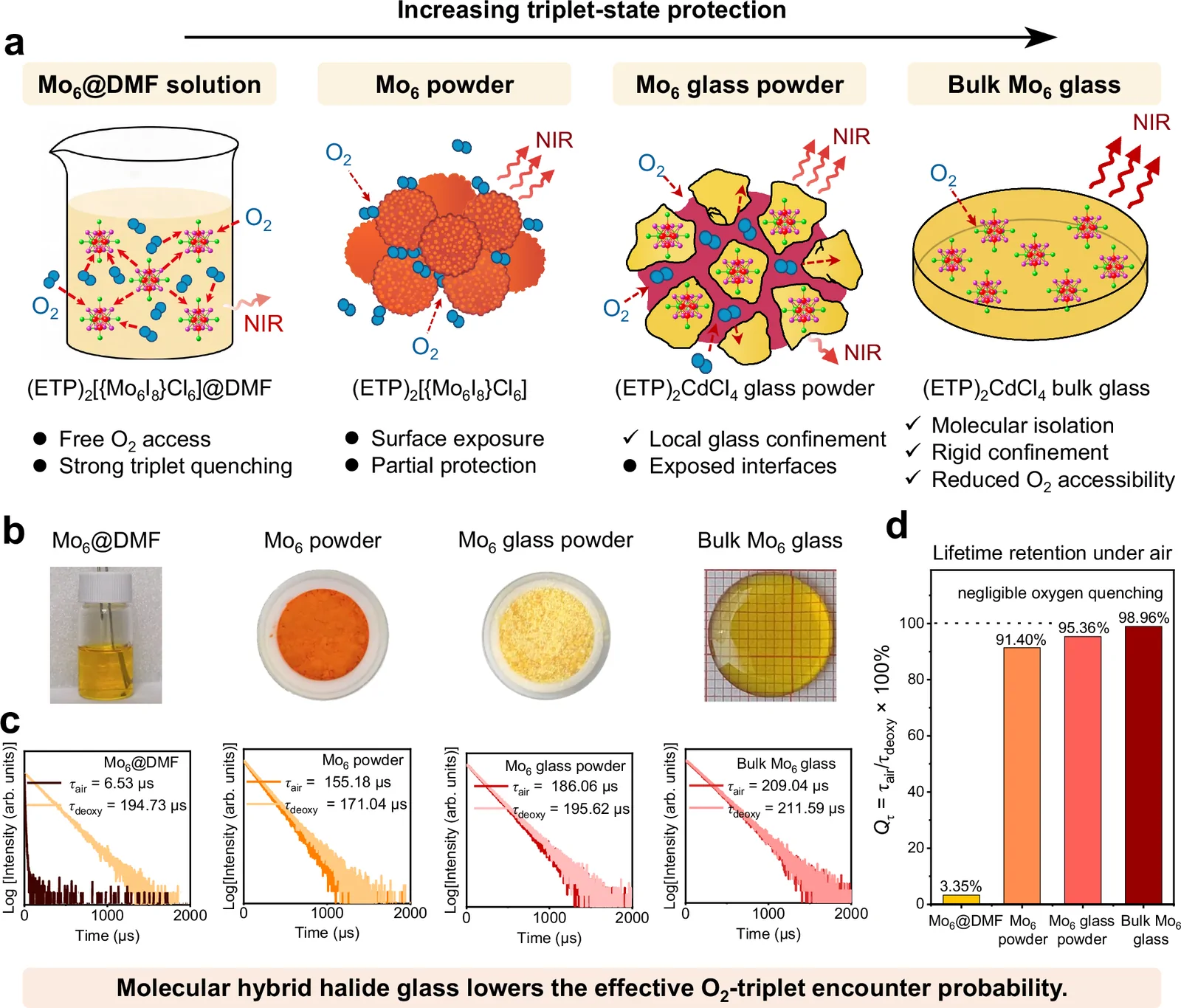
Environment-dependent oxygen-quenching resistance of Mo_6_ cluster emitters. **a** Schematic illustration of oxygen accessibility and triplet-state protection in DMF solution, molecular powder, glass powder and bulk molecular hybrid halide glass. **b** Representative photographs of the corresponding samples. **c** PL decay curves measured under air and corresponding low-oxygen conditions. For the DMF solution, the low-oxygen condition was obtained by N_2_ bubbling; for the powder, glass powder and bulk glass samples, it was obtained by vacuum evacuation followed by dry-N_2_ backfilling. **d** Lifetime retention ratios showing a pronounced increase from Mo_6_@DMF to bulk Mo_6_ glass. Source data are provided as a Source Data file.

These results demonstrate that dispersing molybdenum clusters in a hybrid glass matrix is a generalizable strategy, with a physical process analogous to cluster dissolution in polar ionic liquids followed by glass formation. To further clarify why the optimized molecular hybrid halide glass shows limited oxygen quenching, we quantitatively compared the oxygen-quenching resistance of the same Mo_6_ emitter in different physical environments (Fig. 5a). Based on the lifetime form of the Stern-Volmer quenching relationship^50^, we defined a lifetime retention ratio, *Q*_τ_ = τ_air_/τ_deoxy_ × 100%, where τ_deoxy_ denotes the lifetime measured under the corresponding low-oxygen condition. For the DMF solution, the low-oxygen state was obtained by N_2_ bubbling; for the solid powder, glass powder and bulk glass samples, the low-oxygen state was obtained by vacuum evacuation followed by dry-N_2_ backfilling. A larger *Q*_τ_ value indicates that the long-lived Mo_6_ triplet excited state is less affected by oxygen under ambient air.

As shown in Fig. 5b–d, molecularly dispersed (ETP)_2_[{Mo_6_I_8_}Cl_6_] in DMF exhibits a very low *Q*_τ_ of 3.35%, confirming that the Mo_6_ triplet excited state is highly vulnerable to oxygen-induced dynamic quenching when freely accessible in solution. The pure (ETP)_2_[{Mo_6_I_8_}Cl_6_] powder shows a much higher Qτ of 91.40%, indicating partial solid-state restriction of oxygen access, although surface exposure, defects and cluster-cluster contacts can still contribute to triplet-state deactivation. The (ETP)_2_CdCl_4_:Mo_6_ glass powder further increases *Q*_τ_ to 95.36%, showing that the vitrified matrix provides local protection to the embedded Mo_6_ emitters even after grinding. The intact bulk (ETP)_2_CdCl_4_:Mo_6_ glass exhibits the highest *Q*_τ_ of 98.96%, demonstrating that the complete glass body provides the most effective protection against oxygen-induced triplet quenching under ambient air.

This stepwise increase in oxygen-quenching resistance from the DMF solution to the molecular powder, glass powder, and bulk glass indicates that the oxygen-quenching suppression of the present system is not simply an intrinsic property of the Mo_6_ cluster or a passive blocking effect of any solid state. Instead, the molecular hybrid halide glass suppresses oxygen quenching through coupled chemical and structural effects. During glass formation, the halide-rich ionic melt promotes molecular-level cluster dispersion and matrix-dictated halide-environment redistribution, as evidenced by the convergence of emission spectra, lifetimes and quantum efficiencies for different initial cluster ligands within the same glass matrix. After vitrification, the rigid amorphous matrix freezes the dispersed emissive centers, suppresses cluster-cluster interactions and vibrationally assisted nonradiative relaxation, and markedly lowers the effective encounter probability between molecular oxygen and the long-lived Mo_6_ triplet excited state. Therefore, the glass matrix provides triplet-state protection through molecular isolation, rigid confinement, reduced oxygen accessibility and matrix-dictated emissive-center formation.

As shown in Supplementary Figs. 23 and 24, after exposure to air for 180 min, the emission lifetime and intensity of (ETP)_2_CdCl_4_:Mo_6_ glass decreased by less than 1%, indicating much higher air stability than that of the solution-based system. In addition, continuous 350 nm UV irradiation causes negligible changes in the PL spectra, emission lifetime and integrated intensity of the optimized glass (Supplementary Fig. 25), further confirming its photostability under application-relevant excitation conditions.

**Fig. 6:**
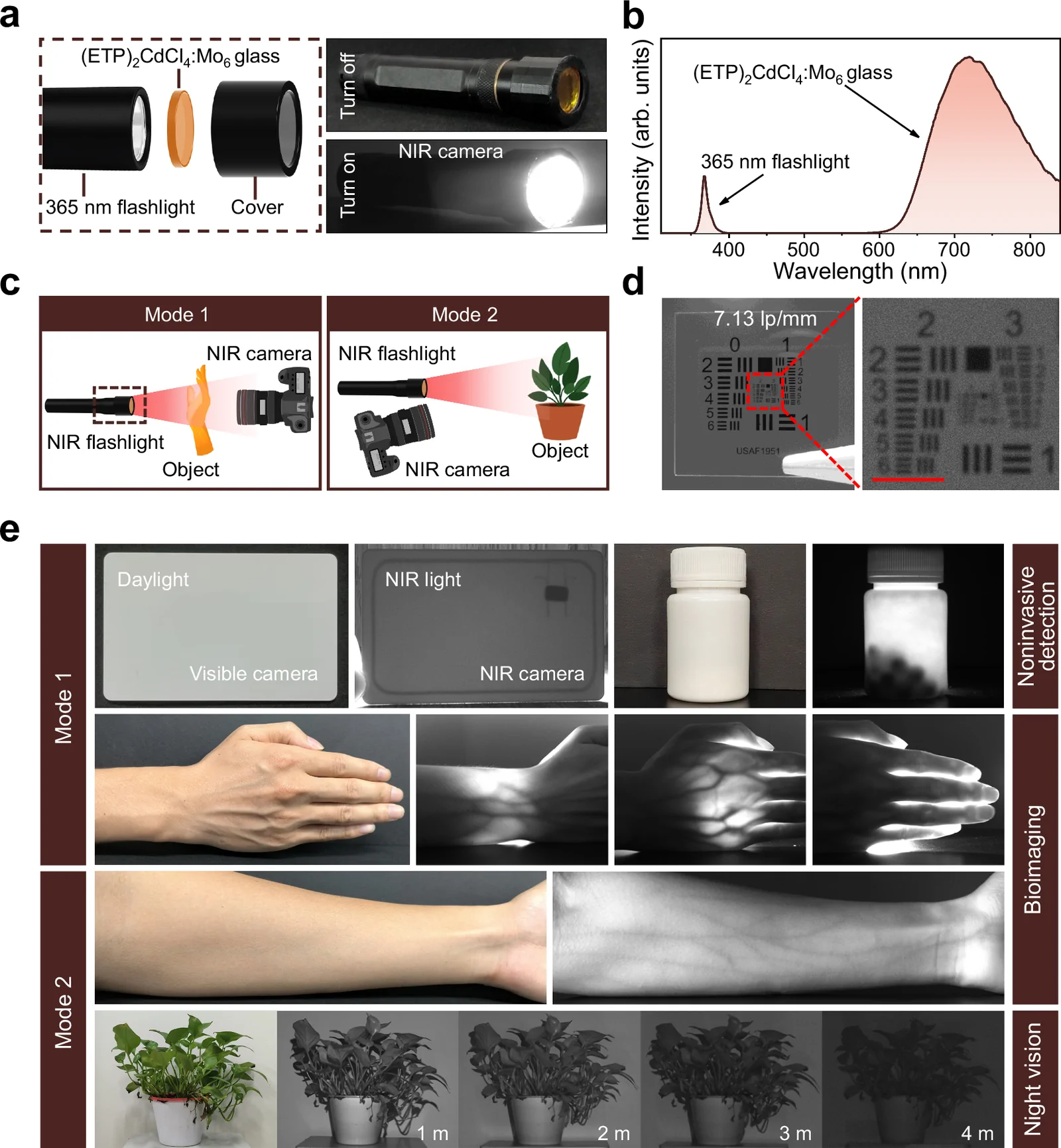
Compact near-infrared illumination and imaging enabled by the molybdenum cluster glass. **a** Schematic illustration of the near-infrared flashlight assembly and photographs of the flashlight in the off and on states. **b** Output emission spectrum of the flashlight in the on state. **c** Schematic illustration of transmitted-light imaging (Mode 1) and scattered-light imaging (Mode 2), in which the near-infrared camera captures the transmitted or scattered signals when the flashlight illuminates an object. The hand, camera and potted-plant vector elements in **c** were adapted from Pixabay under the Pixabay Content License. **d** Imaging resolution evaluated using a USAF 1951 resolution target. **e** Demonstrations of nondestructive detection, bioimaging and night-vision applications. The labels “1 m”, “2 m”, “3 m” and “4 m” indicate imaging distances under dark conditions. Source data are provided as a Source Data file.

To further evaluate the practical application potential of the (ETP)_2_CdCl_4_:Mo_6_ glass as a NIR light source, we integrated it into a portable UV-excited flashlight (Fig. 6a). Specifically, custom glass samples with a diameter of approximately 2 cm were prepared, and a black housing was 3D-printed to fit snugly around each sample. A quartz window of matching dimensions was installed to securely position the glass in front of a 365 nm UV light source, ensuring efficient UV excitation and stable NIR emission. Upon activation of the UV source, the (ETP)_2_CdCl_4_:Mo_6_ glass produced strong and stable NIR emission, which could be clearly captured by an NIR camera. The corresponding emission spectrum (Fig. 6b) shows that the peak lies within the NIR range, confirming the material’s efficient energy conversion and broad emission coverage. Based on this high-performance light source, we developed two imaging modes to demonstrate its application versatility (Fig. 6c). Mode 1 is transmission imaging, suitable for nondestructive inspection of biological tissues or thin-layer samples, leveraging the high penetration depth of NIR light to visualize internal structures. Mode 2 is reflection/scattering imaging, which collects scattered signals from the sample surface to reveal edge contours and surface textures, making it suitable for complex morphologies or distant targets^51,52^. To quantify the imaging system’s performance, a USAF 1951 resolution target was used for calibration (Fig. 6d, and Supplementary Table 10). Briefly, the NIR camera was positioned approximately 10 cm from the USAF 1951 target, and the NIR flashlight was placed approximately 10 cm above the target under dark conditions. We further examined the influence of glass thickness on the imaging performance. (ETP)_2_CdCl_4_ glasses with thicknesses of 1.51, 2.62, 4.33 and 5.74 mm were prepared by varying the precursor loading. No obvious degradation in imaging resolution was observed within this thickness range (Supplementary Fig. 26). Finally, the system achieved a spatial resolution of up to 7.13 lp/mm, clearly resolving fine microstructures and confirming its imaging performance.

In proof-of-concept experiments, the NIR flashlight was successfully applied in multiple scenarios (Fig. 6e). For materials inspection, it could clearly visualize tablets to detect residual particles and reveal embedded chip structures in cards, demonstrating potential for nondestructive testing. In biological imaging, the low scattering and weak tissue absorption of NIR light enabled visualization of subcutaneous blood vessels. Moreover, under completely dark conditions without ambient light, the system still achieved clear imaging over distances of 1–4 m, highlighting its potential for night-vision applications. In summary, the NIR flashlight constructed from (ETP)_2_CdCl_4_:Mo_6_ glass exhibits practical potential in nondestructive testing, biomedical imaging and night-vision technologies^53–55^.

In summary, a molecular hybrid halide glass strategy has been developed to convert oxygen-sensitive Mo_6_ molecular triplet emitters into air-stable, processable and highly efficient bulk NIR-emitting solids. Through high-temperature ionic-liquid-assisted ligand exchange, isostructural (ETP)_2_[{Mo_6_I_8_}X_6_] (X = Cl, Br and I) clusters were prepared, and the role of apical halide chemistry in regulating Mo-X bonding, frontier-orbital composition, ZFS and triplet-state radiative dynamics was clarified. The highest emission efficiency was obtained for the Cl-terminated cluster, which is attributed to stronger Mo-Cl bonding and a more rigid cluster core that suppress nonradiative relaxation. After the optimized cluster was embedded into (ETP)_2_CdCl_4_ glass, molecular-level dispersion, matrix-dictated halide-environment redistribution and rigid amorphous confinement were achieved, thereby reducing cluster-cluster interactions, vibrational deactivation and oxygen-induced triplet quenching. As a result, an IQE of 96.95% and an EQE of 91.43% were achieved in air for the optimized glass, together with good reproducibility, ambient stability and photostability. Air/low-oxygen lifetime comparisons further confirmed that the strongest triplet-state protection was provided by the bulk glass through lowering the effective encounter probability between molecular oxygen and the long-lived Mo_6_ excited state. The optimized glass was further integrated into a UV-excited NIR flashlight, and its potential for nondestructive inspection, biological imaging and night-vision applications was demonstrated. This work establishes molecular hybrid halide glass as an ionically adaptive and structurally confining platform for translating oxygen-sensitive molecular emitters into robust solid-state NIR light sources.

## Methods

### Materials

(Ethyl)triphenylphosphonium chloride (C_20_H_20_ClP, abbreviated as ETPPCl, 98%), (ethyl)triphenylphosphonium bromide (C_20_H_20_BrP, abbreviated as ETPPBr, 98%), (ethyl)triphenylphosphonium iodide (C_20_H_20_IP, abbreviated as ETPPI, 98%), (methoxymethyl)triphenylphosphonium chloride (C_20_H_20_ClOP, abbreviated as MMPPCl, 99%), 1-benzyl-3-methylimidazolium chloride (C_11_H_13_ClN_2_, abbreviated as BzmimCl, 97%), benzimidazole (C_7_H_6_N_2_, abbreviated as HbIm, 99%), cadmium chloride (CdCl_2_, 99.99%), cadmium bromide (CdBr_2_, 99.99%), cadmium iodide (CdI_2_, 99%), zinc chloride (ZnCl_2_, 99.99%), antimony trichloride (SbCl_3_, 99%), methyl alcohol (CH_3_OH, 99.9%), N,N-dimethylformamide (C_3_H_7_NO, abbreviate as DMF, 99.8%) and diethyl ether (C_4_H_10_O, abbreviate as Et_2_O, 99.5%) were purchased from Adamas. Molybdenum iodide (MoI_2_ or Mo_6_I_12_, 99%) was purchased from Macklin. All reagents and solvents were used without further purification.

### Synthesis of (ETP)_2_[(Mo_6_I_8_)X_6_] (X = Cl, Br, I) crystals

A mixture of finely ground 0.2 g MoI_2_ and 20 g ETPPCl was heated at 290 °C until a molten liquid was formed and stirred for 1 h. After cooling to room temperature, the mixture was washed three times with methanol to remove excess ETPPCl. The resulting crude product was dissolved in 7 mL DMF, centrifuged, and the supernatant was filtered through a 0.22 μm filter head. The filtrate was left to evaporate in a glovebox for several weeks to afford single crystals of (ETP)_2_[{Mo_6_I_8_}Cl_6_]. Alternatively, slow addition of the solution into excess Et_2_O yielded polycrystalline powder of (ETP)_2_[{Mo_6_I_8_}Cl_6_]. The syntheses of (ETP)_2_[{Mo_6_I_8_}Br_6_] and (ETP)_2_[{Mo_6_I_8_}I_6_] were performed in a similar manner, with reaction temperatures of 270 °C and 250 °C, respectively. Only 10 g of ETPPI was required for the synthesis of (ETP)_2_[{Mo_6_I_8_}I_6_].

### Synthesis of (ETP)_2_CdY_4_:[{Mo_6_I_8_}X_6_]^2-^ Glasses. (X, Y = Cl, Br, I)

A mixture of ETPPY, CdY_2_, and (ETP)_2_[{Mo_6_I_8_}X_6_] in a molar ratio of 2:1:0.17% was thoroughly ground and homogenized. The mixture was transferred into a glass vessel and heated until a transparent molten liquid was formed. The melt was rapidly poured into a silicone mold and cooled to room temperature to afford transparent (ETP)_2_CdY_4_:[{Mo_6_I_8_}X_6_]^2-^ glasses. When Y = Cl, Br, and I, the heating temperatures were 280 °C, 260 °C, and 240 °C, respectively. All procedures were carried out in a glovebox.

### Synthesis of (MMP)_2_ZnCl_4_: Mo_6_ glass

A mixture of MMPPCl, ZnCl_2_, and (ETP)_2_[{Mo_6_I_8_}Cl_6_] in a molar ratio of 2:1: 0.17% was thoroughly ground and homogenized. The mixture was transferred into a glass vessel and heated at 165 °C until a transparent molten liquid was formed. The melt was rapidly poured into a silicone mold and cooled to room temperature to afford transparent (MMP)_2_ZnCl_4_: Mo_6_ glass. The syntheses of (MMP)_2_SbCl_5_, (Bzmim)_2_SbCl_5_, and ZnCl_2_(HbIm)_2_ glasses were carried out in a similar manner as described above, except that the corresponding organic components and metal halides were used. The synthesis temperatures were 140 °C, 160 °C, and 140 °C, respectively.

### Structural characterization

XRD measurements were carried out on a Rigaku SmartLab SE powder X-ray diffractometer using monochromatic Cu-Kα radiation (*λ* = 1.5406 Å) at 40 kV and 30 mA. Diffraction patterns were scanned at room temperature in steps of 0.02° over an angular range of 5-90° (2θ). Single-crystal data collection of (ETP)_2_[(Mo_6_I_8_)X_6_] (X = Cl, Br, I) were performed on a Bruker Apex II CCD diffractometer at 298 K using Mo-Kα radiation (λ = 0.71073 Å). Photoluminescence (PL) and photoluminescence excitation (PLE) spectra at room temperature were recorded on an FLS1000 photoluminescence spectrophotometer (Edinburgh Instruments, UK) equipped with a continuous 450 W xenon lamp as the excitation source. PLQY values were recorded using a Teflon integrating sphere coupled with the FLS1000. Decay curves were monitored using the FLS1000 spectrophotometer, excited either by a flash xenon lamp with microsecond pulses. Temperature-dependent PL properties were measured using the same spectrophotometer with an open-cycle liquid-nitrogen cooling cryostat (NCV30-2W-H, East Changing, China). Diffuse reflection spectra of the samples were measured on a UV-3600i Plus UV-vis-NIR spectrophotometer (Shimadzu, Japan), with white BaSO_4_ used for absorption correction. Thermogravimetric/differential scanning calorimetry (TG/DSC) analyzes were performed on a Netzsch STA449F3 apparatus in an argon atmosphere.

### Computational methods

The periodic structures of (ETP)_2_[{Mo_6_I_8_}X_6_] (X = Cl, Br, I) were first optimized using the CP2K package^56^ with the PBE functional^57^ and Grimme’s D3 dispersion correction^58^. Goedecker–Teter–Hutter (GTH) pseudopotentials were used in combination with DZVP-MOLOPT-SR-GTH basis sets^59,60^ for all elements. The *k*-point meshes were 1 × 2 × 2 (Cl), 3 × 1 × 2 (Br), and 2 × 1 × 2 (I), respectively. Following geometry optimization, single-point energy calculations were performed on each supercell and the resulting wavefunctions were used for density of states (DOS) and projected DOS (PDOS) analyzes. The PDOS results revealed that both the highest occupied crystal orbitals (HOCO) and the lowest unoccupied crystal orbitals (LUCO) are primarily composed of hybridized Mo 4*d* and halide *p* orbitals (Cl 3*p*, Br 4*p*, I 5*p*). This strong Mo-X orbital mixing governs the band edge positions and explains the halide-dependent bandgap narrowing from Cl to I.

To probe the local excited-state properties, a molecular cluster fragment was extracted from the optimized periodic structures, consisting of the [{Mo_6_I_8_}X_6_] core and two nearby ETP^+^ countercations. Excited-state calculations were carried out using the Gaussian software^61^, employing time-dependent DFT (TD-DFT)^62^ at the PBE0 level^63^ of theory with the SMD^64^ implicit solvation model (solvent: toluene). In which, the 30 singlet and triplet excited states were evaluated. For Mo, I, and Br, the LanL2DZ effective core potential (ECP) basis set^65–67^ was used along with added polarization functions to improve the accuracy of excited-state orbital responses. Specifically, Mo received one *f*-type function (exponent 1.034), I one *d*-type (exponent 0.294), Br one *d*-type (exponent 0.434) and Cl one *d*-type (exponent 0.648). These custom basis functions were selected based on literature precedents^68^. Light elements (C, H, P) were treated using the 6-31G* basis set^69^. Natural transition orbital (NTO) analyzes were used to extract the dominant transitions contributing to the S_1_ and *T*_1_ states, and transition density plots were generated to visualize orbital localization, providing insight into ligand vs. cluster contributions to absorption and emission.

## Supplementary information

Supplementary Information

Transparent Peer Review file

## Source data

Source Data

## Data Availability

The data supporting the findings of this study are available within the Article, Supplementary Information and Source Data files. Source data are provided with this paper. Crystallographic data newly generated in this study for (ETP)_2_[{Mo_6_I_8_}Cl_6_] and (ETP)_2_[{Mo_6_I_8_}Br_6_] have been deposited at the Cambridge Crystallographic Data Centre under deposition numbers CCDC 2477364 and 2477363, respectively. The previously reported crystallographic data for (ETP)_2_[{Mo_6_I_8_}I_6_], used here for comparison, are available from the Cambridge Crystallographic Data Centre under deposition number CCDC 2425267 (DOI: 10.5517/ccdc.csd.cc2mdpf5). These data can be obtained free of charge from the Cambridge Crystallographic Data Centre. Source data are provided with this paper.

## Code availability

No custom code was developed for this study. All calculations were performed using the software packages and parameters described in the Methods section.

## References

[1] Miao, S., Liang, Y. & Wang, X. J. Recent advances and emerging applications of lanthanide/transition-metal-ion-doped short-wave infrared luminescent materials. *Adv. Opt. Mater.* **13**, e01928 (2025).

[2] Liu, D., Dang, P., Zhang, G., Lian, H., Li, G. & Lin, J. Near-infrared emitting metal halide materials: luminescence design and applications. *InfoMat* **6**, e12542 (2024).

[3] Qiao, J., Zhou, G., Zhou, Y., Zhang, Q. & Xia, Z. Divalent europium-doped near-infrared-emitting phosphor for light-emitting diodes. *Nat. Commun.* **10**, 5267 (2019). 31748595 10.1038/s41467-019-13293-0 PMC6868216

[4] Kwak, K., Thanthirige, V. D., Pyo, K., Lee, D. & Ramakrishna, G. Energy gap law for exciton dynamics in gold cluster molecules. *J. Phys. Chem. Lett.* **8**, 4898–4905 (2017). 28933858 10.1021/acs.jpclett.7b01892

[5] Liu, G., Chen, W., Xiong, Z., Wang, Y., Zhang, S. & Xia, Z. Laser-driven broadband near-infrared light source with watt-level output. *Nat. Photonics* **18**, 562–568 (2024).

[6] Zhou, X. et al. Recent advances in cation-engineered A_3_BX_6_ metal halide perovskite for enhanced radiative transition. *Research* **9**, 1281 (2026). 42158316 10.34133/research.1281 PMC13181171

[7] Dai, M., Zhou, B. & Yan, D. Rare earth single-atomic hybrid glasses for near-infrared II optical waveguides. *Angew. Chem. Int. Ed.* **64**, e202505322 (2025). 10.1002/anie.202505322 40263969

[8] Jia, Z. et al. Strategies to approach high performance in Cr^3+^-doped phosphors for high-power NIR-LED light sources. *Light Sci. Appl.* **9**, 86 (2020). 32435469 10.1038/s41377-020-0326-8 PMC7229223

[9] Lin, Q., Wu, X., Peng, J., You, W., Ye, X. & Huang, D. Near-unity internal quantum efficiency and high thermal stability of Sr_3_MgGe_5_O_14:_ Cr^3+^ phosphor for plant growth. *Laser Photonics Rev.* **19**, 2402047 (2025).

[10] Rajendran, V. et al. Chromium ion pair luminescence: a strategy in broadband near-infrared light-emitting diode design. *J. Am. Chem. Soc.* **143**, 19058–19066 (2021). 34735772 10.1021/jacs.1c08334

[11] Wu, Q. et al. Ce^3+^,Cr^3+^ co-doped garnet phosphors with yellow and near-infrared emission for white and near-IR dual-mode pc-LEDs. *Sci. China Mater.* **67**, 3932–3940 (2024).

[12] Xiao, W., Basore, E. T., Zheng, G., Liu, X., Xu, B. & Qiu, J. Suppressed concentration quenching brightens short-wave infrared emitters. *Adv. Mater.* **35**, 2306517 (2023). 10.1002/adma.202306517 37643539

[13] Shi, W. et al. Near-unity NIR phosphorescent quantum yield from a room-temperature solvated metal nanocluster. *Science* **383**, 326–330 (2024). 38236955 10.1126/science.adk6628

[14] Ma, H. et al. Bioactive NIR-II gold clusters for three-dimensional imaging and acute inflammation inhibition. *Sci. Adv.* **9**, eadh7828 (2023). 37531420 10.1126/sciadv.adh7828 PMC10396295

[15] Li, B. et al. Efficient and stable near-infrared InAs quantum dot light-emitting diodes. *Nat. Commun.* **16**, 2450 (2025). 40069203 10.1038/s41467-025-57746-1 PMC11897344

[16] Mikhaylov, M. A. & Sokolov, M. N. Molybdenum iodides-from obscurity to bright luminescence. *Eur. J. Inorg. Chem.* **2019**, 4181–4197 (2019).

[17] Kirakci, K., Shestopalov, M. A. & Lang, K. Recent developments on luminescent octahedral transition metal cluster complexes towards biological applications. *Coord. Chem. Rev.* **481**, 215048 (2023).

[18] Sokolov, M. N., Mihailov, M. A., Peresypkina, E. V., Brylev, K. A., Kitamura, N. & Fedin, V. P. Highly luminescent complexes [Mo_6_X_8_(n-C_3_F_7_COO)_6_]^2-^ (X = Br, I). *Dalton Trans.* **40**, 6375–6377 (2011). 21594270 10.1039/c1dt10376h

[19] Evtushok, D. V. et al. A comparative study of optical properties and X-ray induced luminescence of octahedral molybdenum and tungsten cluster complexes. *Dalton Trans.* **46**, 11738–11747 (2017). 28828417 10.1039/c7dt01919j

[20] Efremova, O. A. et al. Octahedral molybdenum cluster complexes with aromatic sulfonate ligands. *Dalton Trans.* **45**, 15427–15435 (2016). 27605435 10.1039/c6dt02863b

[21] Kirakci, K., Sícha, V., Holub, J., Kubát, P. & Lang, K. Luminescent hydrogel particles prepared by self-assembly of β-cyclodextrin polymer and octahedral molybdenum cluster complexes. *Inorg. Chem.* **53**, 13012–13018 (2014). 25419981 10.1021/ic502144z

[22] Kirakci, K. et al. A comparative study of the redox and excited state properties of (n-Bu_4_N)_2_[Mo_6_X_14_] and (n-Bu_4_N)_2_[Mo_6_X_8_(CF_3_COO)_6_] (X = Cl, Br, or I). *Dalton Trans.* **42**, 7224–7232 (2013). 23532319 10.1039/c3dt32863e

[23] Vorotnikova, N. A., Vorotnikov, Y. A. & Shestopalov, M. A. Functional materials containing luminescent octahedral halide clusters of molybdenum and tungsten: diversity of synthetic approaches and binding modes. *Coord. Chem. Rev.* **500**, 215543 (2024).

[24] Li, B. et al. In situ recrystallization of zero-dimensional hybrid halide glass-ceramics toward improved scintillation performance. *Chem. Sci.* **14**, 12238–12245 (2023). 37969591 10.1039/d3sc04332k PMC10631250

[25] Nie, F. & Yan, D. Molecular glass from solution self-assembly. *Acc. Chem. Res.* **58**, 3010–3020 (2025). 40939047 10.1021/acs.accounts.5c00425

[26] Chen, T. & Yan, D. Radical-induced hour-level afterglow and efficient circularly polarized luminescence from metal halide hybrid glasses. *Angew. Chem. Int. Ed.* **138**, e8889513 (2026). 10.1002/anie.8889513 41841233

[27] Li, B., Wang, Y., Xu, Y. & Xia, Z. Emerging 0D hybrid metal halide luminescent glasses. *Adv. Mater.* **37**, 2415483 (2025). 10.1002/adma.202415483 39744778

[28] Nie, F., Wang, K.-Z. & Yan, D. Supramolecular glasses with color-tunable circularly polarized afterglow through evaporation-induced self-assembly of chiral metal–organic complexes. *Nat. Commun.* **14**, 1654 (2023). 36964159 10.1038/s41467-023-37331-0 PMC10039082

[29] He, X., Zhang, S., Wang, Y., Han, K. & Xia, Z. Halogen engineering in copper(I) halide glass scintillators for improved light yield and X-ray imaging resolution. *Adv. Funct. Mater.* **36**, e21093 (2026).

[30] Nie, F. & Yan, D. Supramolecular glass: a new platform for ultralong phosphorescence. *Sci. China Mater.* **67**, 3531–3536 (2024).

[31] Su, B., Geng, S., Xiao, Z. & Xia, Z. Highly distorted antimony(III) chloride [Sb_2_Cl_8_]^2-^ dimers for near-infrared luminescence up to 1070 nm. *Angew. Chem. Int. Ed.* **134**, e202208881 (2022). 10.1002/anie.202208881 35737598

[32] Li, B. et al. Hybrid Cu(I)-based glassy cluster gel scintillator film by in situ UV photopolymerization. *Matter* **8**, 102214 (2025).

[33] Zhou, B. & Yan, D. Glassy inorganic-organic hybrid materials for photonic applications. *Matter* **7**, 1950–1976 (2024).

[34] Cai, P. et al. Highly efficient near-infrared emission (IQE 85%/EQE 75%) from [(Mo_6_I_8_)I_6_]^2-^ cluster-doped (ETP)_2_SbCl_5_ 0D halide glass for multifunctional optoelectronic applications. *Laser*. *Photonics Rev.* **20**, e01665 (2026).

[35] Barnard, P. A., Sun, I.-W. & Hussey, C. L. Molybdenum(II) chloride in the aluminum chloride-1-methyl-3-ethylimidazolium chloride molten salt: electrochemical and spectroscopic characterization of the [(Mo_6_Cl_8_)Cl_6_]^2-^ ion in neutral and basic melts. *Inorg. Chem.* **29**, 3670–3674 (1990).

[36] Ramirez-Tagle, R. & Arratia-Pérez, R. Electronic structure and molecular properties of the [Mo_6_X_8_L_6_]^2-^; X = Cl, Br, I; L = F, Cl, Br, I clusters. *Chem. Phys. Lett.* **460**, 438–441 (2008).

[37] Xu, T. et al. Regulated room temperature phosphorescence from zero-dimensional organometallic halide hybrids for anti-counterfeiting and encryption. *J. Lumin.* **248**, 118979 (2022).

[38] Kirakci, K., Zelenka, J., Krizova, I., Ruml, T. & Lang, K. Octahedral molybdenum cluster complexes with optimized properties for photodynamic applications. *Inorg. Chem.* **59**, 9287–9293 (2020). 32516524 10.1021/acs.inorgchem.0c01173

[39] Liu, X. et al. Mn^4+^-doped Mg_2_ScSbO_6_ deep-red-emitting double-perovskite phosphors for plant cultivation. *J. Lumin.* **256**, 119624 (2023).

[40] Williams, F. E. & Eyring, H. The mechanism of the luminescence of solids. *J. Chem. Phys.* **15**, 289–304 (1947).

[41] Azumi, T. & Saito, Y. Electronic structures of the lower triplet sublevels of hexanuclear molybdenum(II) chloride cluster. *J. Phys. Chem.* **92**, 1715–1721 (1988).

[42] Kitamura, N. et al. Excited triplet states of [{Mo_6_Cl_8_}Cl_6_]^2-^, [{Re_6_S_8_}Cl_6_]^4-^, and [{W_6_Cl_8_}Cl_6_]^2-^ clusters. *Bull. Chem. Soc. Jpn.* **90**, 1164–1173 (2017).

[43] Akagi, S., Fujii, S. & Kitamura, N. Zero-magnetic-field splitting in the excited triplet states of octahedral hexanuclear molybdenum(II) clusters: [Mo_6_X_8_Y_6_]^2-^ (X, Y = Cl, Br, I). *J. Phys. Chem. A* **122**, 9014–9024 (2018). 30365893 10.1021/acs.jpca.8b09339

[44] Hu, F., Long, Z. C., Zhao, F., Shi, W. Q., Zhou, M. & Wang, Q. M. Anti-heavy-atom effect observed in near-infrared emissive bimetallic nanoclusters Au_28_Cu_12_X_4_Cl_4_ (X = Cl, Br, and I). *J. Am. Chem. Soc.* **147**, 19886–19892 (2025). 40423994 10.1021/jacs.5c04083

[45] Brückner, P., Preetz, W. & Pünjer, M. Darstellung, Kristallstrukturen, NMR-, Schwingungsspektren und Normalkoordinatenanalyse der Clusteranionen [(Mo_6_I_8_)Y_6_]^2-^, Y = F, Cl, Br, I. *Z. Anorg. Allg. Chem.* **623**, 8–17 (1997).

[46] Mikhailov, M. A. et al. Synthetic tuning of redox, spectroscopic, and photophysical properties of {Mo_6_I_8_}^4+^ core cluster complexes by terminal carboxylate ligands. *Inorg. Chem.* **55**, 8437–8445 (2016). 27505303 10.1021/acs.inorgchem.6b01042

[47] Nie, F. & Yan, D. Zero-dimensional halide hybrid bulk glass exhibiting reversible photochromic ultralong phosphorescence. *Nat. Commun.* **15**, 5519 (2024). 38951508 10.1038/s41467-024-49886-7 PMC11217438

[48] Jiang, C., Yan, J., Qiu, J., Wu, M. & Xu, B. Regulating intermolecular interactions for stable multifunctional organic–inorganic metal halide hybrid glasses. *Mater. Horiz.* **12**, 3515–3524 (2025). 39992681 10.1039/d4mh01427h

[49] Ali, M. A., Liu, X., Sun, H.-T., Ren, J. & Qiu, J. Metal inorganic–organic complex glass and fiber for photonic applications. *Chem. Mater.* **34**, 2476–2483 (2022).

[50] Stern, O. & Volmer, M. Über die Abklingzeit der Fluoreszenz. *Phys. Z.* **20**, 183–188 (1919).

[51] Cai, P. et al. Re^4+^/Te^4+^ co-doped Cs_2_ZrCl_6_ double perovskite microcrystals: broadening excitation range and boosting luminescent performance for near-infrared lighting and non-destructive quality inspection. *Mater. Today Chem.* **48**, 102954 (2025).

[52] Shi, R., Miao, S., Lv, X., Chen, D., Zhang, Y. & Liang, Y. High-efficiency short-wave infrared emitter enabled by Cr^3+^–Yb^3+^ co-doped phosphor. *Adv. Opt. Mater.* **12**, 2303221 (2024).

[53] Zheng, L. et al. Ultrabroadband near-infrared Zn_3_Ga_2_GeO_8_: Cr^3+^,Ni^2+^ phosphor for PiG pc-LED. *Adv. Opt. Mater.* **12**, 2401521 (2024).

[54] Li, B. et al. Enhanced VIS-NIR emission of Re^4+^-doped Cs_2_ZrCl_6_ for optical thermometry and near-infrared illumination applications. *Inorg. Chem. Front.* **11**, 5157–5171 (2024).

[55] Peng, H. et al. Efficient broadband near-infrared emitters in lead-free metal halides with record photoluminescence quantum yield under blue light excitation. *Adv. Funct. Mater.* **34**, 2411807 (2024).

[56] Kühne, T. D. et al. CP2K: an electronic structure and molecular dynamics software package-Quickstep: efficient and accurate electronic structure calculations. *J. Chem. Phys* **152**, 194103 (2020). 33687235 10.1063/5.0007045

[57] Perdew, J. P., Burke, K. & Ernzerhof, M. Generalized gradient approximation made simple. *Phys. Rev. Lett.* **77**, 3865–3868 (1996). 10062328 10.1103/PhysRevLett.77.3865

[58] Grimme, S., Ehrlich, S. & Goerigk, L. Effect of the damping function in dispersion-corrected density functional theory. *J. Comput. Chem.* **32**, 1456–1465 (2011). 21370243 10.1002/jcc.21759

[59] Goedecker, S., Teter, M. & Hutter, J. Separable dual-space Gaussian pseudopotentials. *Phys. Rev. B* **54**, 1703–1710 (1996). 10.1103/physrevb.54.1703 9986014

[60] Hartwigsen, C., Goedecker, S. & Hutter, J. Relativistic separable dual-space Gaussian pseudopotentials from H to Rn. *Phys. Rev. B* **58**, 3641–3662 (1998). 10.1103/physrevb.54.1703 9986014

[61] Frisch, M. J. et al. *Gaussian 09, Revision A.02* (Gaussian, Inc., Wallingford CT, 2009).

[62] Scalmani, G. et al. Geometries and properties of excited states in the gas phase and in solution: theory and application of a time-dependent density functional theory polarizable continuum model. *J. Chem. Phys.* **124**, 094107 (2006). 10.1063/1.2173258 16526845

[63] Adamo, C. & Barone, V. Toward reliable density functional methods without adjustable parameters: the PBE0 model. *J. Chem. Phys.* **110**, 6158–6170 (1999).

[64] Marenich, A. V., Cramer, C. J. & Truhlar, D. G. Universal solvation model based on solute electron density and on a continuum model of the solvent defined by the bulk dielectric constant and atomic surface tensions. *J. Phys. Chem. B* **113**, 6378–6396 (2009). 19366259 10.1021/jp810292n

[65] Hay, P. J. & Wadt, W. R. Ab initio effective core potentials for molecular calculations. Potentials for K to Au including the outermost core orbitals. *J. Chem. Phys.* **82**, 299–310 (1985).

[66] Wadt, W. R. & Hay, P. J. Ab initio effective core potentials for molecular calculations. Potentials for main group elements Na to Bi. *J. Chem. Phys.* **82**, 284–298 (1985).

[67] Hay, P. J. & Wadt, W. R. Ab initio effective core potentials for molecular calculations. Potentials for the transition metal atoms Sc to Hg. *J. Chem. Phys.* **82**, 270–283 (1985).

[68] Check, C. E., Faust, T. O., Bailey, J. M., Wright, B. J., Gilbert, T. M. & Sunderlin, L. S. Addition of polarization and diffuse functions to the LANL2DZ basis set for p-block elements. *J. Phys. Chem. A* **105**, 8111–8116 (2001).

[69] Hariharan, P. C. & Pople, J. A. Influence of polarization functions on molecular-orbital hydrogenation energies. *Theor. Chim. Acta* **28**, 213–222 (1973).

