# Supplementary material for: Near-unity near-infrared emission from Mo6 cluster doped hybrid halide glasses via halide-ligand and matrix engineering
Source: Nat Commun. 2026 Jul 22;17:8947. doi: 10.1038/s41467-026-75934-5 (PMC13503768; doi:10.1038/s41467-026-75934-5)
Supplement: Supplementary file 1 [file 41467_2026_75934_MOESM1_ESM.pdf]

Supplementary Information

Near-Unity Near-Infrared Emission from Mo_6 Cluster doped Hybrid Halide Glasses via Halide-Ligand and Matrix Engineering

Xuhui Feng (冯旭晖)[1†], Peiqing Cai (蔡培庆)[1†]*, Da Lei (雷达)[2,3], Keyuan Liu (刘克原)[1], Haohan Wang (王浩涵)[4], Hang Yin (尹航)[4]*, Qinhua Wei (魏钦华)[4], Zutao Fan (范祖涛)[5], Zhaoyang Chen (陈朝阳)[6,7], Shala Bi (比沙拉)[6,7]*, Zugang Liu (刘祖刚)[1]*

[1]College of Optical and Electronic Technology, China Jiliang University, Hangzhou, Zhejiang 310018, China.

[2]Key Laboratory of Green and High-end Utilization of Salt Lake Resources, Qinghai Institute of Salt Lakes, Chinese Academy of Sciences, Xining, Qinghai 810008, China

[3]Qinghai Provincial Key Laboratory of Resources and Chemistry of Salt Lakes, Qinghai Institute of Salt Lakes, Chinese Academy of Sciences, Xining, Qinghai 810008, China

[4]College of Materials and Chemistry, China Jiliang University, Hangzhou, Zhejiang 310018, China.

[5]International Collaborative Laboratory of 2D Materials for Optoelectronics Science and Technology of Ministry of Education, Institute of Microscale Optoelectronics, Shenzhen University, Shenzhen, Guangdong 518060, China

[6]State Key Laboratory of Functional Materials and Devices for Special Environments Conditions, Xinjiang Technical Institute of Physics and Chemistry of CAS, Urumqi, Xinjiang 830011, China

[7]Xinjiang Key Laboratory of Electronic Information Materials and Devices, Xinjiang Technical Institute of Physics and Chemistry of CAS, Urumqi, Xinjiang 830011, China

*Correspondence and requests for materials should be addressed to Peiqing Cai (pqcai@cjlu.edu.cn), Hang Yin (yinhang@cjlu.edu.cn), Shala Bi (bsl@ms.xjb.ac.cn) or Zugang Liu (zgliu78@cjlu.edu.cn).

†These authors contributed equally to this work.

Supplementary Table 1 | The crystal structure parameters of $(ETP)_2[\{Mo_6I_8\}X_6]$ (X = Cl, Br, I) single crystals. ()

Structure	$(ETP)_2[\{Mo_6I_8\}Cl_6]$	$(ETP)_2[\{Mo_6I_8\}Br_6]$	$(ETP)_2[\{Mo_6I_8\}I_6]$
CCDC code	2477364	2477363	2425267
Empirical formula	$C_{40}H_{40}Cl_6I_8Mo_6P_2$	$C_{40}H_{40}Br_6I_8Mo_6P_2$	$C_{40}H_{40}I_{14}Mo_6P_2$
Formula weight	2386.20	2652.96	2934.90
Temperature (K)	293.00	293.00	293.00
Crystal system	monoclinic	monoclinic	monoclinic
Space group	C2/c	$P2_1/n$	$P2_1/n$
Unit cell dimensions	a = 27.869(3) Å<br>α = 90°	a = 9.8610(6) Å<br>α = 90°	a = 10.2309(7) Å<br>α = 90°
	b = 13.5829(13) Å<br>β = 126.701(2)°	b = 23.1140(15) Å<br>β = 94.058(2)°	b = 23.2103(18) Å<br>β = 93.711(2)°
	c = 18.871(3) Å<br>γ = 90°	c = 12.9754(7) Å<br>γ = 90°	c = 13.2272(9) Å<br>γ = 90°
Volume ($Å^3$)	5727.3(12)	2950.0(3)	3134.4(4)
Z	4	2	2
Dcalcd (g/cm^3)	2.767	2.987	3.110
μ (mm^{-1})	5.951	9.569	8.137
F(000)	4352.0	2392.0	2608.0
Crystal size (mm^3)	0.13 × 0.12 × 0.09	0.13 × 0.12 × 0.09	0.13 × 0.12 × 0.09
Radiation	MoKα (λ = 0.71073)	MoKα (λ = 0.71073)	MoKα (λ = 0.71073)
Index ranges	$-36 \leq h \leq 36$<br>$-17 \leq k \leq 14$<br>$-23 \leq l \leq 24$	$-12 \leq h \leq 12$<br>$-30 \leq k \leq 24$<br>$-16 \leq l \leq 16$	$-13 \leq h \leq 12$<br>$-30 \leq k \leq 30$<br>$-17 \leq l \leq 16$
Reflections collected	25712	25771	29091
Independent reflections	6515	6776	7204
Goodness-of-fit on F^2	1.104	1.027	1.093
Final R indexes [$I>2\sigma(I)$]	R_1 = 0.0326<br>wR_2 = 0.0665	R_1 = 0.0326<br>wR_2 = 0.0665	R_1 = 0.0416<br>wR_2 = 0.0807
Final R indexes (all data)	R_1 = 0.0460<br>wR_2 = 0.0725	R_1 = 0.0460<br>wR_2 = 0.0725	R_1 = 0.0585<br>wR_2 = 0.0884

(Note: The Cl- and Br-terminated structures were newly determined in this study and deposited

under CCDC 2477364 and 2477363, respectively. The I-terminated structure was previously reported and deposited under CCDC 2425267; it is included here only for structural comparison.)

Supplementary Table 2 | EDS elemental compositions of $(ETP)_2[\{Mo_6I_8\}Cl_6]$, $(ETP)_2[\{Mo_6I_8\}Br_6]$ and $(ETP)_2[\{Mo_6I_8\}I_6]$. The relatively large deviation in the measured C content arises from the limited quantification accuracy of EDS for light elements.

Sample	Element	Weight (%)	Atomic (%)
$(ETP)_2[\{Mo_6I_8\}Cl_6]$	C	34.2	78.7
	P	2.2	2
	Mo	19.0	5.5
	I	37.3	8.1
	Cl	7.3	5.7
$(ETP)_2[\{Mo_6I_8\}Br_6]$	C	28.4	76.0
	P	2.4	2.5
	Mo	18.8	6.3
	I	33.9	8.6
	Br	16.5	6.6
$(ETP)_2[\{Mo_6I_8\}I_6]$	C	24.51	74.93
	P	1.86	2.20
	Mo	16.76	6.41
	I	56.88	16.46

Supplementary Table 3 | Structural diagrams and crystallographic data for individual $[\{Mo_6I_8\}X_6]^{2-}$ (X = Cl, Br, I) clusters with different terminal ligands. All values represent averages.

	Compounds	Mo–Mo (Å)
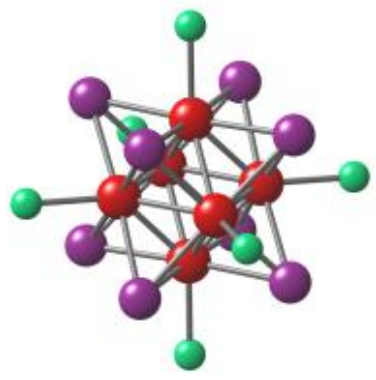	$(ETP)_2[\{Mo_6I_8\}Cl_6]$	2.6675
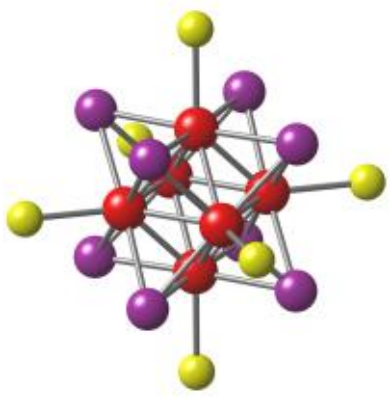	$(ETP)_2[\{Mo_6I_8\}Br_6]$	2.6741
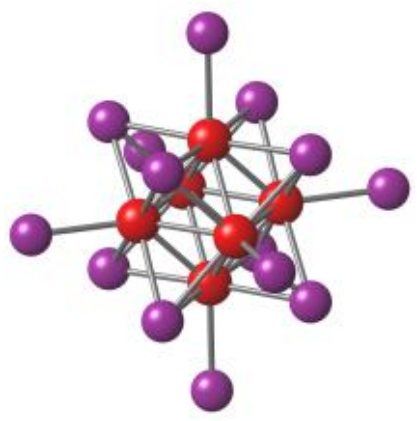	$(ETP)_2[\{Mo_6I_8\}I_6]$	2.6764

Supplementary Table 4 | Photophysical parameters of Y-X glasses.

	Cl-Cl	Cl-Br	Cl-I	Br-Cl	Br-Br	Br-I	I-Cl	I-Br	I-I
λ_{ex} (nm)		720			728			761	
FWHM (nm)		128			170			260	
τ (μs)	212.60	210.23	209.71	166.43	164.65	163.33	85.79	78.53	75.07
IQE (%)	96.95	95.33	90.66	55.16	54.46	45.37	18.64	16.59	14.25

Supplementary Table 5 | Comparison of the photophysical parameters between $(ETP)_2[\{Mo_6I_8\}X_6]$ powders and glasses containing the corresponding dispersed $[\{Mo_6I_8\}X_6]^{2-}$ clusters.

	$(ETP)_2[\{Mo_6I_8\}Cl_6]$	Cl-Cl	$(ETP)_2[\{Mo_6I_8\}Br_6]$	Br-Br	$(ETP)_2[\{Mo_6I_8\}I_6]$	I-I
τ (μs)	162.79	212.60	124.34	164.65	60.11	75.07
IQE (%)	62.05	96.95	41.72	54.46	13.59	14.25
AE (%)	91.07	94.31	91.23	94.03	90.82	93.72

Supplementary Table 6 | Thermodynamic parameters of five different glass matrices doped with $(ETP)_2[\{Mo_6I_8\}Cl_6]$ powder.

	$(ETP)_2CdCl_4$: Mo_6	$(MMP)_2ZnCl_4$: Mo_6	$(MMP)_2SbCl_5$: Mo_6	$(Bzmim)_2SbCl_5$: Mo_6	$ZnCl_2(HbIm)_2$: Mo_6
T_m (°C)	195.6	159.5	128.1	137.1	131.8
T_d (°C)	290	179	158	210	181

Supplementary Table 7 | Repeated IQE, AE and EQE measurements of the optimized $(ETP)_2CdCl_4$:Mo_6 glass.

Parameter (%)	1	2	3	4	5	6	7	8	9	10	11	12	13	14	15	16	17	18
**IQE**	96.87	96.71	96.00	98.51	97.45	98.86	97.26	96.59	96.54	97.05	95.51	97.34	95.83	95.14	98.12	96.12	97.21	96.11
**AE**	93.10	93.07	93.11	93.52	93.51	93.46	92.65	92.74	92.70	92.43	92.44	92.47	93.66	93.57	93.45	93.38	93.36	93.44
**EQE**	90.19	90.01	89.39	92.13	91.13	92.39	90.11	89.58	89.49	89.70	88.29	90.01	89.75	89.02	91.69	89.76	90.76	89.81

Supplementary Table 8 | Summary of repeated measurements of IQE, AE and EQE of $(ETP)_2CdCl_4$:Mo_6 glass.

Parameter	Mean ± s.d.	Minimum	Maximum	n
IQE (%)	96.85 ± 1.00	95.14	98.86	18
AE (%)	93.11 ± 0.43	92.43	93.66	18
EQE (%)	90.18 ± 1.07	88.29	92.39	18

Supplementary Table 9 | Photophysical parameters of compounds based on the $\{Mo_6I_8\}^{4+}$ core at room temperature.

Sample	λ_{em} (nm)	FWHM (cm^{-1})	IQE (%)	Ref.
$Kat_2[\{Mo_6I_8\}I_6]$	/	/	15	[1]
$(4\text{-}ViBnNMe_3)_2[\{Mo_6I_8\}I_6]$	710	/	1	[2]
$(TBA)_2[\{Mo_6I_8\}(NO_3)_6]$	666	2350	26	[3]
$(TBA)_2[\{Mo_6I_8\}(N_3)_6]$	716	/	21	[4]
$(TBA)_2[\{Mo_6I_8\}(CH_3COO)_6]$	699	2310	18	[5]
$(TBA)_2[\{Mo_6I_8\}((CH_3)_3CCOO)_6]$	704	2250	16	[5]
$(TBA)_2[\{Mo_6I_8\}(OOCC_2F_5)_6]$	661	2280	32	[5]
$(TBA)_2[\{Mo_6I_8\}(C_4H_3OCOO)_6]$	696	2370	34	[5]
$(TBA)_2[\{Mo_6I_8\}(C_6H_5COO)_6]$	698	2290	30	[5]
$(TBA)_2[\{Mo_6I_8\}(C_{10}H_7COO)_6]$	704	2150	18	[5]
$Cs_2[\{Mo_6I_8\}(C_2F_5COO)_6]$	650	/	35	[6]
$(TBA)_2[\{Mo_6I_8\}(C_3F_7COO)_6]$	659	2170	36	[7]
$(TBA)_2[\{Mo_6I_8\}(OC_6H_4\text{-p-}(NO_2))_6]$	733	2300	3	[8]
$(TBA)_2[\{Mo_6I_8\}(OC_6H_3\text{-2,4-}(NO_2)_2)_6]$	703	2250	5	[8]
$(TBA)_2[\{Mo_6I_8\}(OTS)_6]$	662	2300	44	[9]
$(TBA)_2[\{Mo_6I_8\}(C_6H_5SO_3)_6]$	657	2200	62	[9]
$(TBA)_2[\{Mo_6I_8\}(SC_6F_4H)_6]$	713	3140	4	[10]
$(TBA)_2[\{Mo_6I_8\}(C{\equiv}CC(O)OCH_3)_6]$	700	2600	5	[11]
$[TPEIm_2][Mo_6I_8(C_2F_5COO)_6]$	668	/	31	[12]
$(DAPIm)_2[\{Mo_6I_8\}(C_2F_5COO)_6]$	670	/	12	[13]
$(TBA)_2[\{Mo_6I_8\}Cl_6]$	705	2520	47	[14]
$(TBA)_2[\{Mo_6I_8\}Br_6]$	713	3230	23	[14]
$(TBA)_2[\{Mo_6I_8\}I_6]$	738	4580	11	[14]
$(ETP)_2[\{Mo_6I_8\}Cl_6]$	708	2515	62.05	**This work**
$(ETP)_2[\{Mo_6I_8\}Br_6]$	720	3568	41.72	**This work**
$(ETP)_2[\{Mo_6I_8\}I_6]$	740	4200	13.59	**This work**
$(ETP)_2CdCl_4{:}Mo_6$ glass	720	2488	96.95	**This work**

Supplementary Table 10 | Number of line pairs/mm in USAF resolving power test target 1951.

Group number / Element	0	1	2	3	4	5	6
**1**	1.00	2.00	4.00	8.00	16.00	32.00	64.00
**2**	1.12	2.24	4.49	8.98	17.96	35.90	71.80
**3**	1.26	2.52	5.04	10.08	20.16	40.30	80.60
**4**	1.41	2.83	5.66	11.31	22.63	45.30	90.50
**5**	1.59	3.17	6.35	12.70	25.40	50.80	101.6
**6**	1.78	3.56	7.13	14.25	28.51	57.00	114.0

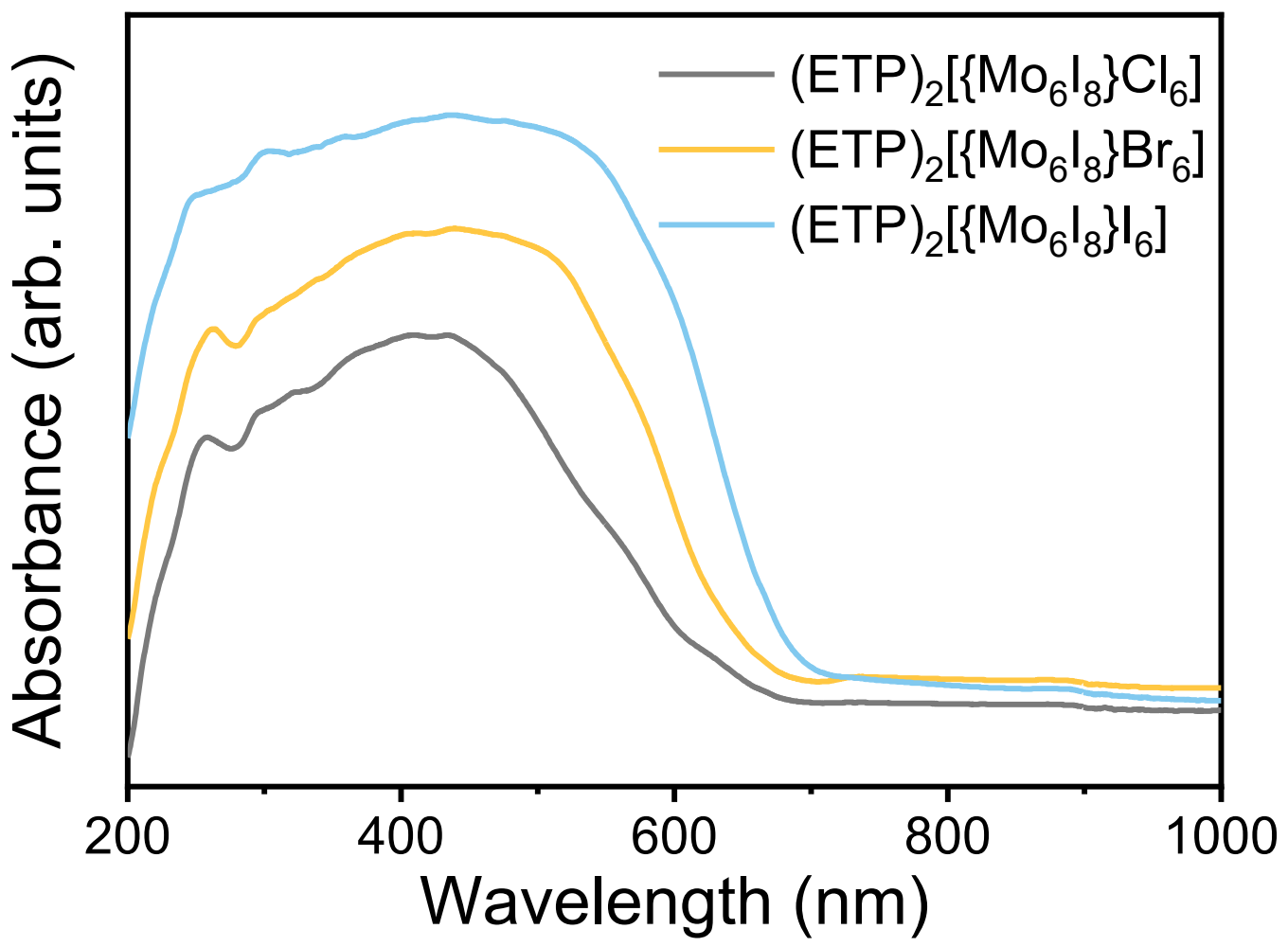

Supplementary Figure 1 | Absorption properties of molybdenum cluster powders. Absorption spectra of $(ETP)_2[\{Mo_6I_8\}X_6]$ powders.

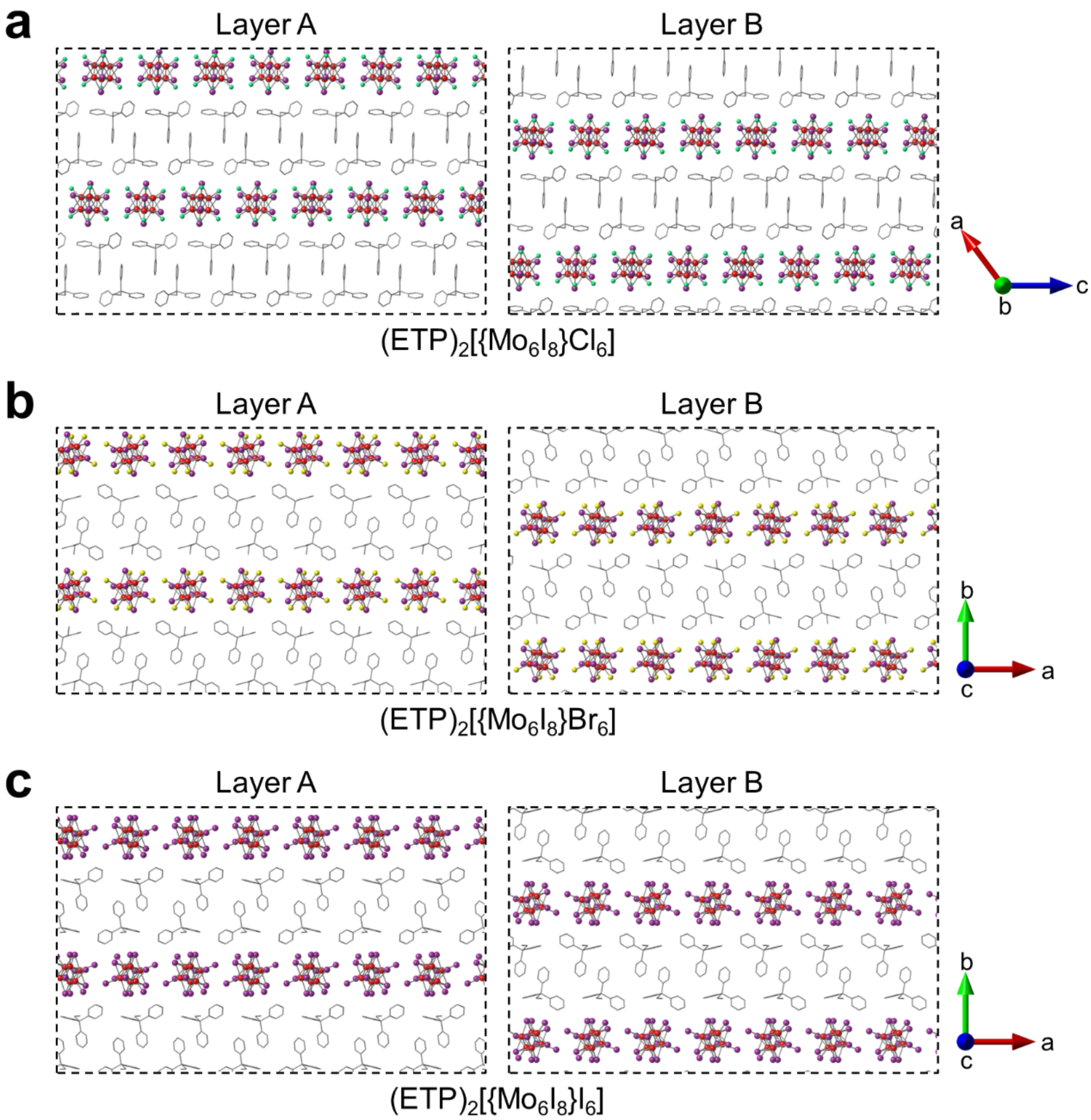

Supplementary Figure 2 | Two-dimensional single-layer crystal structures of molybdenum cluster salts. Two-dimensional single-layer structure of **a**, $(ETP)_2[\{Mo_6I_8\}Cl_6]$, **b,** $(ETP)_2[\{Mo_6I_8\}Br_6]$ and **c,** $(ETP)_2[\{Mo_6I_8\}I_6]$. The three-dimensional crystal structure arises from the spatial stacking of alternating A and B layers.

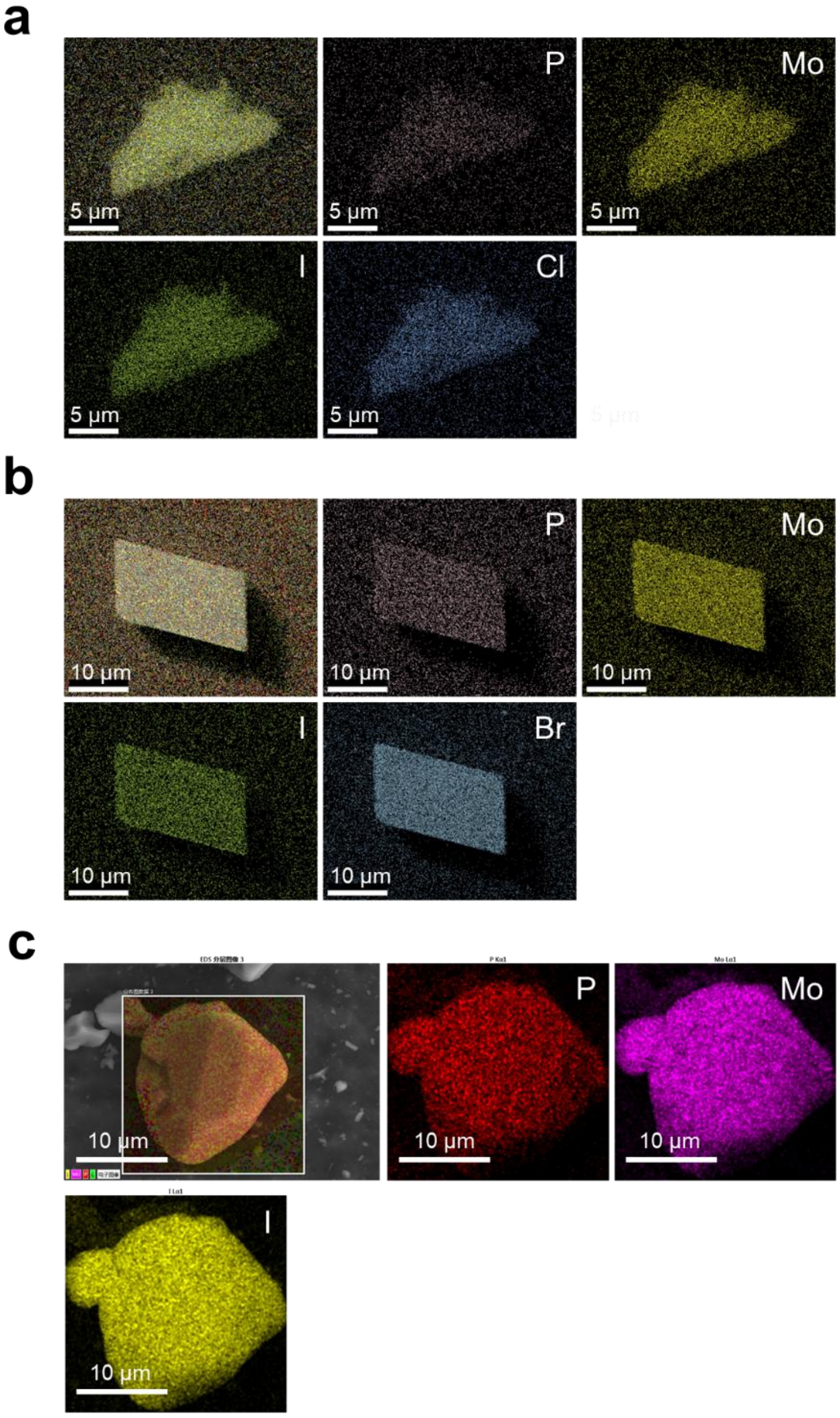

Supplementary Figure 3 | Elemental mapping of molybdenum cluster salts. Elemental mapping results for **a,** $(ETP)_2[\{Mo_6I_8\}Cl_6]$, **b,** $(ETP)_2[\{Mo_6I_8\}Br_6]$ and **c,** $(ETP)_2[\{Mo_6I_8\}I_6]$.

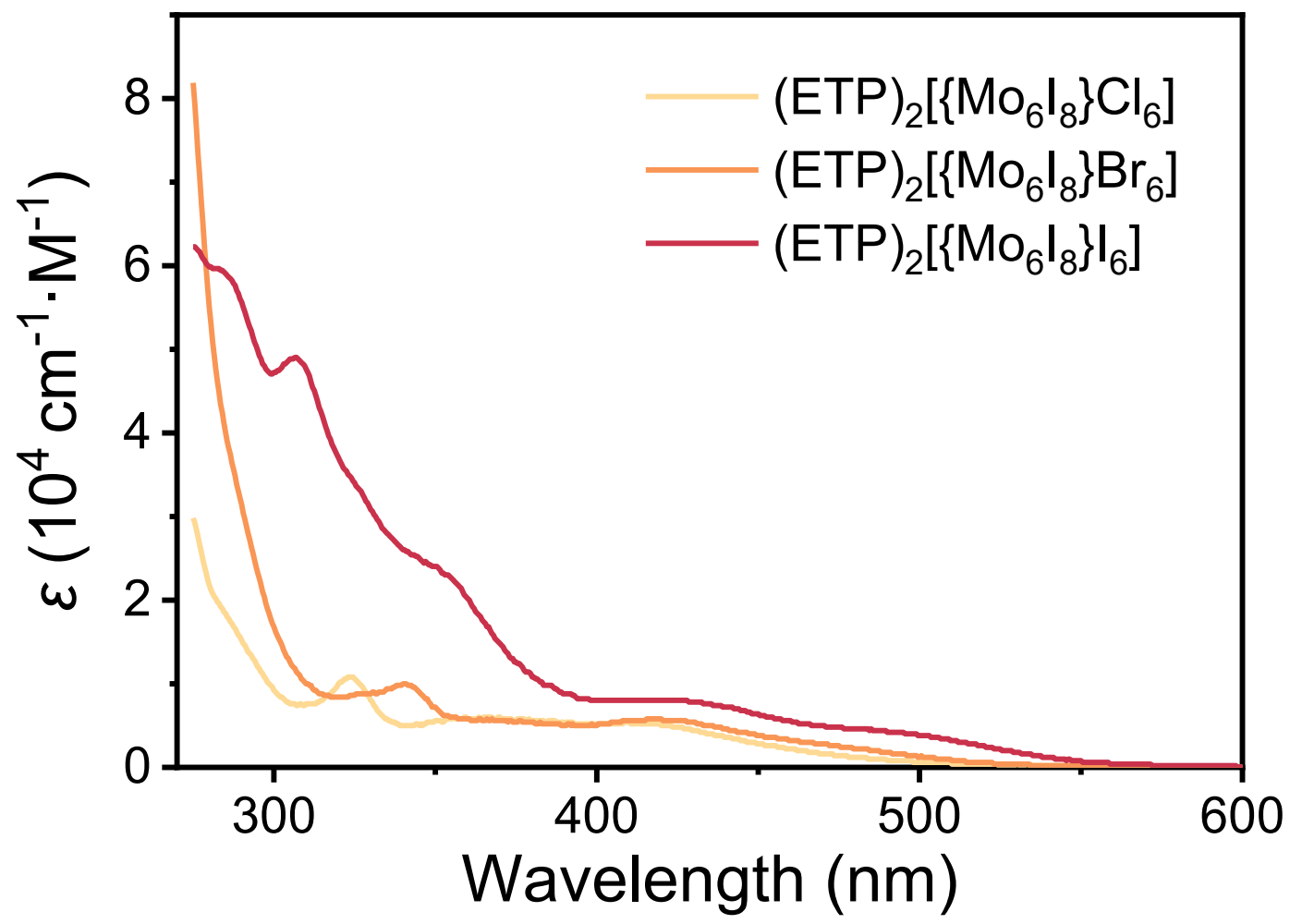

Supplementary Figure 4 | Absorption spectra of molybdenum cluster salts in solution. Ultraviolet–visible absorption spectra of $(ETP)_2[\{Mo_6I_8\}X_6]$ in N,N-dimethylformamide (DMF) at concentrations of 5.0×10^{-6} M. Measurements performed using a 1 cm path length quartz cuvette.

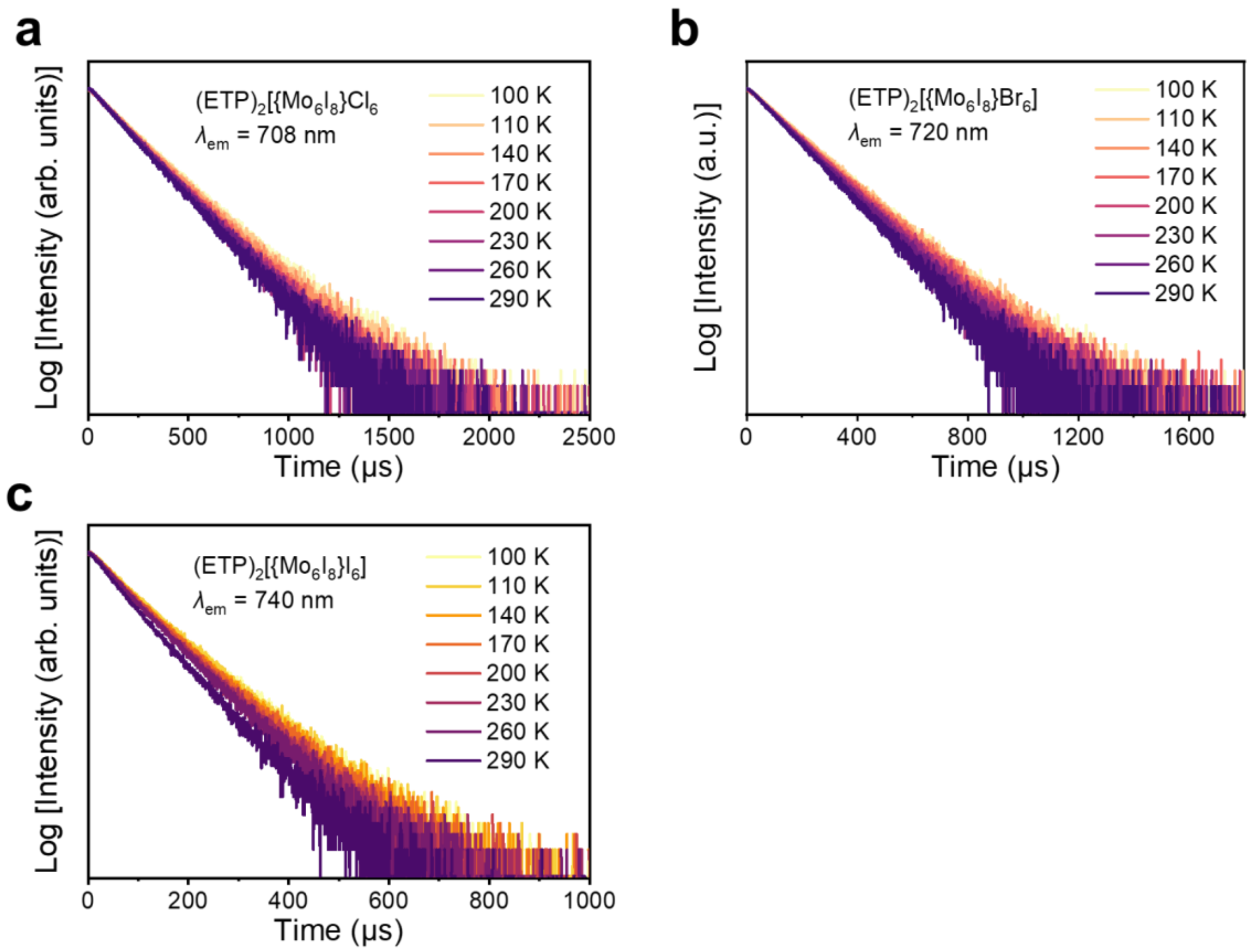

Supplementary Figure 5 | Temperature-dependent decay dynamics of molybdenum cluster salts. Temperature-dependent decay curves of **a,** $(ETP)_2[\{Mo_6I_8\}Cl_6]$, **b,** $(ETP)_2[\{Mo_6I_8\}Br_6]$ and **c,** $(ETP)_2[\{Mo_6I_8\}I_6]$.

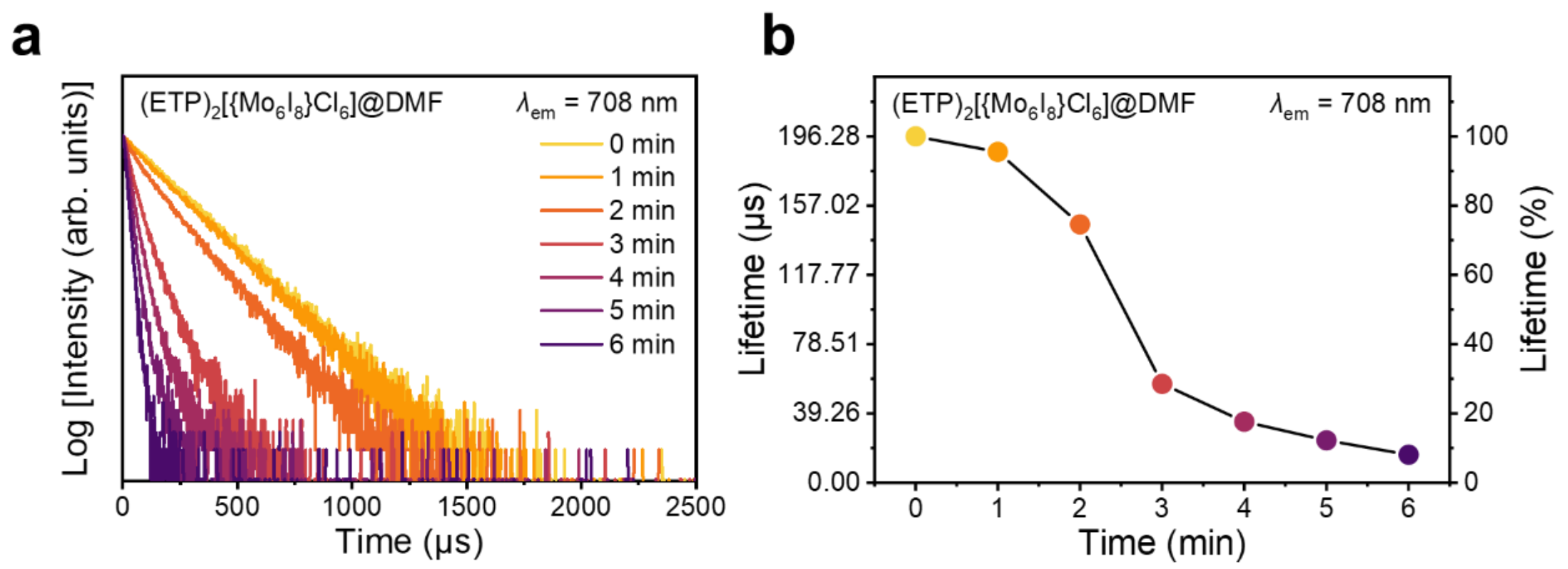

Supplementary Figure 6 | Air-exposure-dependent decay dynamics of deoxygenated cluster solution. a, Decay curves and **b,** corresponding lifetimes of deoxygenated DMF solution of $(ETP)_2[\{Mo_6I_8\}Cl_6]$ as a function of air exposure time.

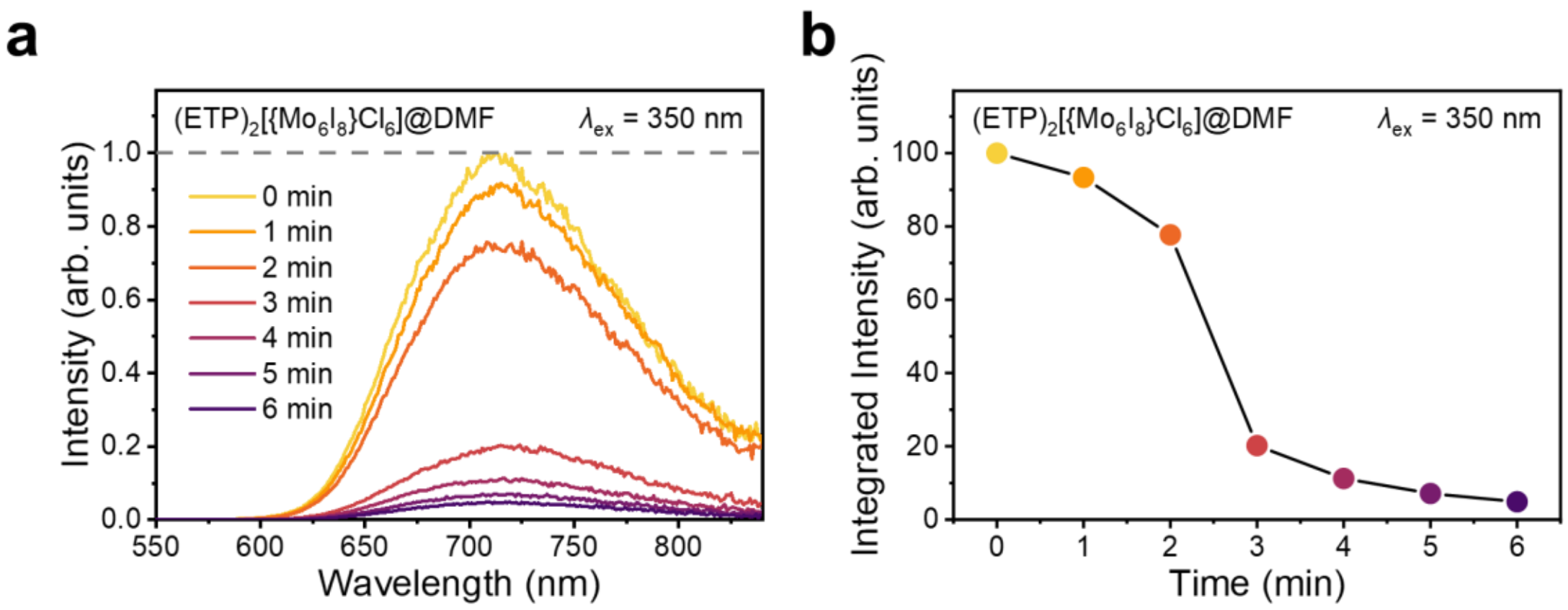

Supplementary Figure 7 | Air-exposure-dependent emission intensity of deoxygenated cluster solution. a, Photoluminescence (PL) spectra and **b,** corresponding integrated intensities of deoxygenated DMF solution of $(ETP)_2[\{Mo_6I_8\}Cl_6]$ as a function of air exposure time.

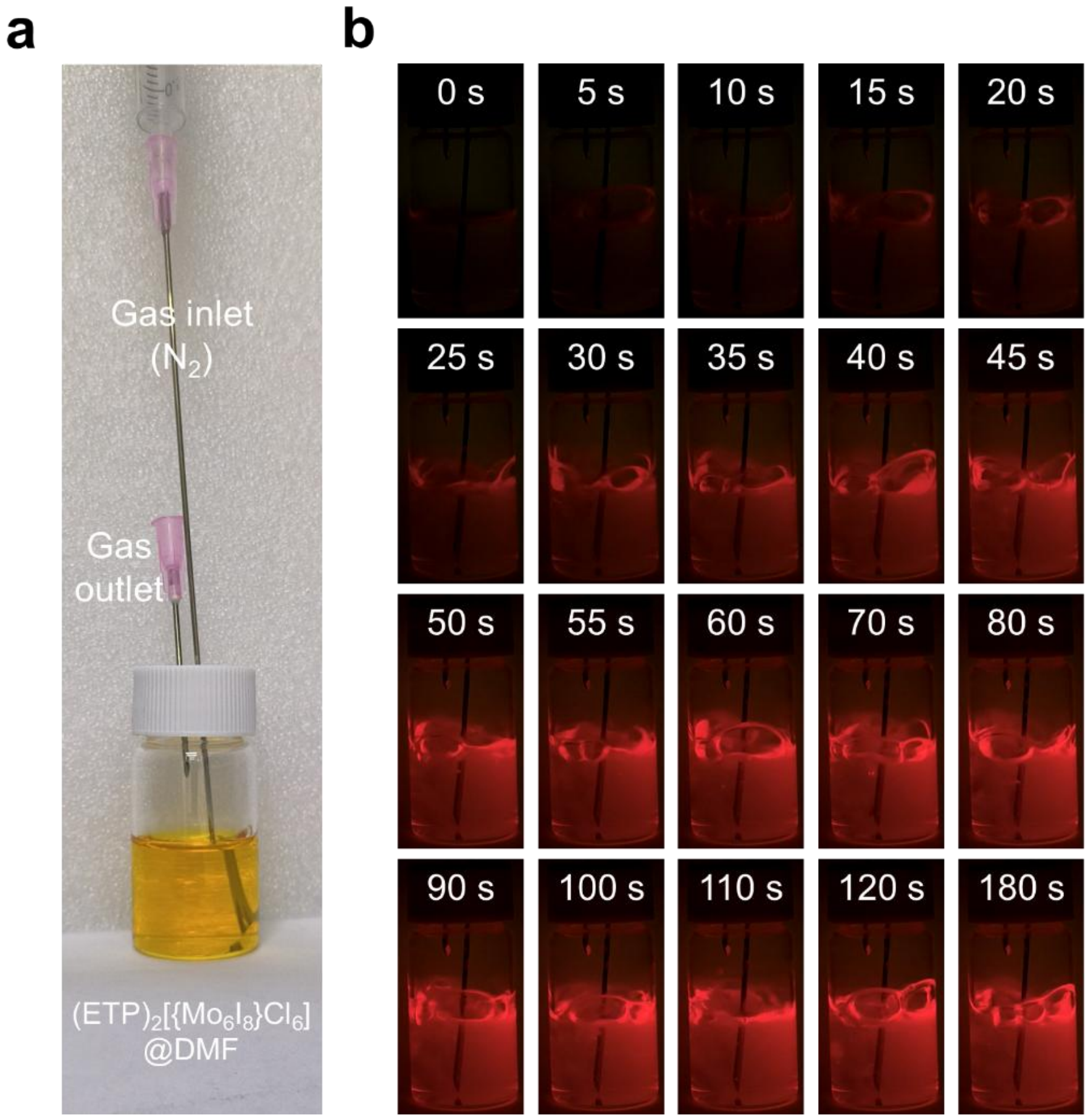

Supplementary Figure 8 | Nitrogen-bubbling treatment of air-exposed cluster solution. a, Schematic illustration of nitrogen bubbling through an air-exposed DMF solution of $(ETP)_2[\{Mo_6I_8\}Cl_6]$ to remove dissolved oxygen. **b,** Photographs of the DMF solution of $(ETP)_2[\{Mo_6I_8\}Cl_6]$ under UV illumination as a function of nitrogen bubbling time.

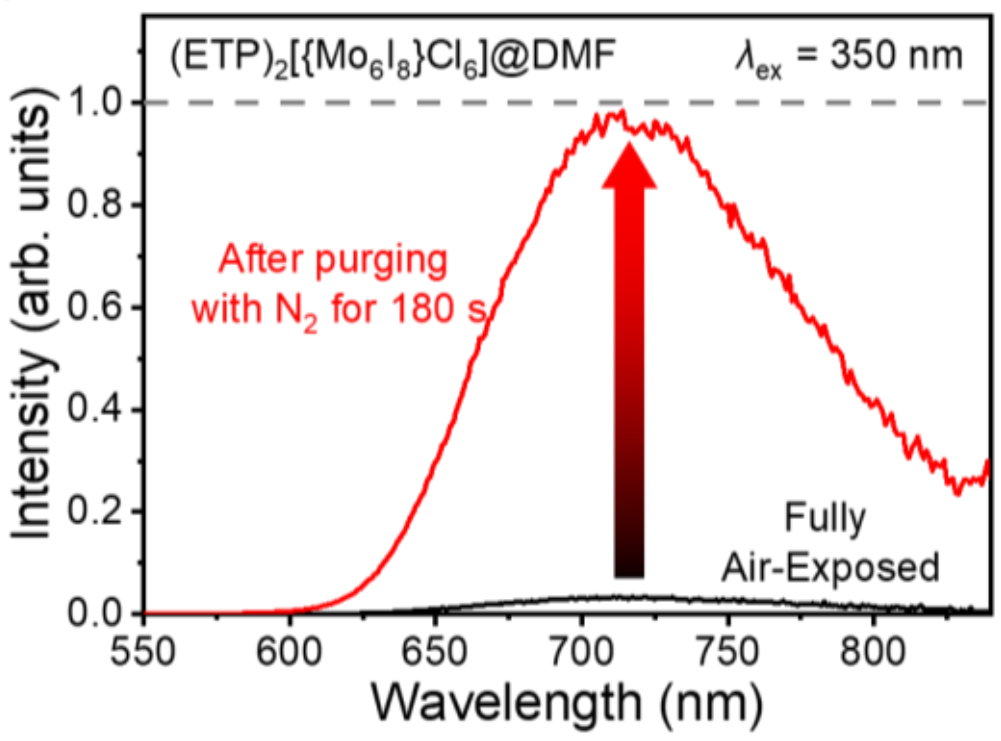

Supplementary Figure 9 | Emission recovery of air-exposed cluster solution after nitrogen bubbling. PL spectra of the DMF solution of $(ETP)_2[\{Mo_6I_8\}Cl_6]$ after full exposure to air and after nitrogen bubbling for 180 s.

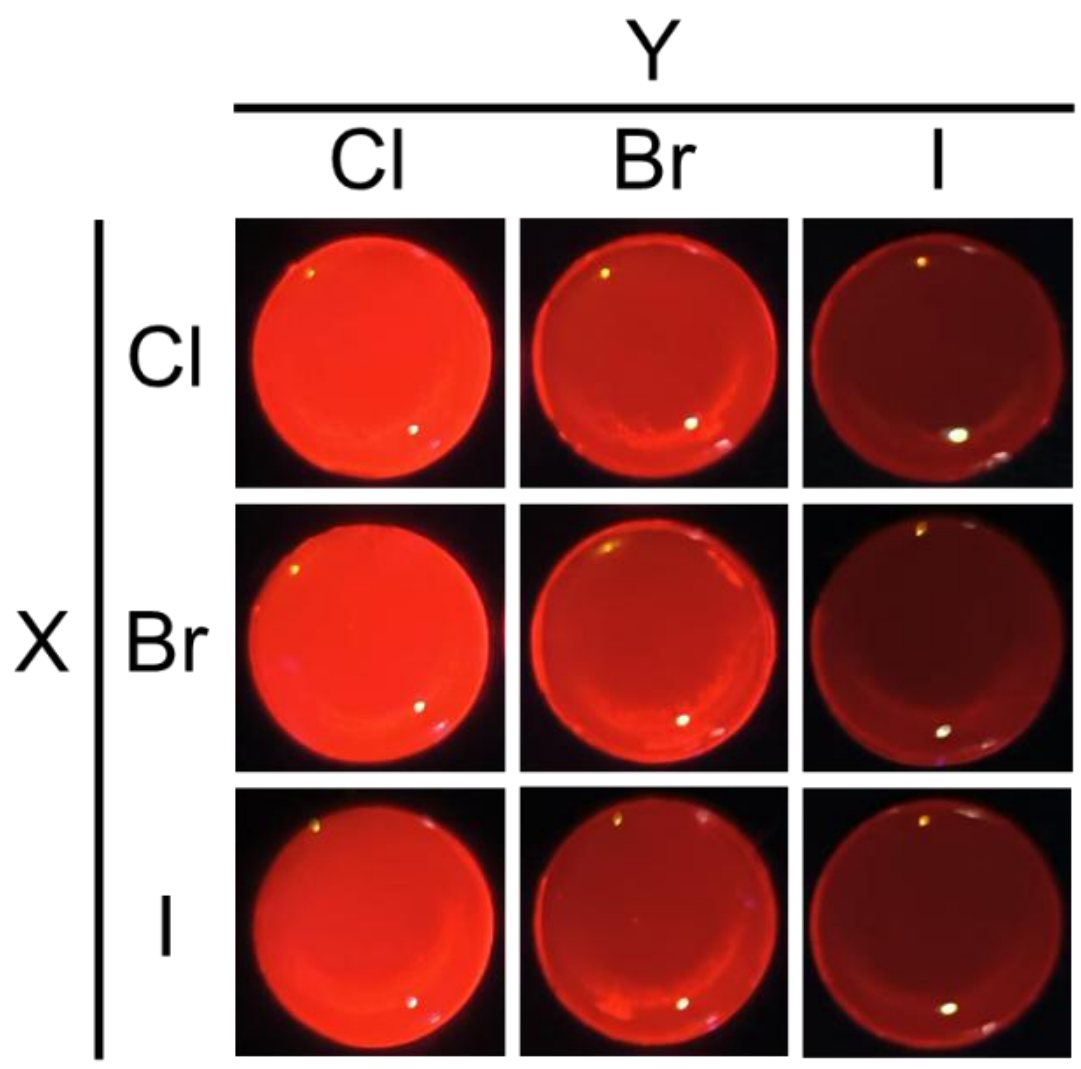

Supplementary Figure 10 | Ultraviolet-excited photographs of Y-X glasses. Photographs of Y-X glasses under ultraviolet illumination captured using a visible-light camera.

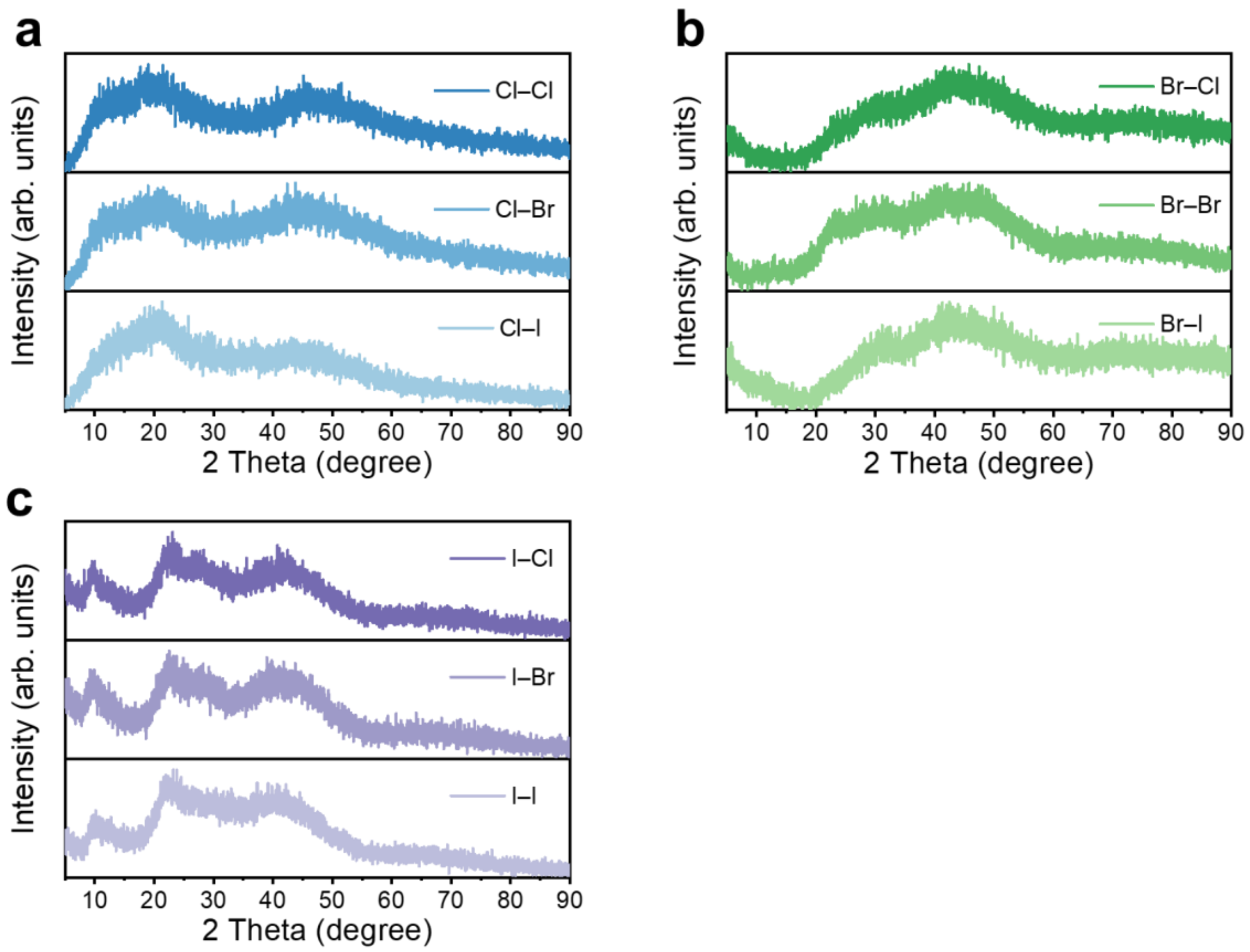

Supplementary Figure 11 | Structural characterization of Y-X glasses. Powder X-ray diffraction patterns of Y-X glasses, including **a,** Cl-Cl, Cl-Br, Cl-I, **b,** Br-Cl, Br-Br, Br-I, **c,** I-Cl, I-Br and I-I glasses.

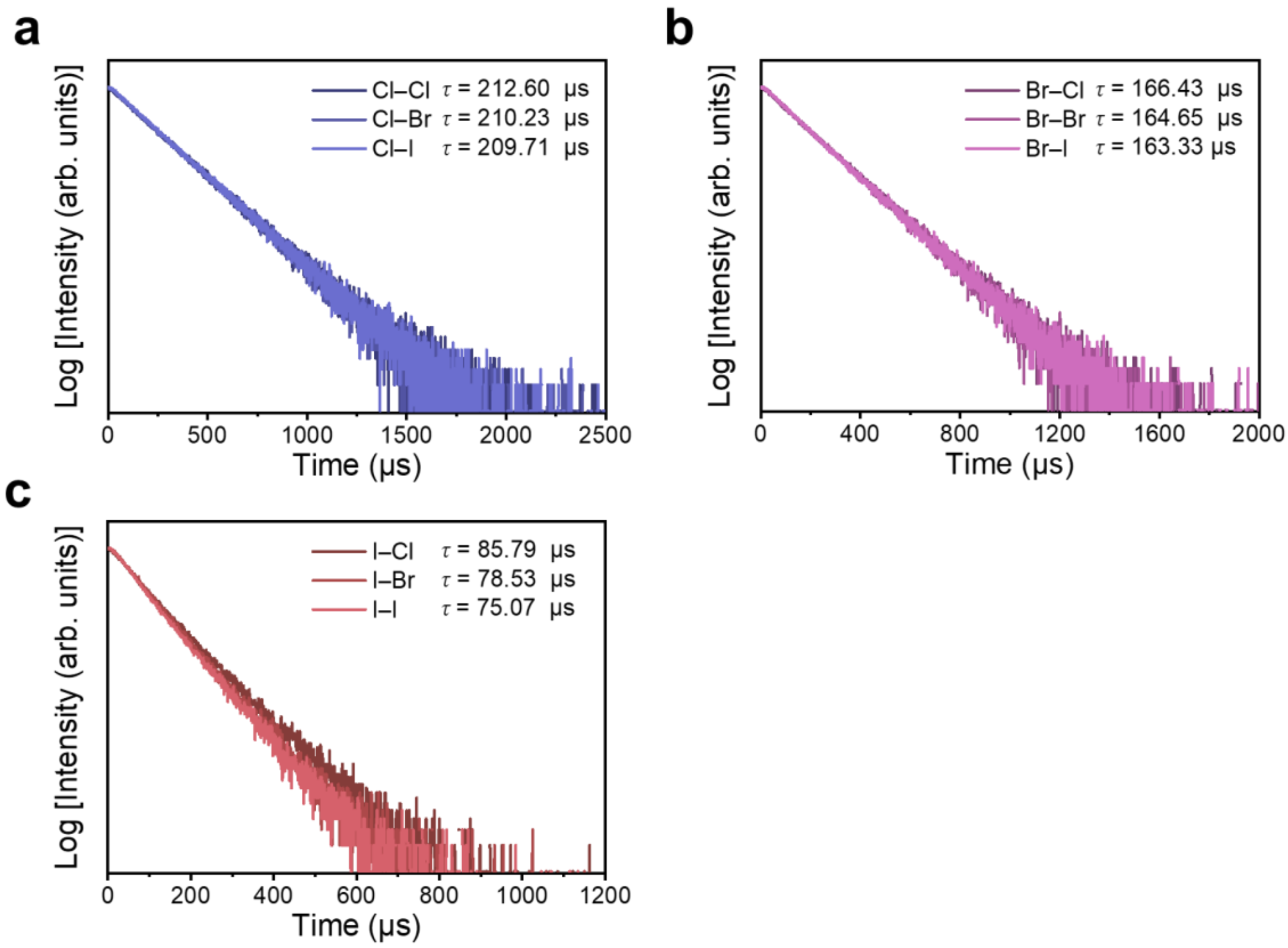

Supplementary Figure 12 | Photoluminescence decay dynamics of Y-X glasses. Photoluminescence decay curves of Y-X glasses, including **a,** Cl-Cl, Cl-Br, Cl-I, **b,** Br-Cl, Br-Br, Br-I, **c,** I-Cl, I-Br and I-I glasses.

Cl-Cl glass

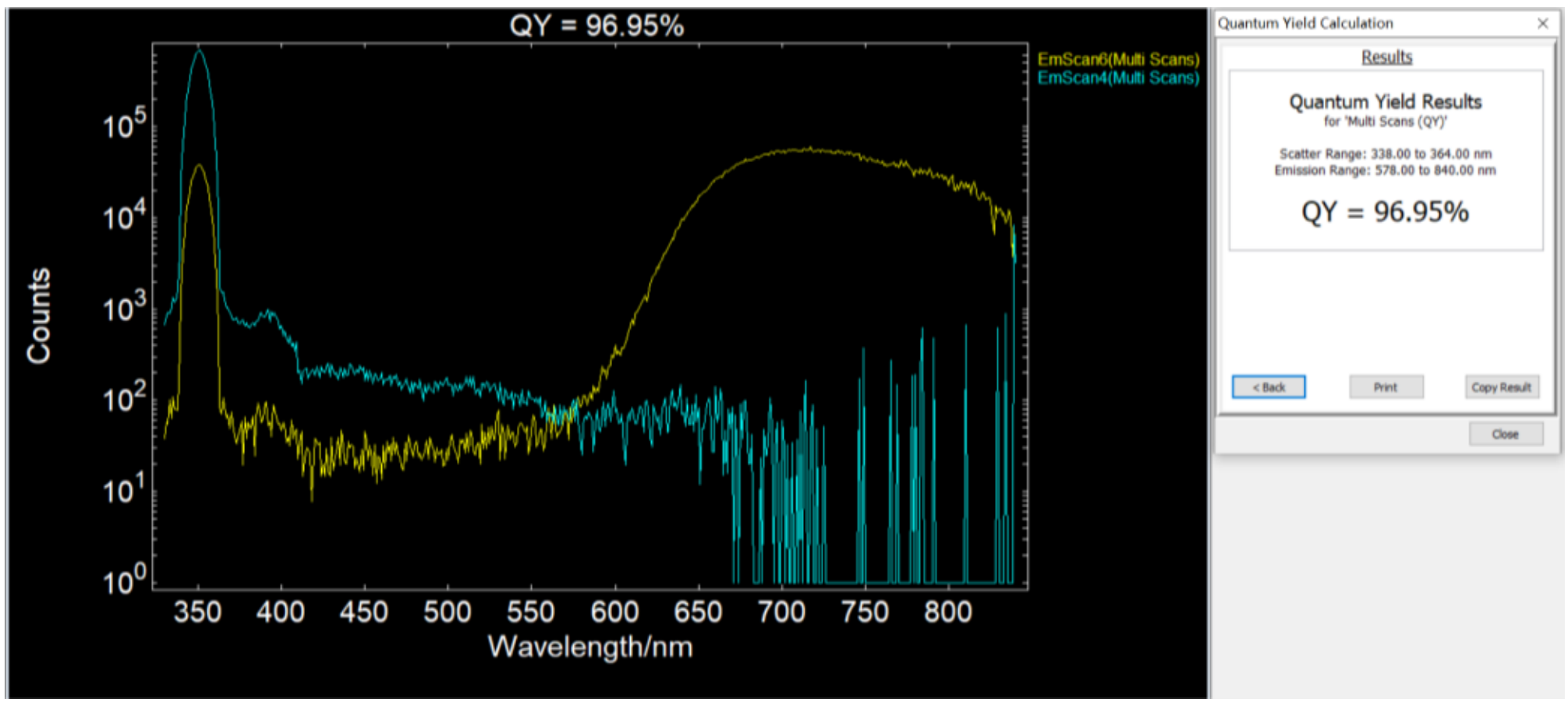

Cl-Br glass

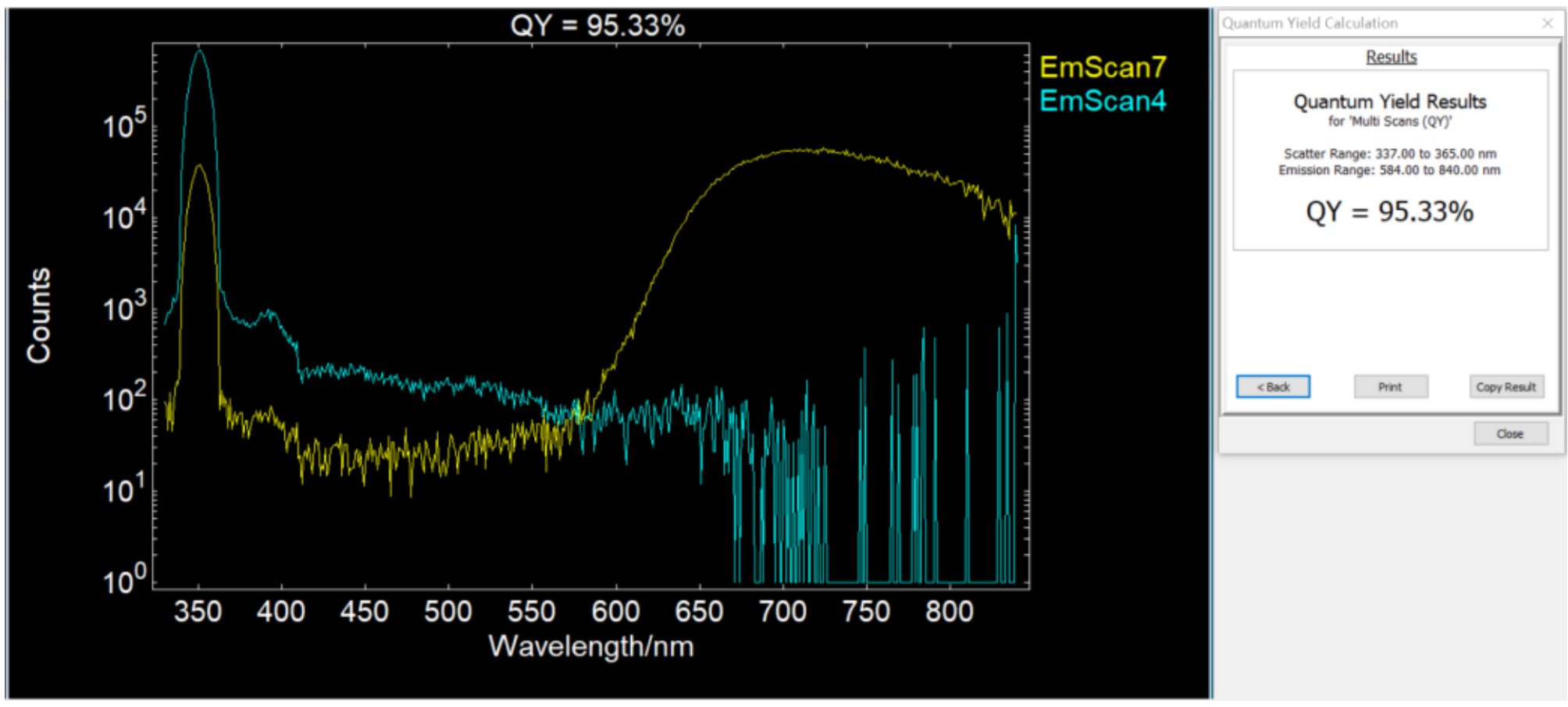

Cl-I glass

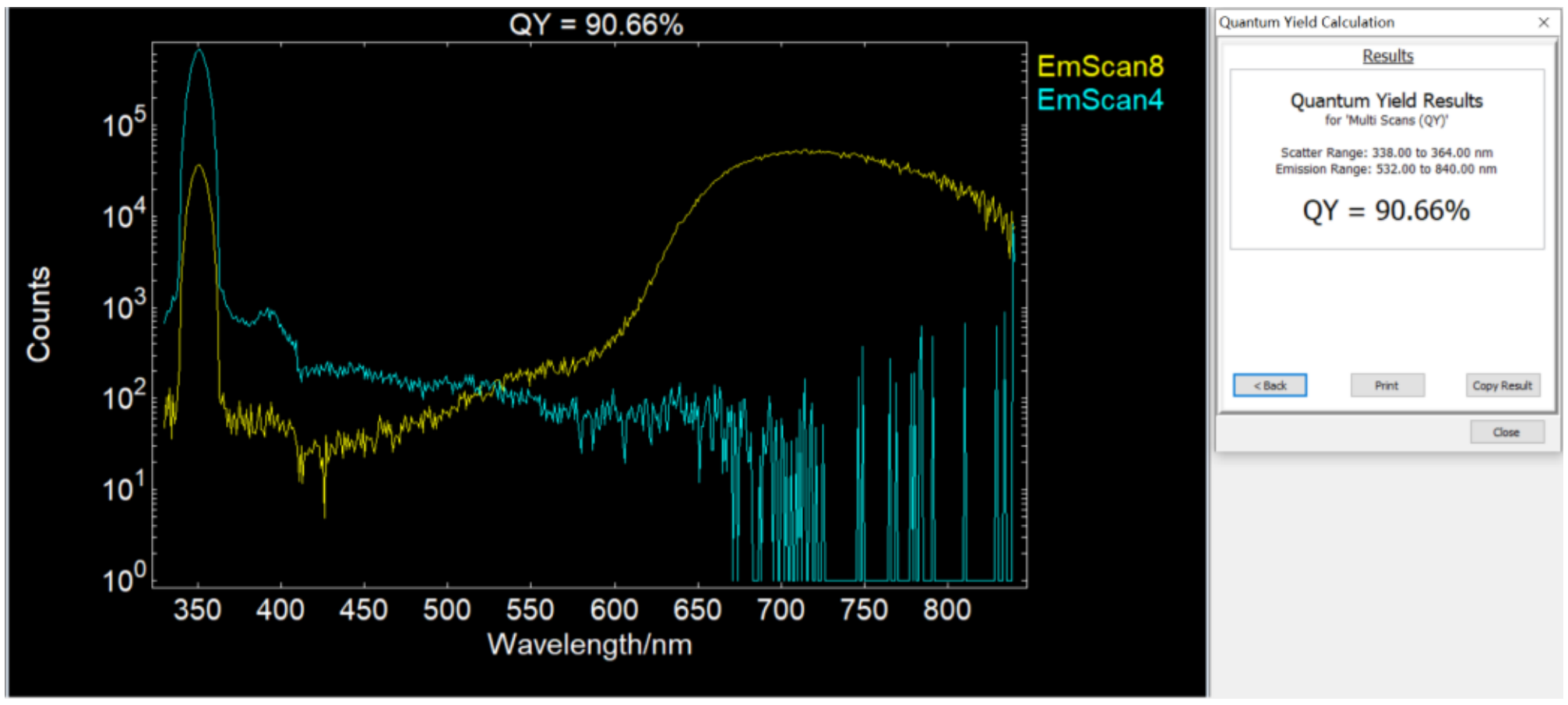

Supplementary Figure 13 | Photoluminescence quantum yield measurements of Cl-based Y-X glasses. Screenshots of the software interface showing the photoluminescence quantum yield measurement of Cl-Cl, Cl-Br and Cl-I glasses.

Br-Cl glass.

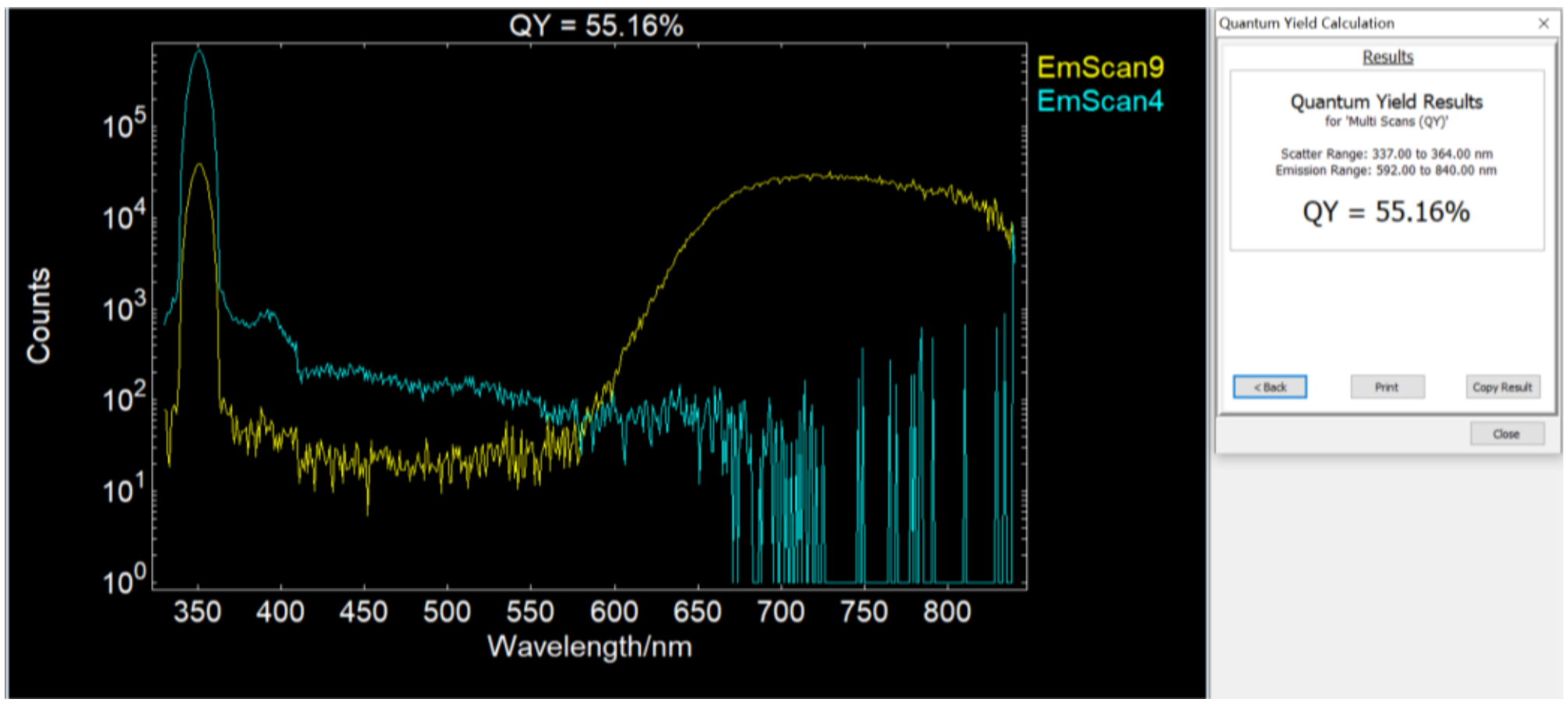

Br-Br glass

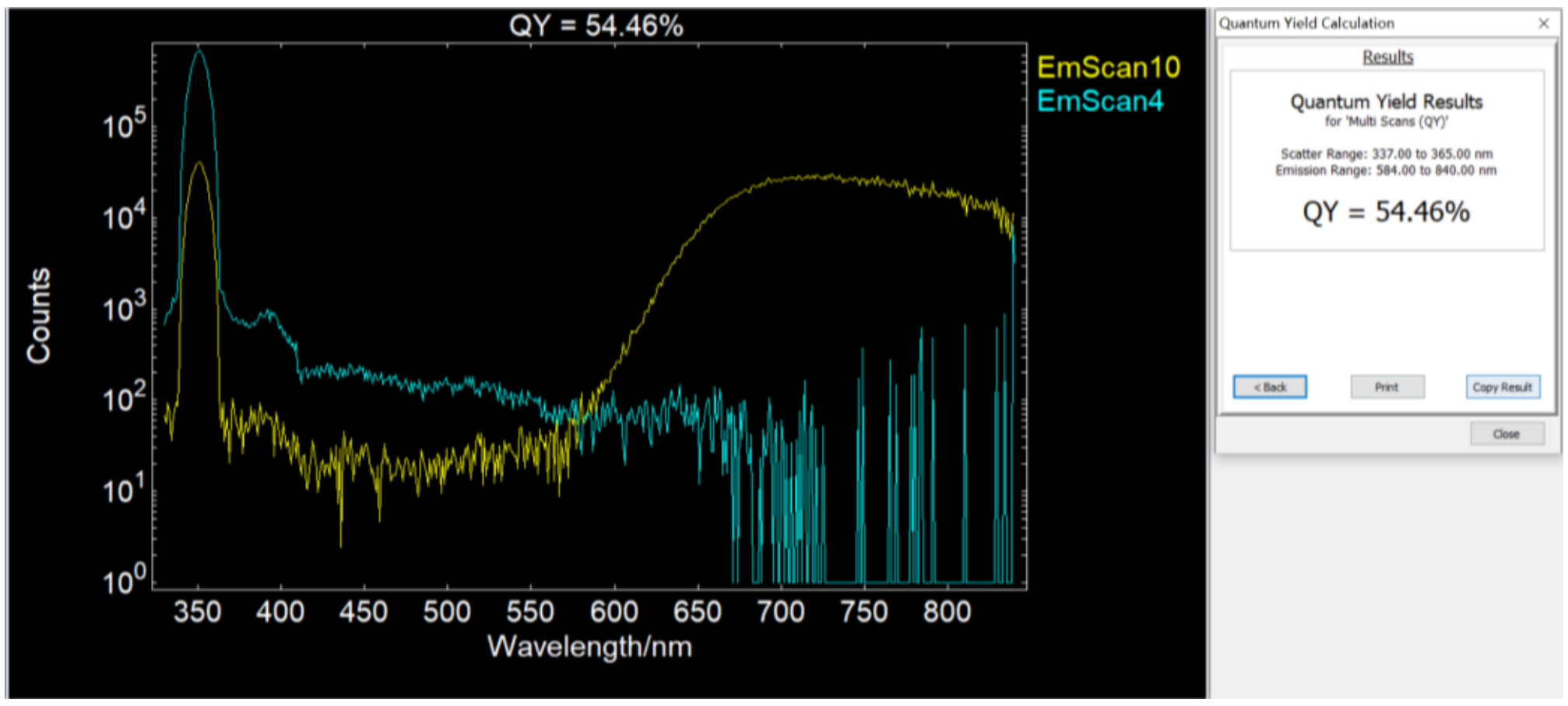

Br-I glass

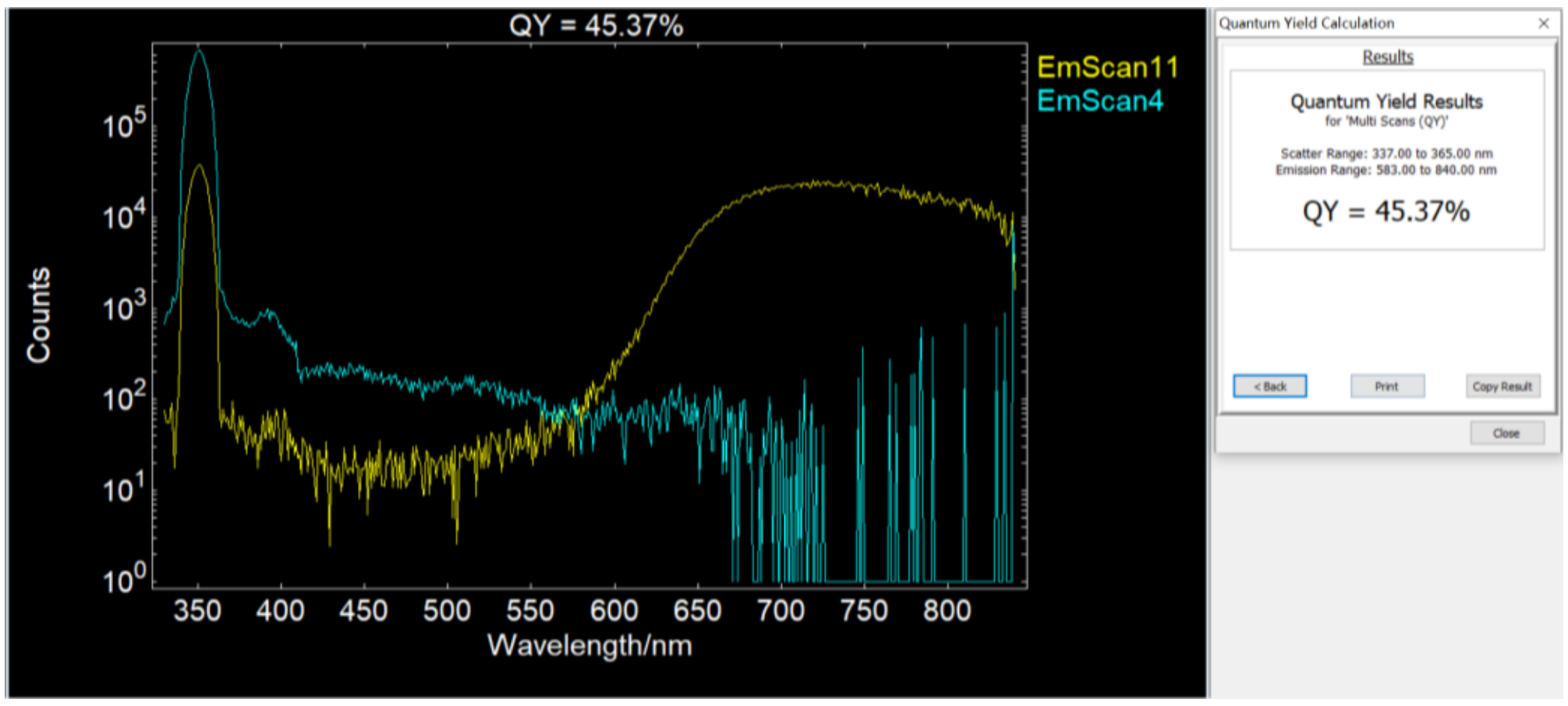

Supplementary Figure 14 | Photoluminescence quantum yield measurements of Br-based Y-X glasses. Screenshots of the software interface showing the photoluminescence quantum yield measurements of Br-Cl, Br-Br and Br-I glasses.

I-Cl glass

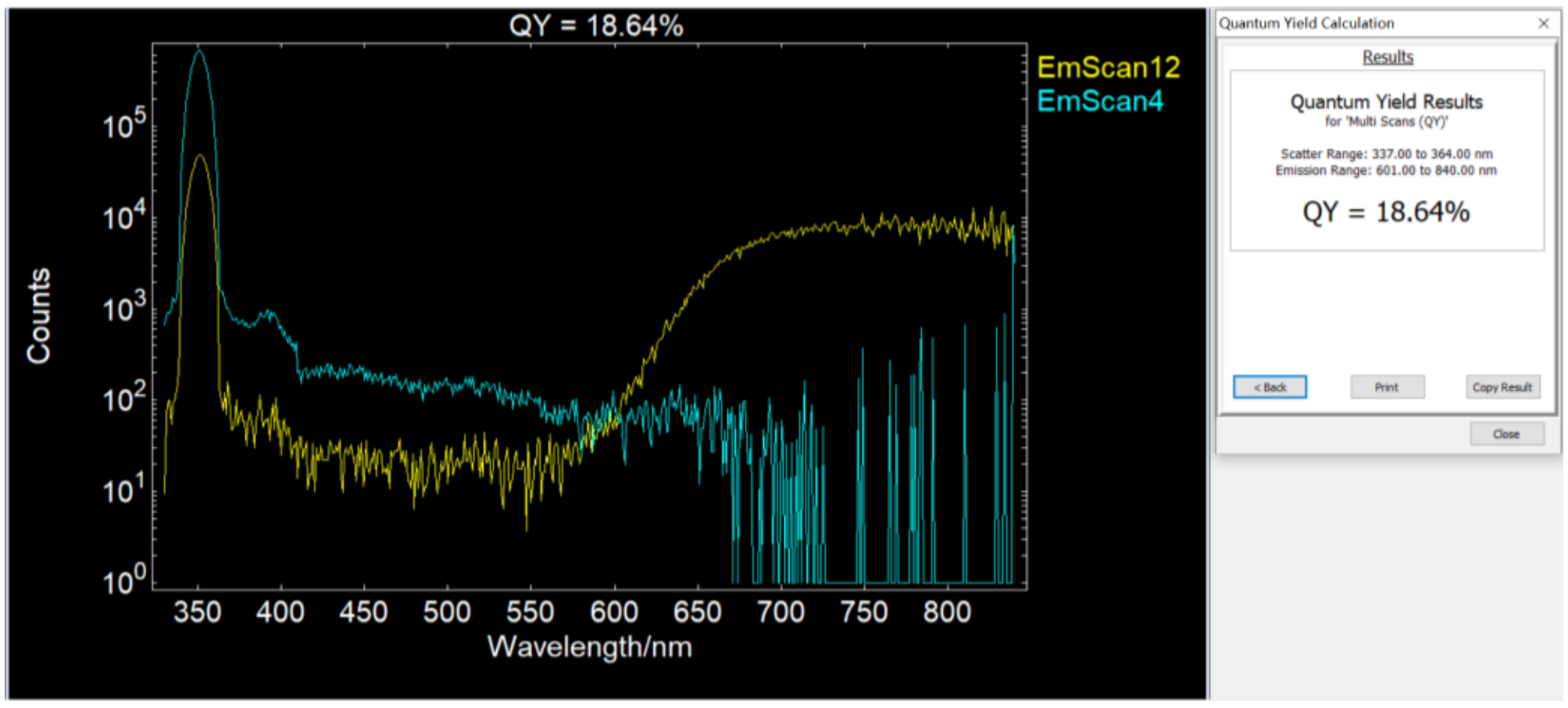

I-Br glass

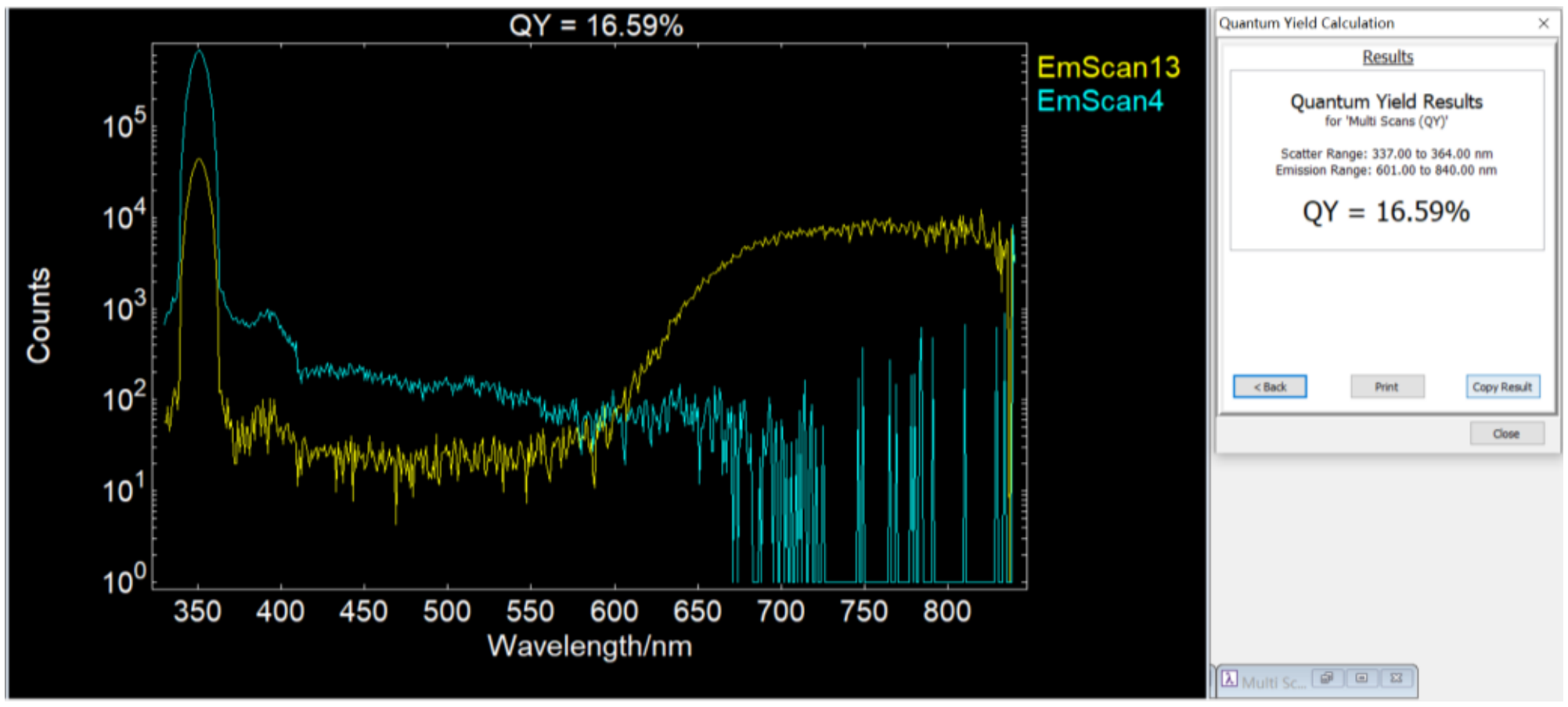

I-I glass

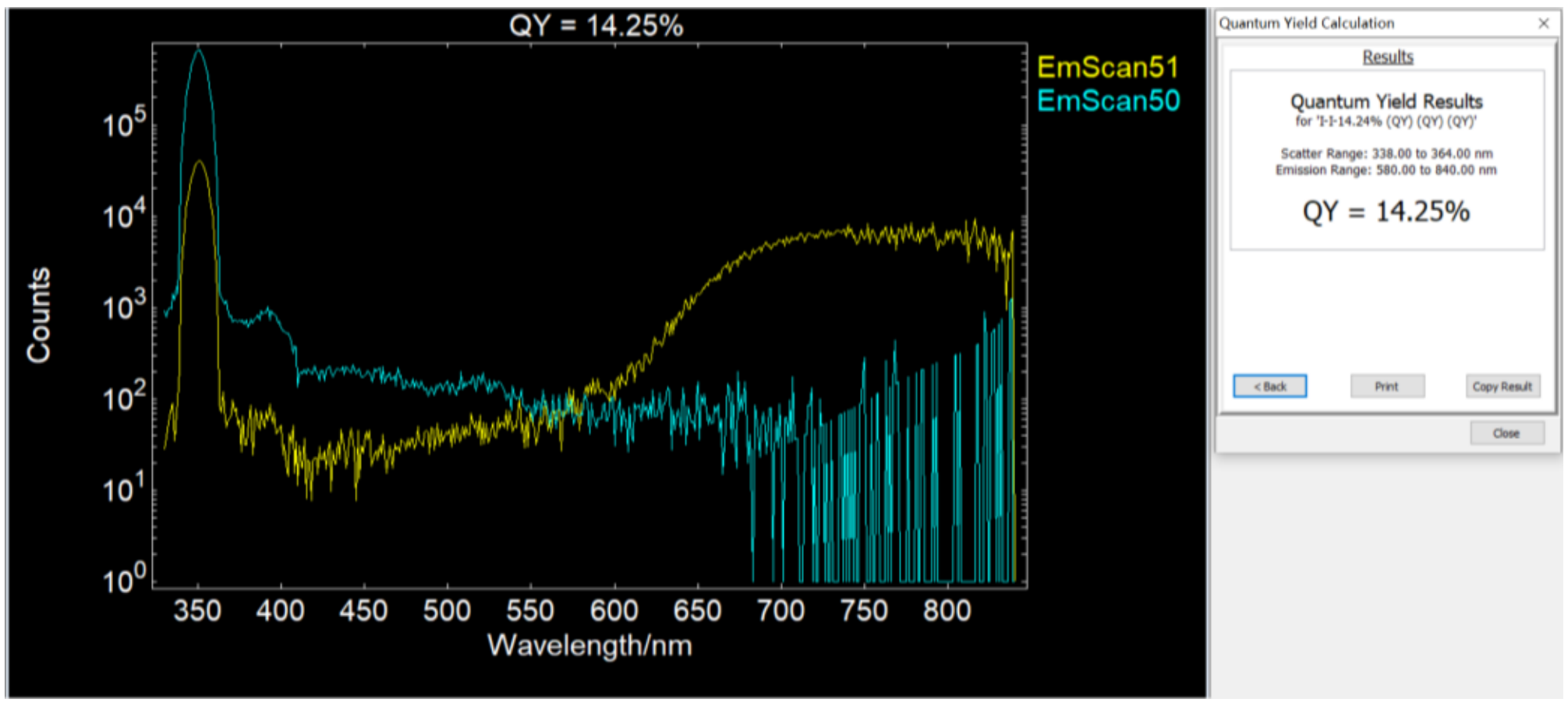

Supplementary Figure 15 | Photoluminescence quantum yield measurements of I-based Y-X glasses. Screenshots of the software interface showing the photoluminescence quantum yield measurements of I-Cl, I-Br and I-I glasses.

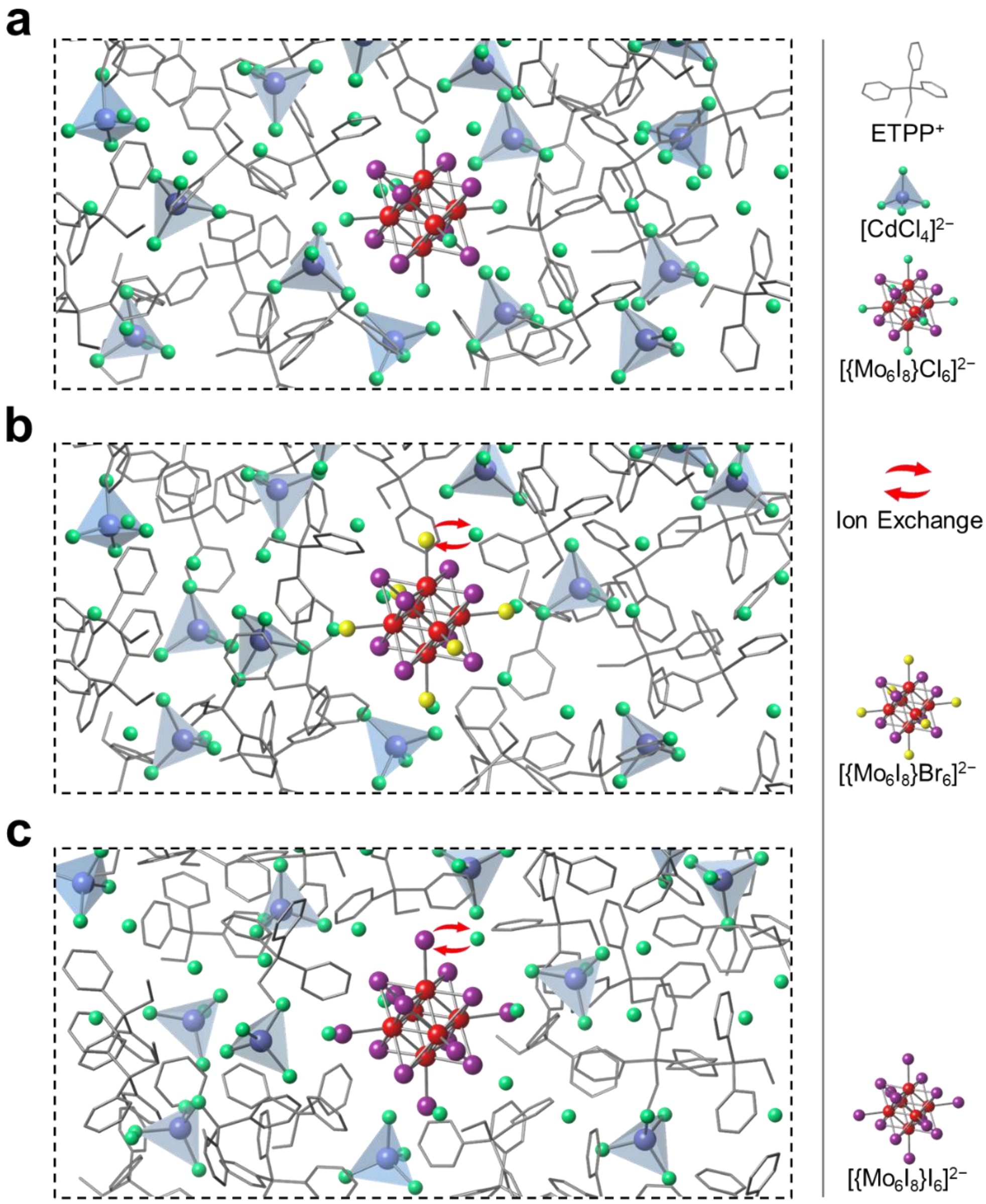

Supplementary Figure 16 | Halide redistribution during incorporation of cluster salts into chloride-based glass. Schematic illustration of the doping of $(ETP)_2[\{Mo_6I_8\}Cl_6]$, of $(ETP)_2[\{Mo_6I_8\}Br_6]$, and of $(ETP)_2[\{Mo_6I_8\}I_6]$ powders into the $(ETP)_2CdCl_4$ glass matrix. For $(ETP)_2[\{Mo_6I_8\}Br_6]$ and $(ETP)_2[\{Mo_6I_8\}I_6]$, all six external ligands are susceptible to attack and complete substitution by the high concentration of Cl^- ions in the matrix.

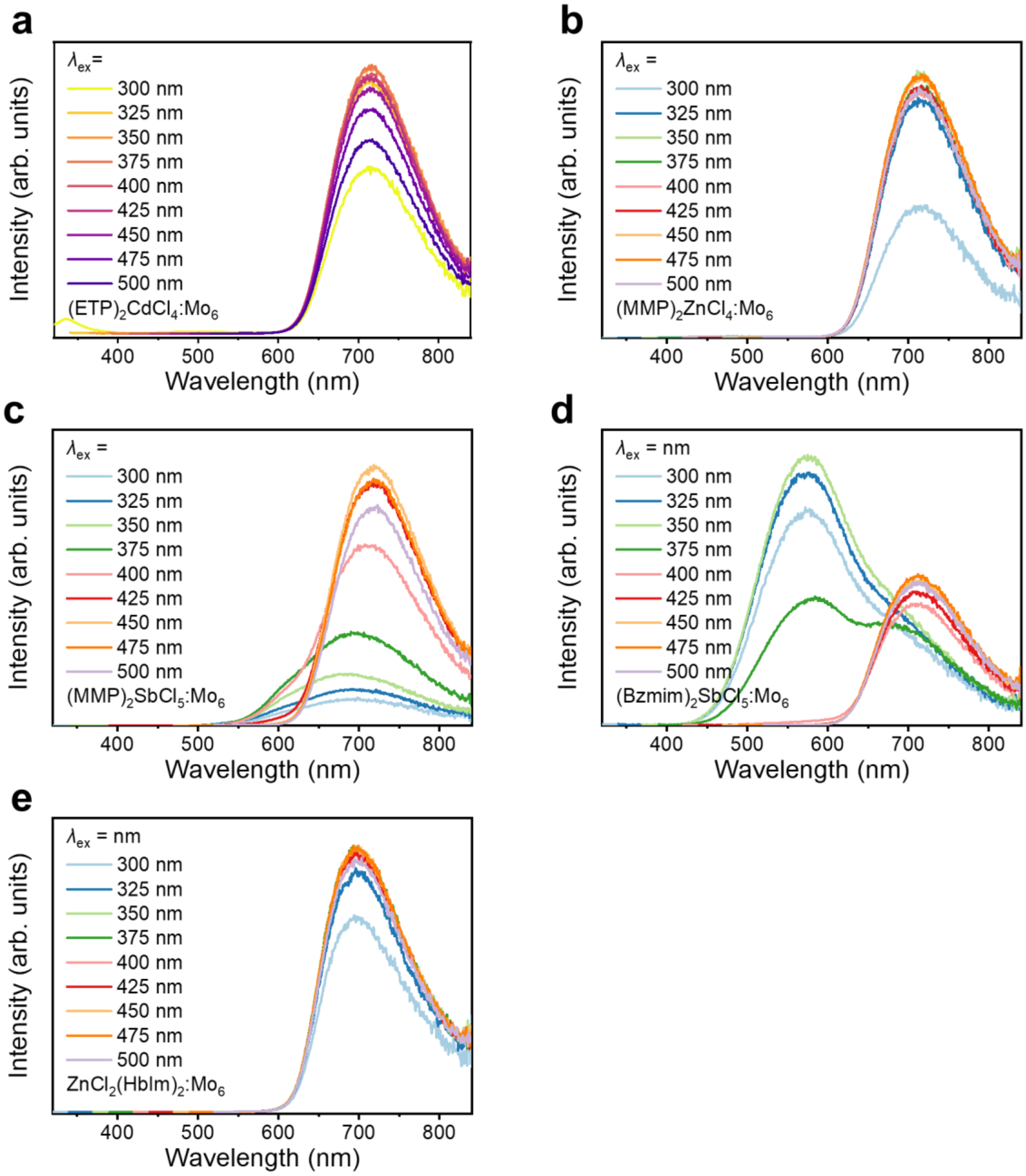

Supplementary Figure 17 | Excitation-dependent emission of cluster-doped glass matrices. Excitation-dependent PL spectra of five different glass matrices doped with $(ETP)_2[\{Mo_6I_8\}Cl_6]$, including **a,** $(ETP)_2CdCl_4$:Mo_6, **b,** $(MMP)_2ZnCl_4$:Mo_6, **c,** $(MMP)_2SbCl_5$:Mo_6, **d,** $(Bzmim)_2SbCl_5$:Mo_6, and **e,** $ZnCl_2(HbIm)_2$:Mo_6.

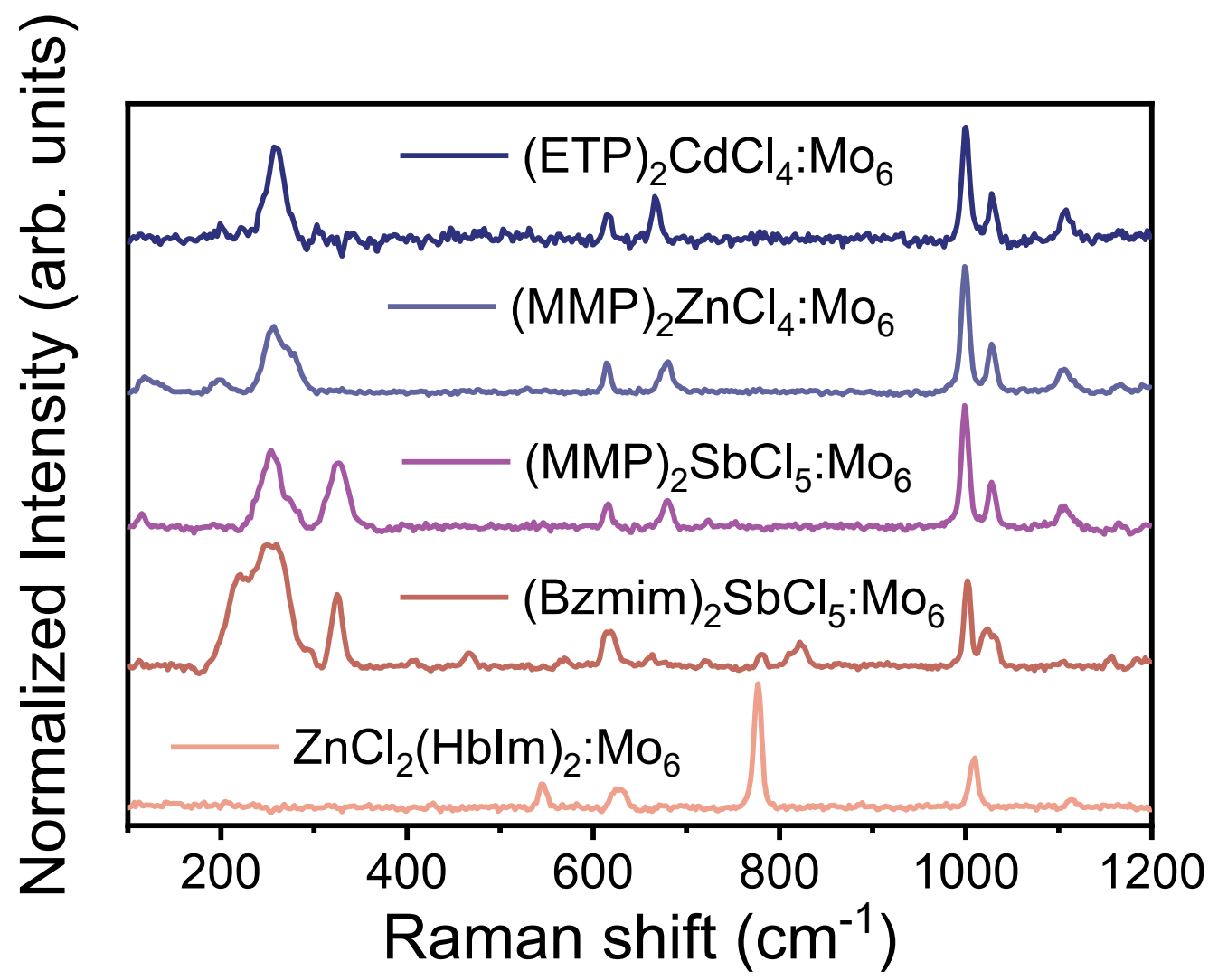

Supplementary Figure 18 | Raman characterization of cluster-doped glass matrices. Raman spectrum of five different glass matrices doped with $(ETP)_2[\{Mo_6I_8\}Cl_6]$.

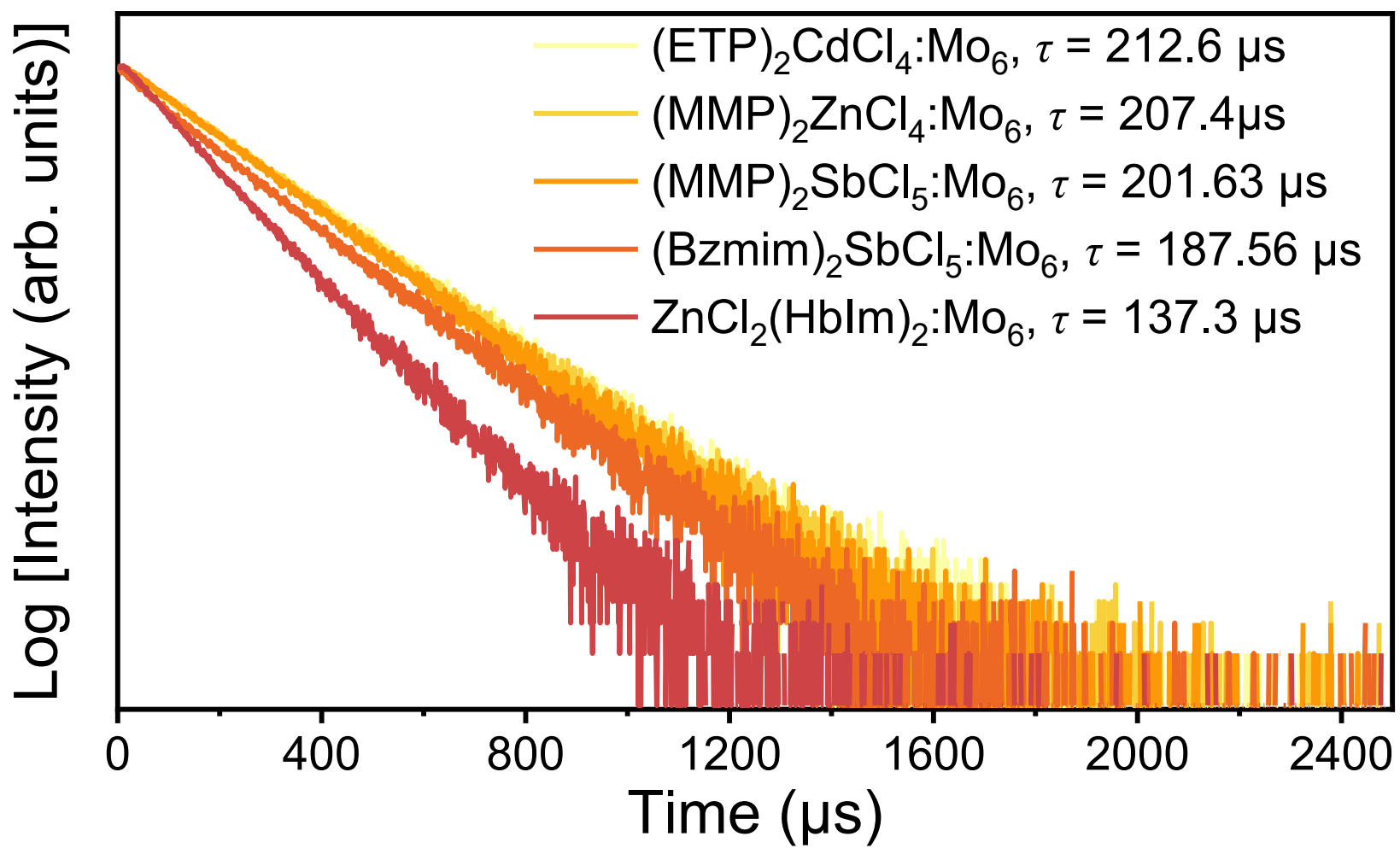

Supplementary Figure 19 | Photoluminescence decay dynamics of cluster-doped glass matrices. Decay curves of five different glass matrices doped with $(ETP)_2[\{Mo_6I_8\}Cl_6]$.

(MMP)$_2$ZnCl$_4$:Mo$_6$ glass

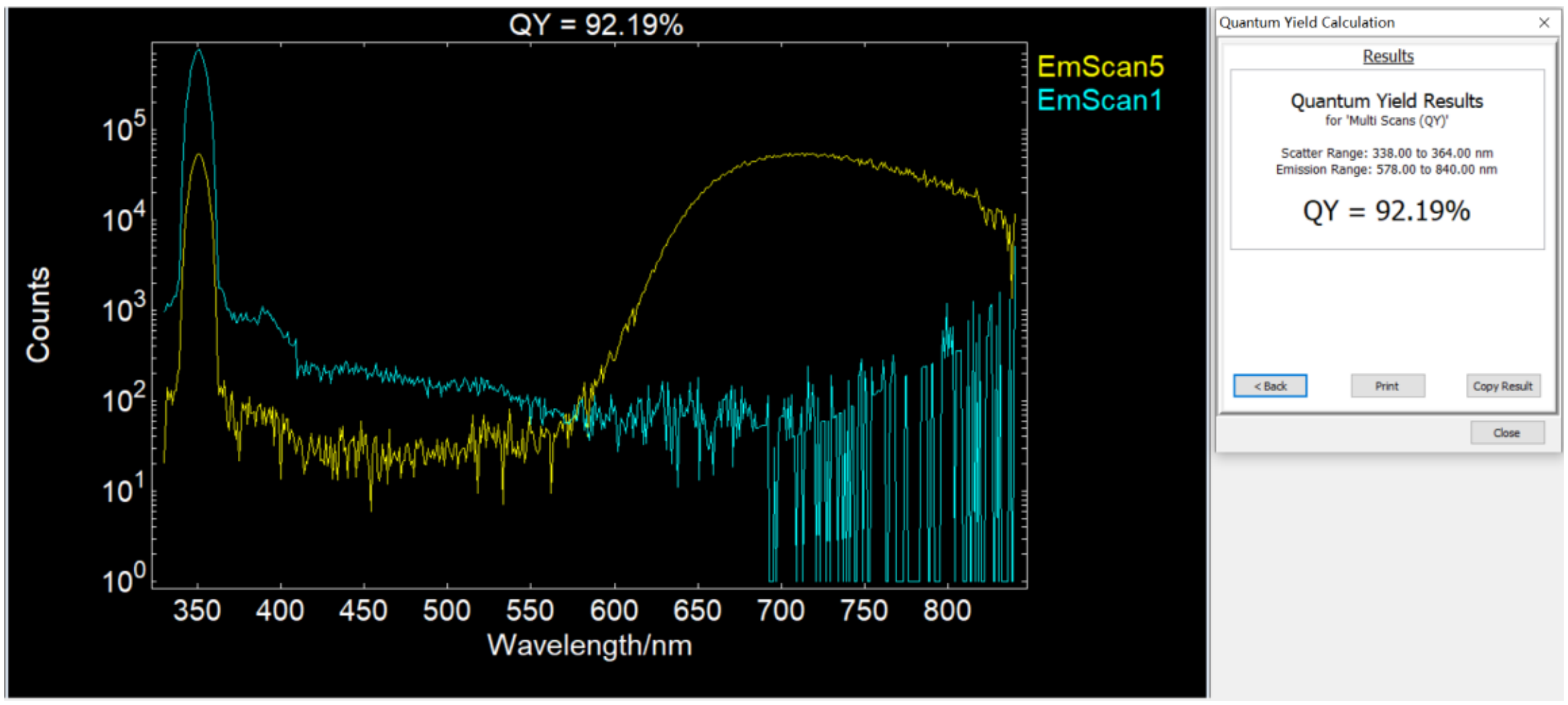

(MMP)$_2$SbCl$_5$:Mo$_6$ glass

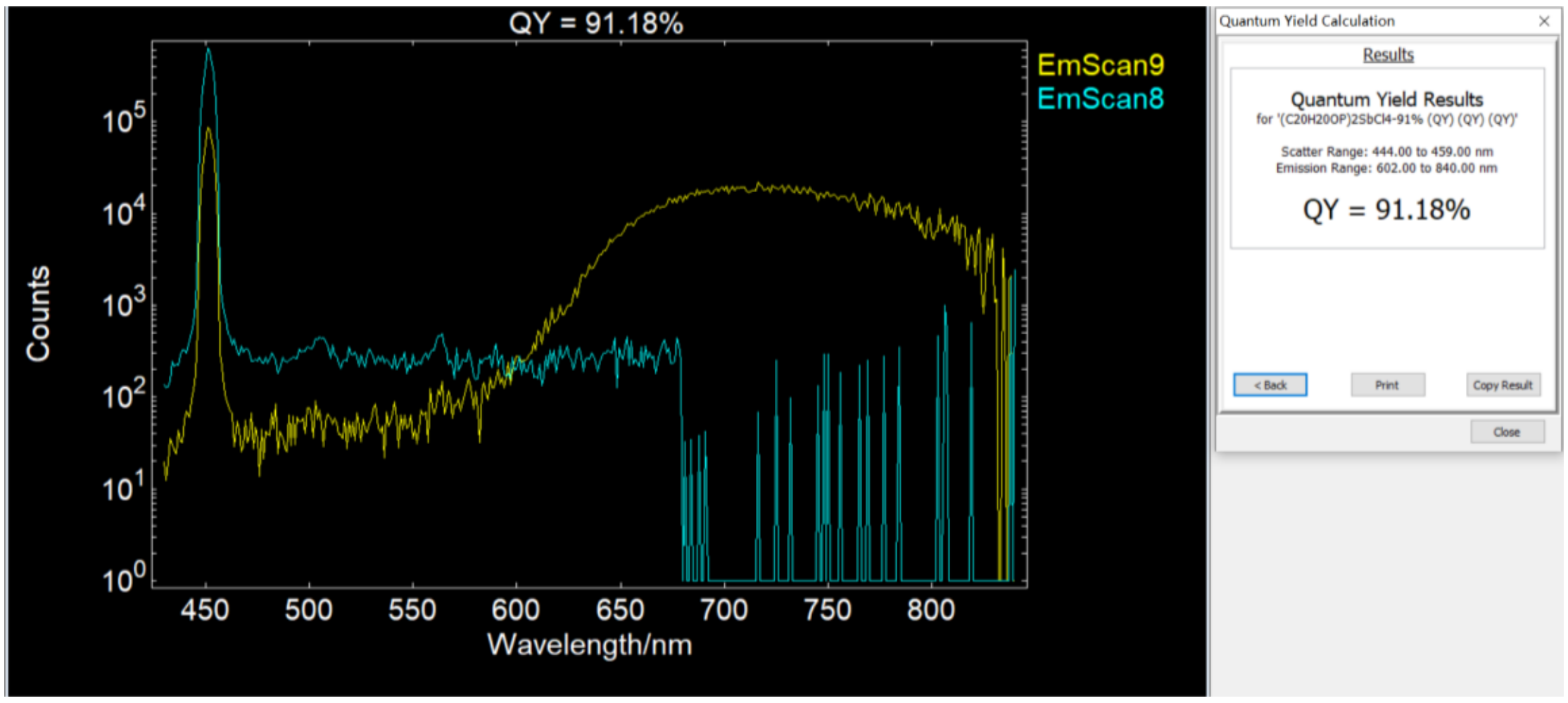

Supplementary Figure 20 | Photoluminescence quantum yield measurements of zinc- and antimony-based cluster glasses. Screenshots of the software interface showing the photoluminescence quantum yield measurement of (MMP)$_2$ZnCl$_4$:Mo$_6$ and (MMP)$_2$SbCl$_5$:Mo$_6$ glasses.

(Bzmim)$SbCl_5$:Mo_6 glass

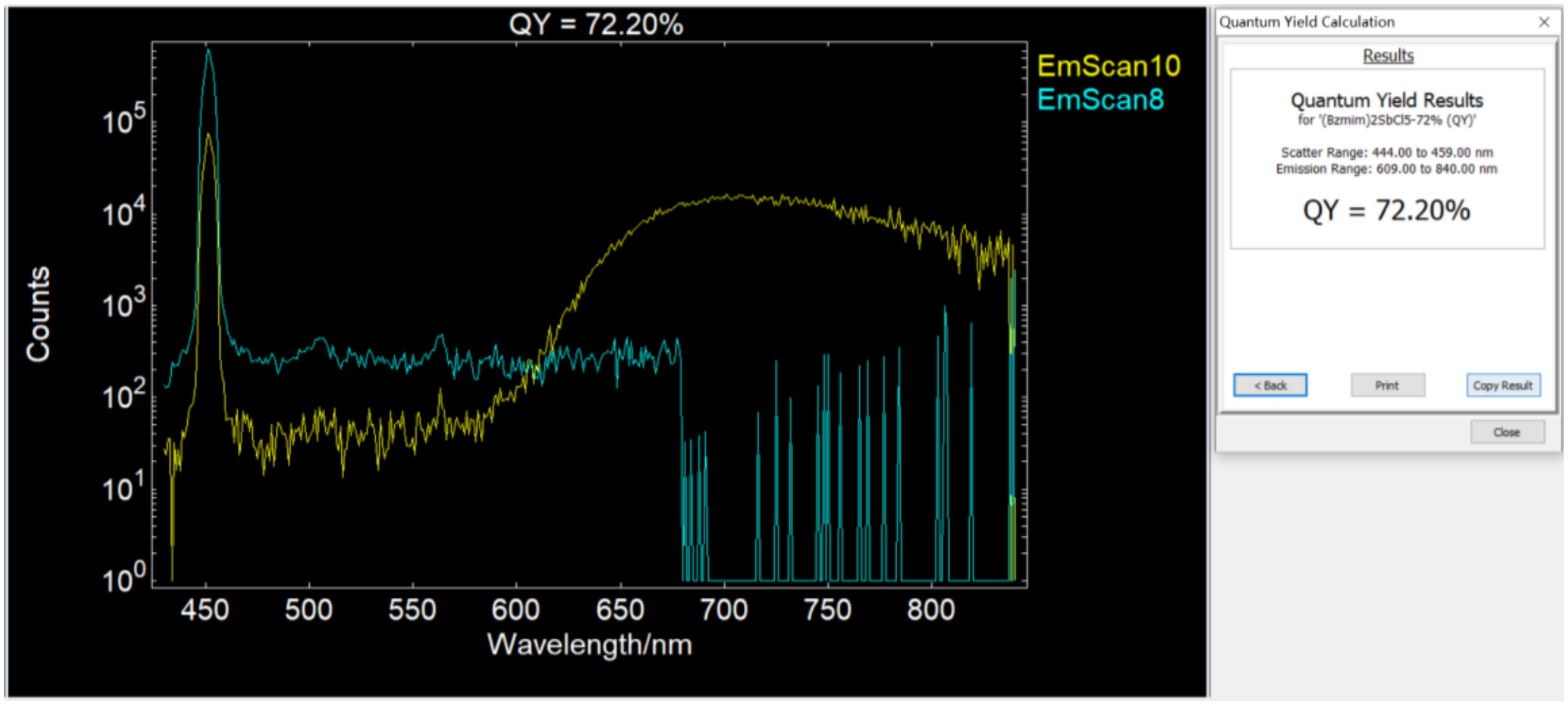

ZnCl$_2$(HbIm)$_2$:Mo_6 glass

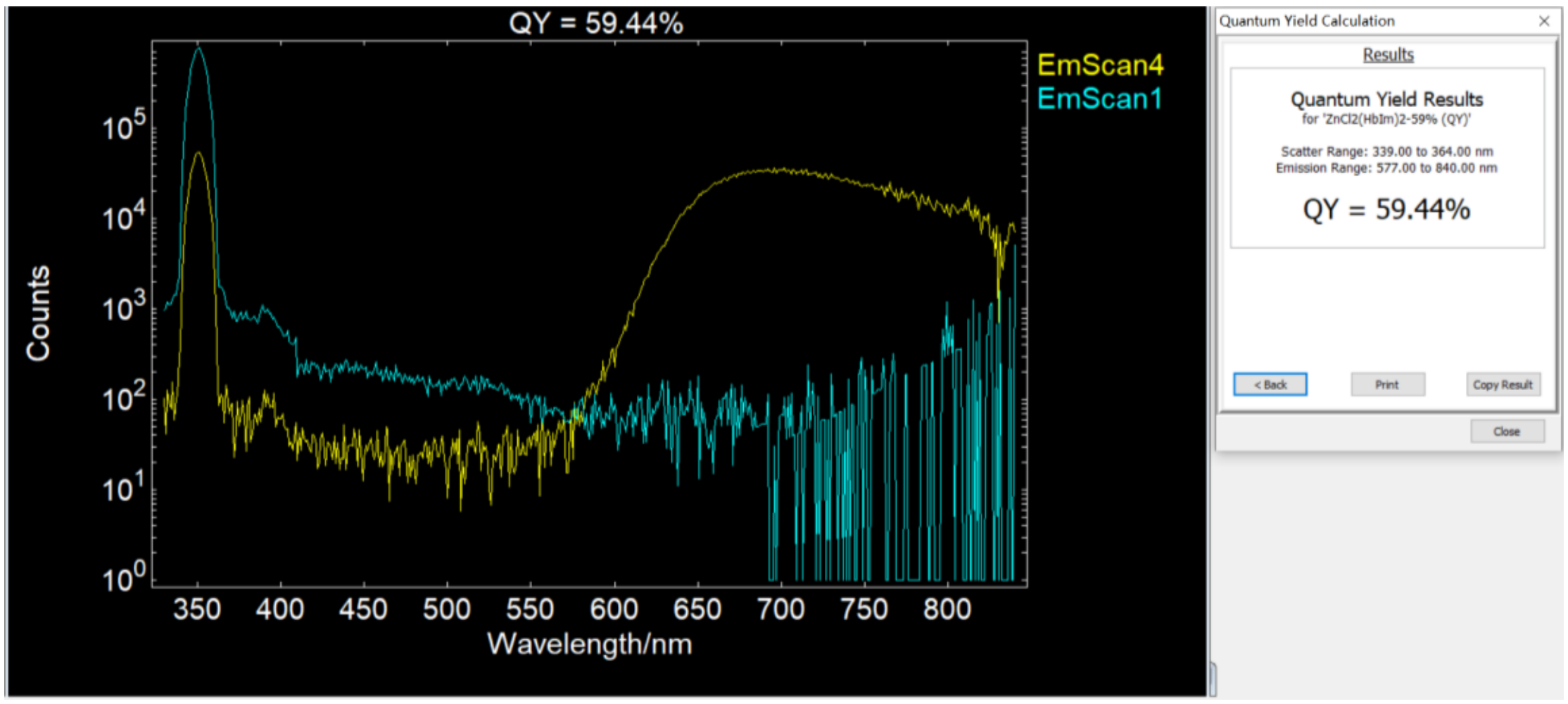

Supplementary Figure 21 | Photoluminescence quantum yield measurements of imidazolium-based cluster glasses. Screenshots of the software interface showing the photoluminescence quantum yield measurement of (Bzmim)$SbCl_5$:Mo_6 and ZnCl$_2$(HbIm)$_2$:Mo_6 glasses.

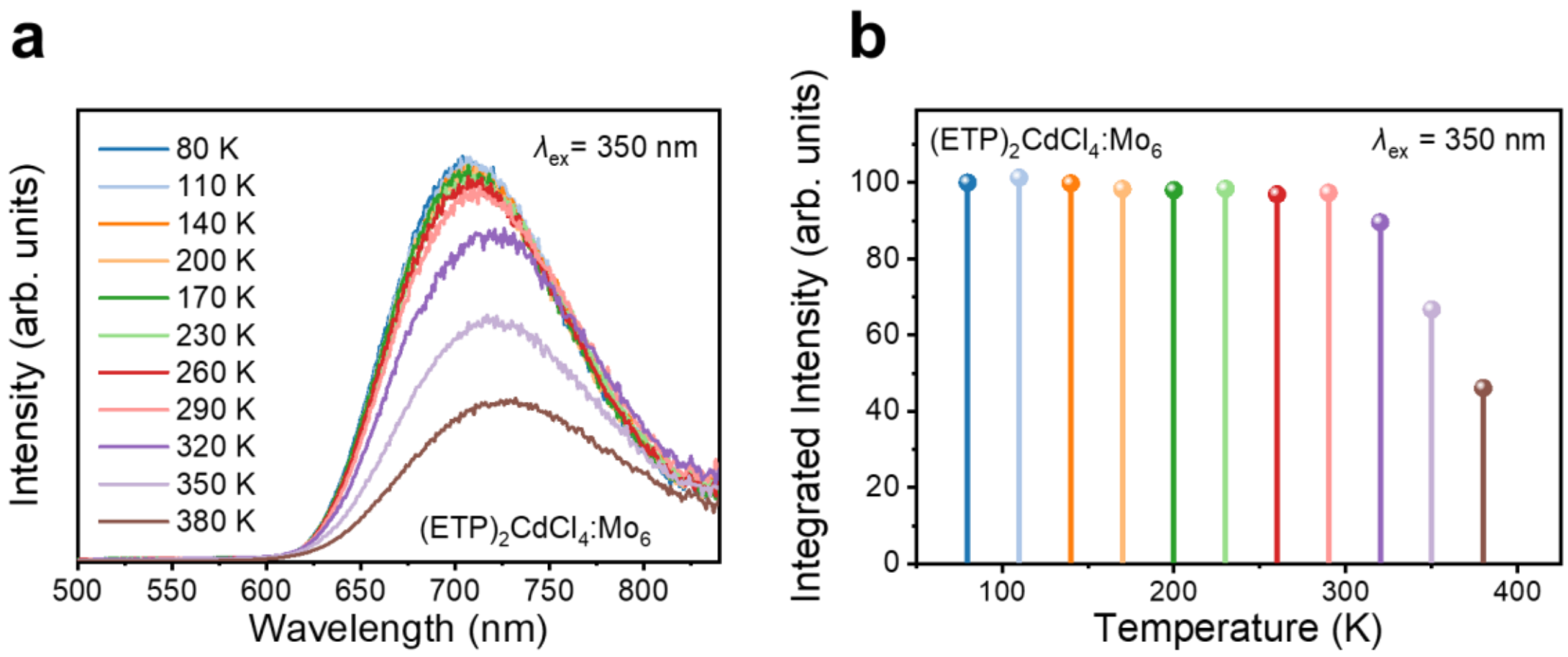

Supplementary Figure 22 | Temperature-dependent emission of molybdenum-cluster-doped cadmium chloride glass. a, Temperature-dependent PL spectra of $(ETP)_2CdCl_4:Mo_6$ glass and **b,** corresponding integrated emission intensity.

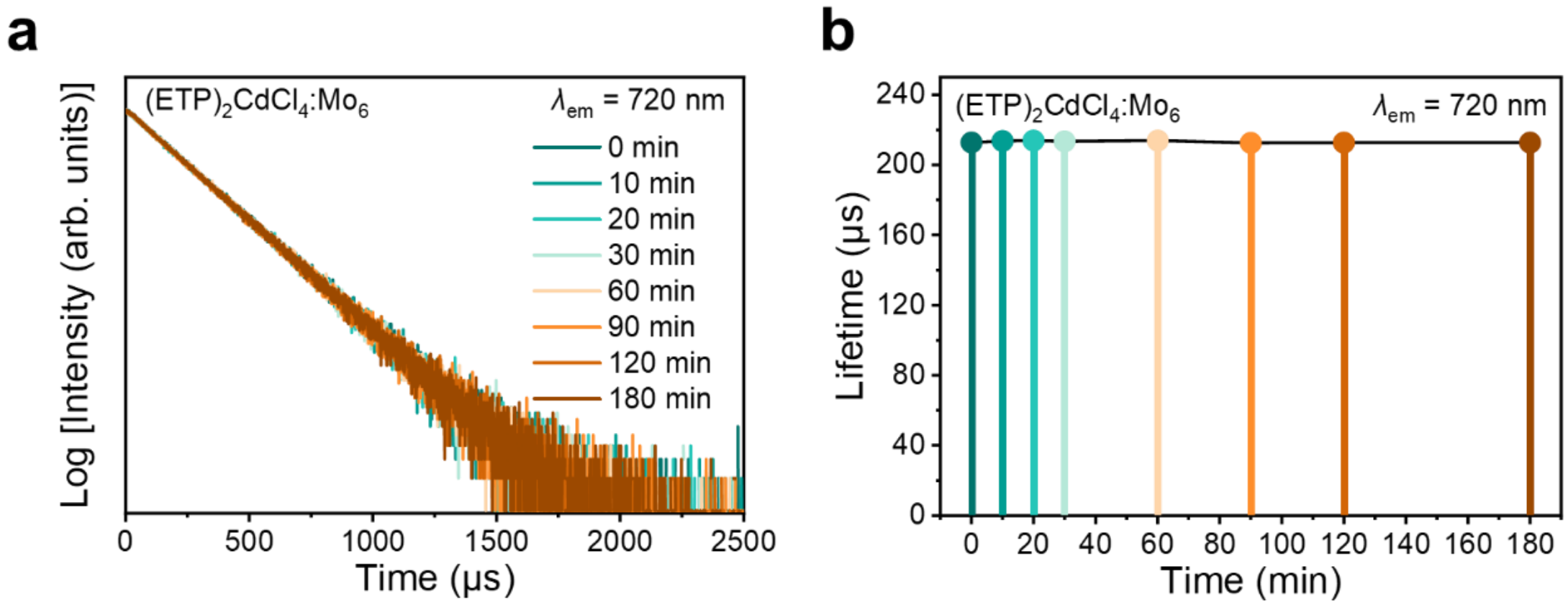

Supplementary Figure 23 | Air-exposure-dependent decay dynamics of molybdenum-cluster-doped cadmium chloride glass. a, Decay curves and **b,** corresponding lifetimes of $(ETP)_2CdCl_4:Mo_6$ glass as a function of air exposure time.

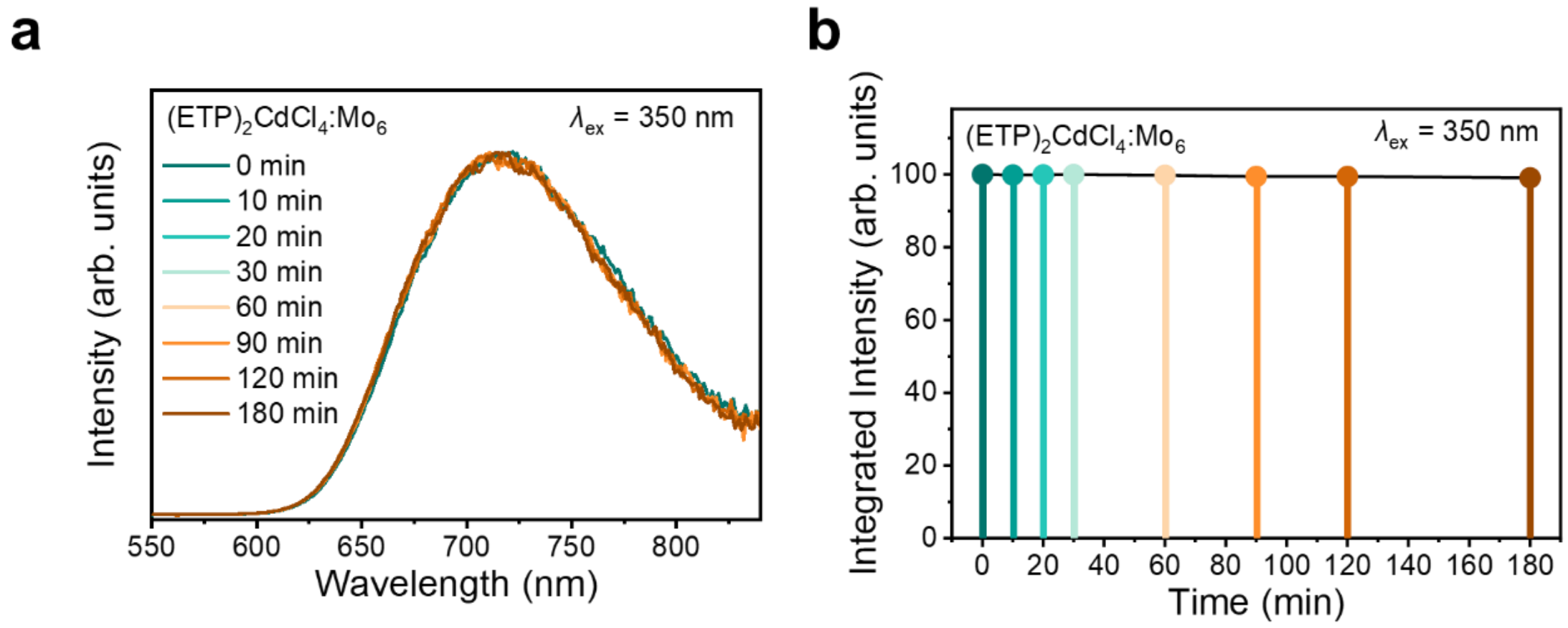

Supplementary Figure 24 | Air-exposure-dependent emission intensity of molybdenum-cluster-doped cadmium chloride glass. a, PL spectra and **b,** corresponding integrated intensities of $(ETP)_2CdCl_4:Mo_6$ glass as a function of air exposure time.

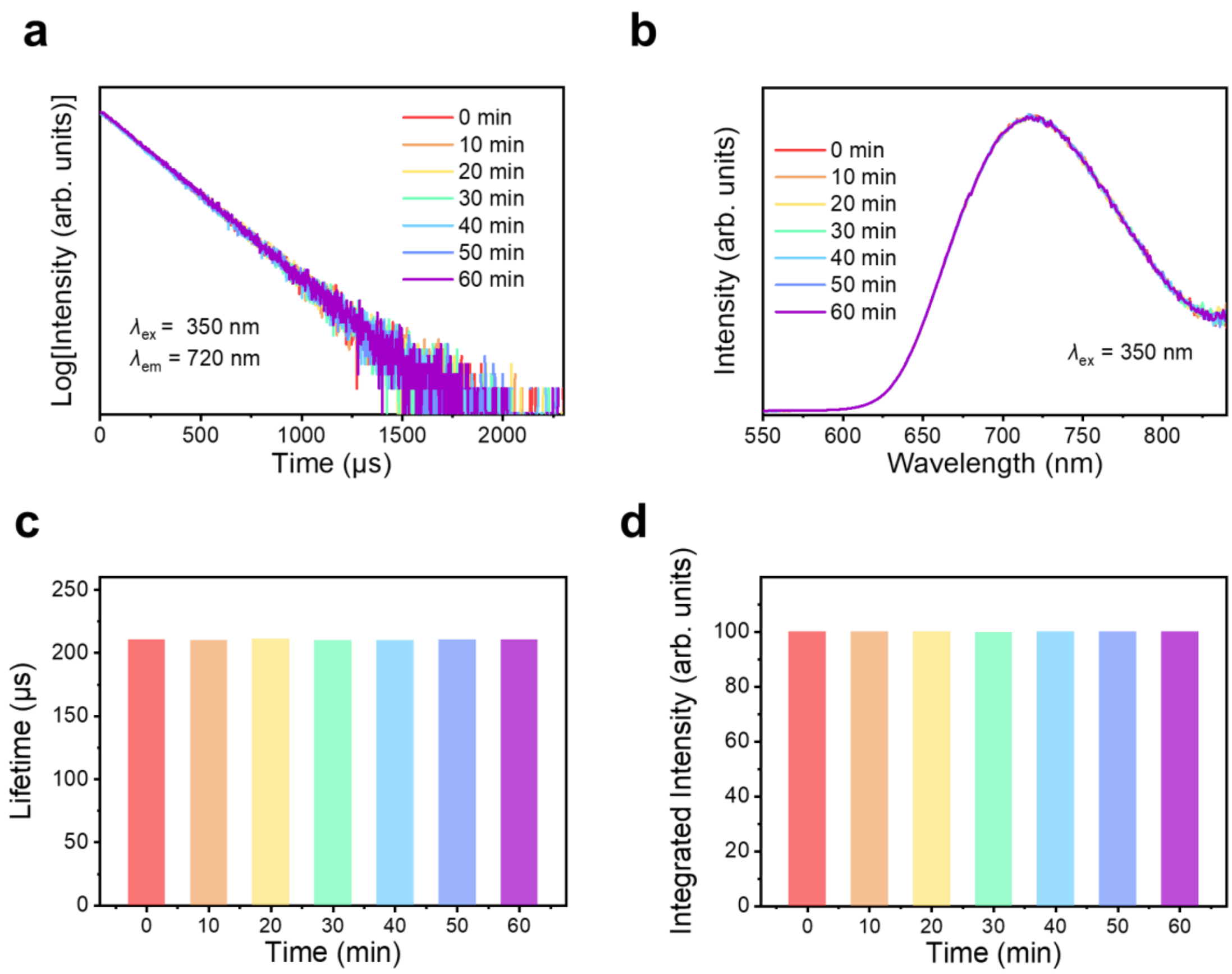

Supplementary Figure 25 | Photostability of $(ETP)_2CdCl_4$:Mo_6 under continuous 350 nm UV irradiation. a, Time-dependent PL decay curves. **b,** PL spectra recorded at different irradiation times. **c,** Evolution of the emission lifetime. (d) Evolution of the integrated PL intensity.

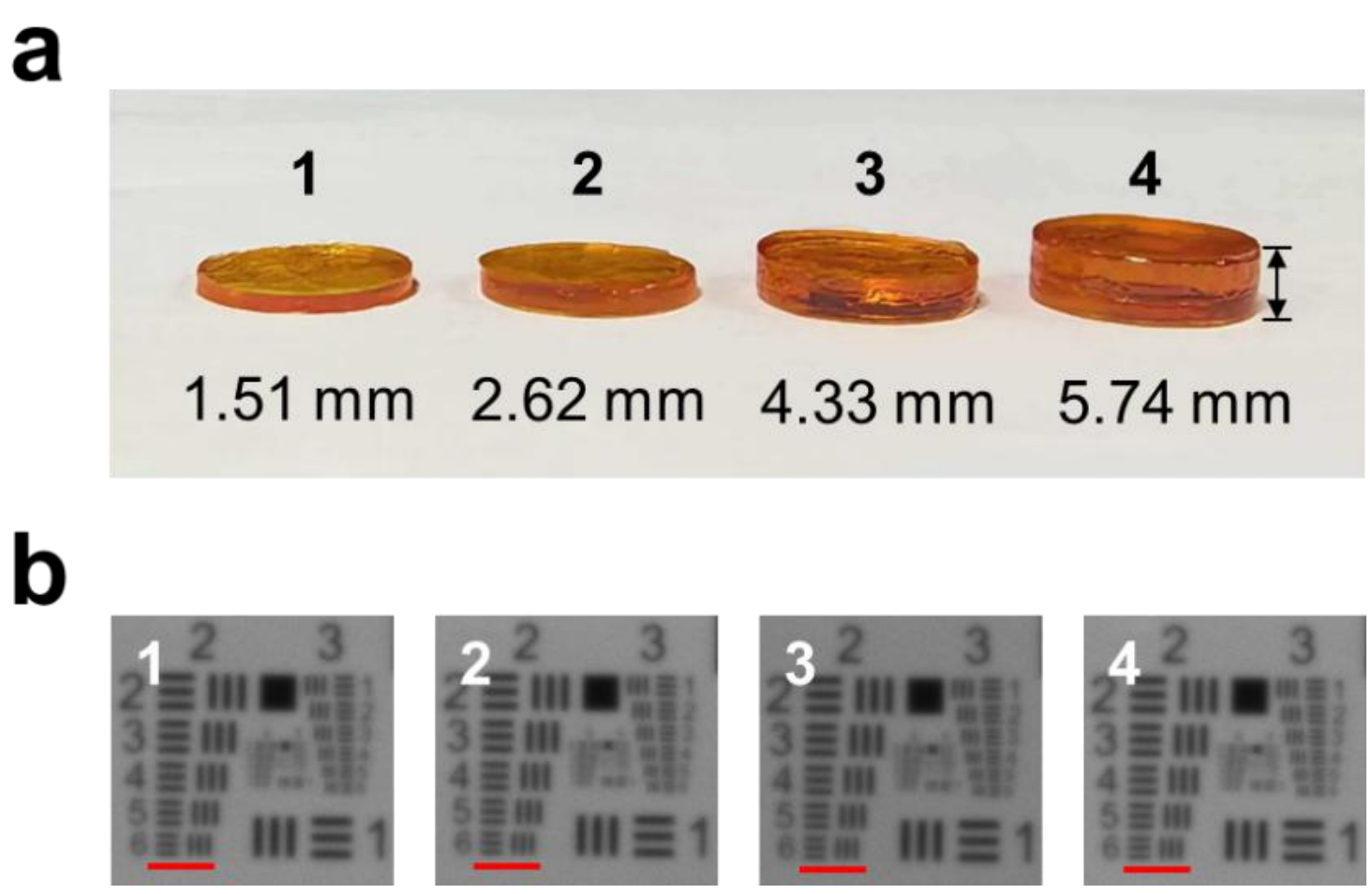

Supplementary Figure 26 | Thickness-dependent imaging performance of $(ETP)_2CdCl_4$:Mo_6 glasses. a, Photographs of $(ETP)_2CdCl_4$:Mo_6 glass samples with thicknesses of 1.51, 2.62, 4.33 and 5.74 mm, controlled by adjusting the precursor loading during melt-quenching preparation. **b,** NIR images of a USAF 1951 resolution target acquired using the corresponding glass samples as UV-excited NIR illumination sources. The red lines indicate the same reference region used for visual comparison of the imaging resolution.

Supplementary references

[1] M. Prévôt, M. Amela-Cortes, S. K. Manna, S. Cordier, T. Roisnel, H. Folliot, L. Dupont, Y. Molard, *J. Mater. Chem. C* **2015**, *3*, 5152-5161.

[2] Y. A. Vorotnikov, M. A. Mikhailov, K. A. Brylev, D. A. Piryazev, N. V. Kuratieva, M. N. Sokolov, Y. V. Mironov, M. A. Shestopalov, *Russ. Chem. Bull.* **2015**, *64*, 2591-2596.

[3] O. A. Efremova, M. A. Shestopalov, N. A. Chirtsova, A. I. Smolentsev, Y. V. Mironov, N. Kitamura, K. A. Brylev, A. J. Sutherland, *Dalton Trans.* **2014**, *43*, 6021-6025.

[4] Y. A. Vorotnikov, O. A. Efremova, I. N. Novozhilov, V. V. Yanshole, N. V. Kuratieva, K. A. Brylev, N. Kitamura, Y. V. Mironov, M. A. Shestopalov, *J. Mol. Struct.* **2017**, *1134*, 237-243.

[5] M. A. Mikhailov, K. A. Brylev, P. A. Abramov, E. Sakuda, S. Akagi, A. Ito, N. Kitamura, M. N. Sokolov, *Inorg. Chem.* **2016**, *55*, 8437–8445.

[6] M. Amela-Cortes, Y. Molard, S. Paofai, A. Desert, J. L. Duvail, N. G. Naumov, S. Cordier, *Dalton Trans.* **2016**, *45*, 237-245.

[7] M. N. Sokolov, M. A. Mihailov, E. V. Peresypkina, K. A. Brylev, N. Kitamura, V. P. Fedin, *Dalton Trans.* **2011**, *40*, 6375-6377.

[8] M. A. Mikhailov, K. A. Brylev, A. V. Virovets, M. R. Gallyamov, I. Novozhilov, M. N. Sokolov, *New J. Chem.* **2016**, *40*, 1162-1168.

[9] O. A. Efremova, Y. A. Vorotnikov, K. A. Brylev, N. A. Vorotnikova, I. N. Novozhilov, N. V. Kuratieva, M. V. Edeleva, D. M. Benoit, N. Kitamura, Y. V. Mironov, M. A. Shestopalov, A. J. Sutherland, *Dalton Trans.* **2016**, *45*, 15427-15435.

[10] M. N. Sokolov, M. A. Mikhailov, A. V. Virovets, K. A. Brylev, R. A. Bredikhin, A. M. Maksimov, V. E. Platonov, V. P. Fedin, *Russ. Chem. Bull.* **2013**, *62*, 1764-1767.

[11] M. N. Sokolov, M. A. Mikhailov, K. A. Brylev, A. V. Virovets, C. Vicent, N. B. Kompankov, N. Kitamura, V. P. Fedin, *Inorg. Chem.* **2013**, *52*, 12477-12481.

[12] I. V. Kashnik, B. Yang, N. Dumait, M. Cordier, K. A. Brylev, P. A. Bouit, Y.

Molard, *Adv. Opt. Mater.* **2024**, *12*, 2400781.

[13] I. V. Kashnik, J. Rebours, N. Dumait, K. A. Brylev, Y. Molard, *J. Mater. Chem. C* **2024**, *12*, 8398-8407.

[14] S. Akagi, S. Fujii, N. Kitamura, *Dalton Trans.* **2018**, *47*, 1131-1139.
