# Supplementary material for: Near-unity near-infrared emission from Mo6 cluster doped hybrid halide glasses via halide-ligand and matrix engineering
Source: Nat Commun. 2026 Jul 22;17:8947. doi: 10.1038/s41467-026-75934-5 (PMC13503768; doi:10.1038/s41467-026-75934-5)
Supplement: Supplementary file 2 [file 41467_2026_75934_MOESM2_ESM.pdf]

nature portfolio

# Peer Review File

# Near-Unity Near-Infrared Emission from $Mo_6$ Cluster doped Hybrid Halide Glasses via Halide-Ligand and Matrix Engineering

Corresponding Author: Dr Peiqing Cai

**This file contains all reviewer reports in order by version, followed by all author rebuttals in order by version.**

Version 0:

Reviewer comments:

Reviewer #1

(Remarks to the Author)
This manuscript reports a zero-dimensional hybrid metal halide glass system incorporating Mo6 clusters, and systematically investigates its near-infrared photoluminescence properties. Via materials synthesis, structural characterization, and spectroscopic investigations, the authors demonstrate broadband near-infrared emission and discuss its promising applications in optoelectronic devices and imaging. This work is relevant to the development of high-performance near-infrared luminescent materials and will be of broad interest to readers of Nature Communications. Overall, the manuscript presents relatively systematic experimental work, and multiple characterization results support the main conclusions. However, several aspects related to data presentation, mechanistic interpretation, and experimental details require further clarification and improvement to enhance the rigor and completeness of the study. The following comments are provided for the authors' revision.
1. In the structural analysis section, the authors mainly discuss the arrangement of the crystal structure, while the potential influence of variations in Mo–X bond lengths on the electronic structure and luminescent properties is not sufficiently explored. A more quantitative analysis of structural parameters is recommended to strengthen the discussion of the structure–property relationship.
2. The molybdenum cluster samples with different halide ligands exhibit noticeably different absorption coefficients, yet the manuscript provides limited discussion regarding the underlying reasons. Further analysis of the potential influence of halogen atomic orbital characteristics on the absorption cross-section is recommended.
3. The temperature-dependent PL measurements only extend to 290 K. Considering that the material may operate under higher temperatures in practical applications, it is suggested to extend the measurement range or provide additional discussion regarding the thermal stability of the material at elevated temperatures.
4. Although relatively high IQE and EQE values are reported, the manuscript lacks repeated experiments or error analysis. Providing averaged values along with standard deviations from multiple measurements would improve the reliability of the reported data.
5. The resolution test was conducted using a USAF 1951 target; however, the measurement distance and optical system configuration are not specified. These key experimental parameters should be provided to improve the completeness of the experimental description.
6. It would be useful to provide spectral data for the glass samples after long-term storage in air in order to evaluate the environmental stability of the material. For potential device applications, additional photostability tests under continuous illumination are recommended to verify the practical applicability of the materials.
7. Molecular hybrid glasses and the wide photoemission properties are hot topics in materials and chemistry. To arouse a broad interest from readership in this field, some recent related works on recent fabrications of hybrid metal halide glass (Matter, 2024, 7, 1950; Acc. Chem. Res., 2025, 58, 3010; Angew. Chem. Int. Ed. 2026, e8889513) and high-performance luminescent applications (Nat. Commun. 2023, 14, 1654; Angew. Chem. Int. Ed. 2025, 64, e202420139; Sci. China Mater. 2024, 67, 3531) could be included as references.
8. A comparison with other types of near-infrared luminescent materials (such as rare-earth ions, transition metal ions, and quantum dots, please checking Angew. Chem. Int. Ed. 2025, 64, e202505322) is recommended to more comprehensively demonstrate the advantages and characteristics of the present work.

Reviewer #2

(Remarks to the Author)
The manuscript by Feng et al. reports on the preparation and study of the photoluminescence of cluster compounds with general formula (ETP)2[Mo6I8X6] (X=Cl, Br, and I). The compounds were further embedded in glass matrices leading to increase in photoluminescence quantum efficiency in air atmosphere.

Overall, the study is well conducted and the manuscript is well written, but there is a clear lack of novelty, as it is already known for almost 15 years that Mo6 clusters especially those with iodine inner ligands can exhibit very high quantum yields of photoluminescence, close to unity, even in solution (10.1039/c3dt32863e; 10.1021/ic502144z; 10.1021/acs.inorgchem.9b02569). Most of this study is focused on features of these cluster that were previously demonstrated by other authors (temperature dependence of the photoluminescence, oxygen quenching, DFT calculations of the excited states). The main interest of the article is the ability to limit quenching by oxygen in the solid-state which can dramatically reduce the quantum efficiency in air atmosphere. However, it is not a newly discovered feature of Mo6 cluster compounds (10.1039/C6DT02863B) and no suitable explanation can be found in the manuscript as to why these specific compounds show limited oxygen quenching.

For these reasons I do not recommend publication of the manuscript in Nature Communications and I suggest submission to a more specialized journal.

Reviewer #3

(Remarks to the Author)
In this study, the authors designed and synthesized a series of isostructural molybdenum clusters (ETP)2[{Mo6I8}X6] (X = Cl, Br, I) via ligand exchange method in high-temperature ionic liquid, and revealing systematic correlations between outer ligands and the electronic and luminescent properties of the {Mo6I8}4+ core. Further, due to the suppresses cluster vibrations and nonradiative transitions, embedding [{Mo6I8}Cl6]2– cluster into (ETP)2CdCl4 glass matrix markedly enhances cluster dispersion and photostability, achieving 96.95% 43 IQE and 91.43% EQE. This study delivers a timely and valuable contribution to the field of next-generation high-efficiency NIR light sources, particularly in the context of structural modulation strategies aimed at enhancing performance of octahedral molybdenum cluster luminescent materials. The reviewer thinks this work is interesting and is worthy of publication. However, the following issues should be well addressed:
1. One of the key points in this manuscript is about the preparation of the hybrid metal halide glasses, and some of the recent advances in this topic should be described, as reported in Advanced Functional Materials, 2026, 36(23) e21093.; Matter, 2025, 102214.; Chemical Science, 2023, 14, 12238.
2. Figure S1 demonstrates the absorption spectra, not diffuse reflectance spectra.
3. In Figure 1b, the experimental XRD data of (ETP)2[{Mo6I8}Cl6] is not consistent with simulated data actually. We can observe the additional diffraction peak ~10-15°, therefore, it is necessary to re-test experimental XRD data. In addition, in order to facilitate a clearer observation, the diffraction angle range of the XRD data can be reduced.
4. In Figure 2d, temperature ranges is not given for (ETP)2[{Mo6I8}I6].
5. Figure 2e shows the temperature dependence of integrated PL intensity and lifetime in the temperatures range from ~80 to ~300 K, which is not consistent with the temperature range for temperature-dependent PL spectra in Figure 2d. It is suggested to unify the temperature range in Figure 1d and 1e.
6. Figure 4a is photographs, Figure 4b is PL spectra.
7. To demonstrated the dispersion effect, a series of hybrid glass systems have been used, does the PL properties of the glass matrix affect the performance of the final composite glass?
8. How to control the thickness of the hybrid metal halide glasses and the effect of glasses thickness on imaging resolution should be supplemented.
9. To disperse Mo6 clusters in zero-dimensional hybrid metal halide glasses to resolve the aggregation-induced quenching (ACQ) and 1O2-mediated quenching, this is new way to improve the luminescent properties of the {Mo6I8}4+ core. However, the advantages of this way is not reflected in this paper, and it should be reflected in the introduction.
10. The authors fabricated the NIR flashlight based on their new materials, and some recent advances on the new kinds of NIR emitters can be mentioned as the research background, as reported in Angew. Chem. Int. Ed., 2022, 61, e202208881.; Nature Communications, 2019, 10, 5267.

Version 1:

Reviewer comments:

Reviewer #1

(Remarks to the Author)
In my view, the authors have answered all the questions and revised related points, and thus this revised work can be published as it is.

Reviewer #2

(Remarks to the Author)

I have carefully reviewed the revised manuscript and I believe the authors have put good effort into highlighting the main feature of the study, which is control over oxygen quenching of the phosphorescence of the Mo6 clusters which greatly decrease their efficiency. They presented the challenges and rational to reach low oxygen quenching. They also added suitable references related to Mo6 clusters. I suggest acceptance of the manuscript in its current state.

Reviewer #3

(Remarks to the Author)
This manuscript has been properly revised and it is ready for the publication now.

**Open Access** This Peer Review File is licensed under a Creative Commons Attribution 4.0 International License, which permits use, sharing, adaptation, distribution and reproduction in any medium or format, as long as you give appropriate credit to the original author(s) and the source, provide a link to the Creative Commons license, and indicate if changes were made.
In cases where reviewers are anonymous, credit should be given to 'Anonymous Referee' and the source.
The images or other third party material in this Peer Review File are included in the article's Creative Commons license, unless indicated otherwise in a credit line to the material. If material is not included in the article's Creative Commons license and your intended use is not permitted by statutory regulation or exceeds the permitted use, you will need to obtain permission directly from the copyright holder.
To view a copy of this license, visit https://creativecommons.org/licenses/by/4.0/

# Response to the Reviewers

## Reviewer #1

This manuscript reports a zero-dimensional hybrid metal halide glass system incorporating $Mo_6$ clusters, and systematically investigates its near-infrared photoluminescence properties. Via materials synthesis, structural characterization, and spectroscopic investigations, the authors demonstrate broadband near-infrared emission and discuss its promising applications in optoelectronic devices and imaging. This work is relevant to the development of high-performance near-infrared luminescent materials and will be of broad interest to readers of Nature Communications. Overall, the manuscript presents relatively systematic experimental work, and multiple characterization results support the main conclusions. However, several aspects related to data presentation, mechanistic interpretation, and experimental details require further clarification and improvement to enhance the rigor and completeness of the study. The following comments are provided for the authors' revision.

1. In the structural analysis section, the authors mainly discuss the arrangement of the crystal structure, while the potential influence of variations in Mo-X bond lengths on the electronic structure and luminescent properties is not sufficiently explored. A more quantitative analysis of structural parameters is recommended to strengthen the discussion of the structure–property relationship.

**Response:** We thank the reviewer for this important suggestion. We have revised the structural analysis to provide a more quantitative correlation between Mo-X bonding, electronic structure, and luminescent behavior. Specifically, we have added a comparative discussion of the Mo-X bond lengths, Mo-Mo distances, and the corresponding photophysical parameters, including emission maxima, lifetimes, and quantum efficiencies. The revised discussion emphasizes that changing the apical ligand from Cl to Br and I simultaneously modifies the halogen p-orbital energy, orbital diffuseness, and Mo-X bond strength. The lower-energy and less diffuse Cl 3p orbitals, together with shorter Mo-Cl bonds, stabilize the occupied cluster-centered frontier orbitals and help maintain a wider optical gap. By contrast, Br and I ligands possess higher-energy and more diffuse p orbitals; the longer Mo-Br and Mo-I bonds weaken the Mo 4d-halogen p orbital interaction and raise the highest occupied cluster orbital, leading to a narrower gap and the observed red-shifted emission. This structure-property analysis is now integrated with the PDOS results in Fig. 3c and the crystallographic data summarized in Table S3. We believe that this revision provides a more complete structural basis for the halide-dependent emission shift and efficiency variation.

2. The molybdenum cluster samples with different halide ligands exhibit noticeably different absorption coefficients, yet the manuscript provides limited discussion regarding the underlying reasons. Further analysis of the potential influence of halogen atomic orbital characteristics on the absorption cross-section is recommended.

**Response:** We agree with the reviewer that the absorption-coefficient difference deserves a more explicit explanation. We have expanded the discussion by connecting the molar absorption coefficients with the halogen atomic orbital characteristics and Mo-X orbital hybridization. At 350 nm, the molar absorption coefficients of the Cl-, Br-, and I-terminated clusters are 5606,

7207, and 24024 $cm^{-1}$ $M^{-1}$, respectively, indicating a clear increase from Cl to I. In the revised manuscript, we explain that this trend arises from the progressively higher energy and greater spatial diffuseness of the halogen p orbitals from Cl 3p to Br 4p and I 5p. These orbital features increase the halogen contribution to the cluster frontier orbitals and enhance the transition dipole associated with charge-transfer and metal-metal/metal-ligand mixed transitions. This explanation is supported by the PDOS analysis and is discussed together with the ligand-dependent absorption behavior.

3. The temperature-dependent PL measurements only extend to 290 K. Considering that the material may operate under higher temperatures in practical applications, it is suggested to extend the measurement range or provide additional discussion regarding the thermal stability of the material at elevated temperatures.

**Response:** We thank the reviewer for this helpful comment. We clarify in the revised manuscript that the temperature-dependent PL measurements of the isolated $(ETP)_2[\{Mo_6I_8\}X_6]$ cluster powders were primarily designed to analyze the SOC/ZFS-mediated excited-state population dynamics from 100 to 290 K, rather than to evaluate device-level high-temperature operation. To address the reviewer's concern regarding practical thermal stability, we have added a more explicit discussion of the temperature-dependent PL behavior of the optimized $(ETP)_2CdCl_4:Mo_6$ glass over a broader temperature range (80–380 K; Fig. S23). The glass maintains nearly unchanged emission intensity from 80 to 290 K, whereas a more pronounced intensity decrease is observed at higher temperatures. This behavior is attributed to thermally activated molecular vibrations of the organic/inorganic hybrid matrix, which enhance nonradiative relaxation above room temperature. The revised text now distinguishes the mechanistic low-temperature measurements of the cluster powders from the operational thermal-stability assessment of the glass sample.

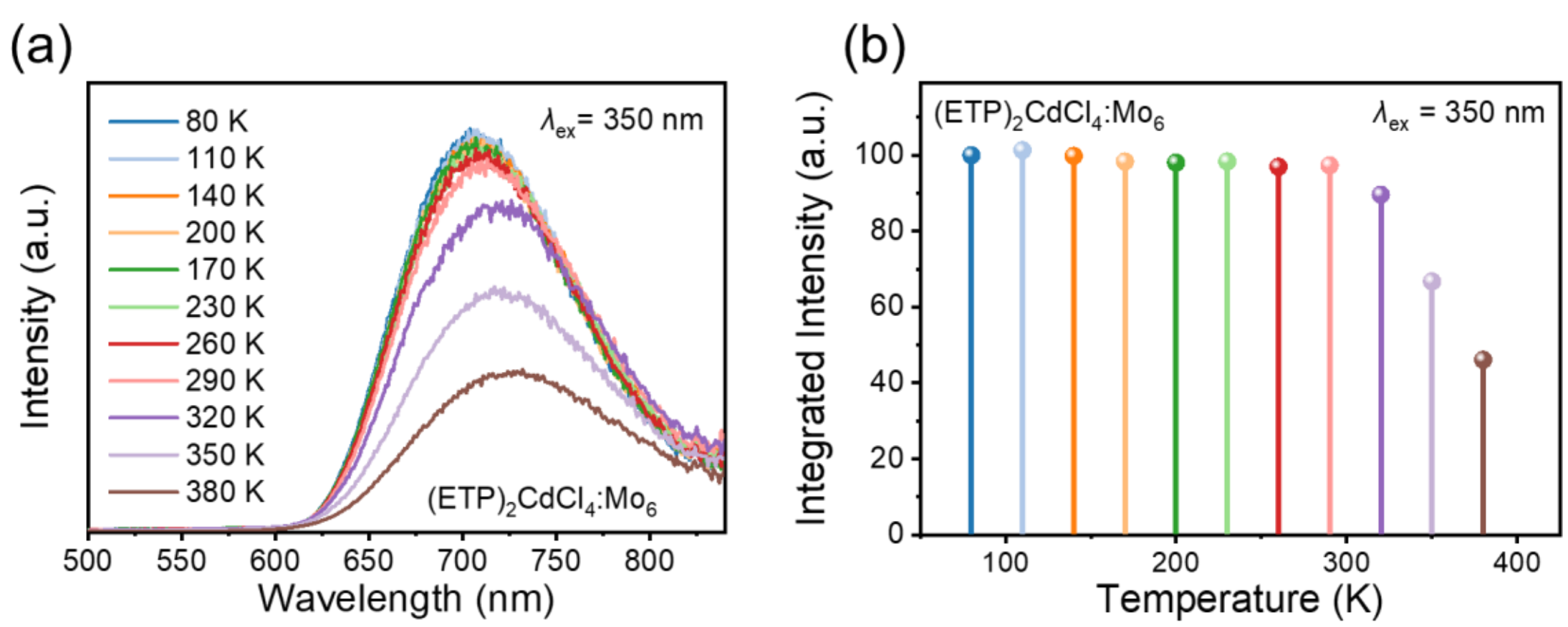


Figure S23. (a) Temperature-dependent PL spectra of $(ETP)_2CdCl_4:Mo_6$ glass and (b) corresponding integrated emission intensity.

4. Although relatively high IQE and EQE values are reported, the manuscript lacks repeated experiments or error analysis. Providing averaged values along with standard deviations from multiple measurements would improve the reliability of the reported data.

**Response:** We fully agree with the reviewer. To improve the statistical reliability of the reported efficiencies, we performed repeated measurements on independently prepared glass samples. Based on 18 repeated measurements (Table S8), the average IQE, absorption efficiency (AE), and EQE values are 96.85 ± 1.00%, 93.11 ± 0.43%, and 90.18 ± 1.07%, respectively (Table S9). These small standard deviations confirm the good reproducibility of the preparation and measurement procedures. The averaged values have now been added to the revised manuscript and Supplementary Information.

Table S9. Summary of repeated measurements of IQE, AE and EQE of $(ETP)_2CdCl_4$:$Mo_6$ glass

| Parameter | Mean ± s.d. | Minimum | Maximum | n |
|---|---|---|---|---|
| **IQE (%)** | 96.85 ± 1.00 | 95.14 | 98.86 | 18 |
| **AE (%)** | 93.11 ± 0.43 | 92.43 | 93.66 | 18 |
| **EQE (%)** | 90.18 ± 1.07 | 88.29 | 92.39 | 18 |

5. The resolution test was conducted using a USAF 1951 target; however, the measurement distance and optical system configuration are not specified. These key experimental parameters should be provided to improve the completeness of the experimental description.

**Response:** We thank the reviewer for pointing out this omission. We have revised the Methods section to include the experimental configuration used for the USAF 1951 resolution test. Specifically, the NIR camera was positioned approximately 10 cm in front of the USAF 1951 target, and the NIR flashlight was placed approximately 10 cm above the target to provide illumination. The experiment was conducted in a dark environment to minimize ambient-light interference.

6. It would be useful to provide spectral data for the glass samples after long-term storage in air in order to evaluate the environmental stability of the material. For potential device applications, additional photostability tests under continuous illumination are recommended to verify the practical applicability of the materials.

**Response:** We agree that long-term environmental stability and photostability are essential for evaluating practical applicability. In response, we have added spectral stability data for the optimized glass sample after storage in air and under continuous illumination. These results show that the NIR emission profile and intensity remain highly stable after long-term exposure to ambient conditions and under continuous UV excitation. The new data are now included in the Supplementary Information as Figure S26, and the corresponding discussion has been added to the Results section. This additional evidence further supports the robustness of the hybrid glass matrix in suppressing oxygen-related quenching and maintaining stable NIR output under application-relevant conditions.

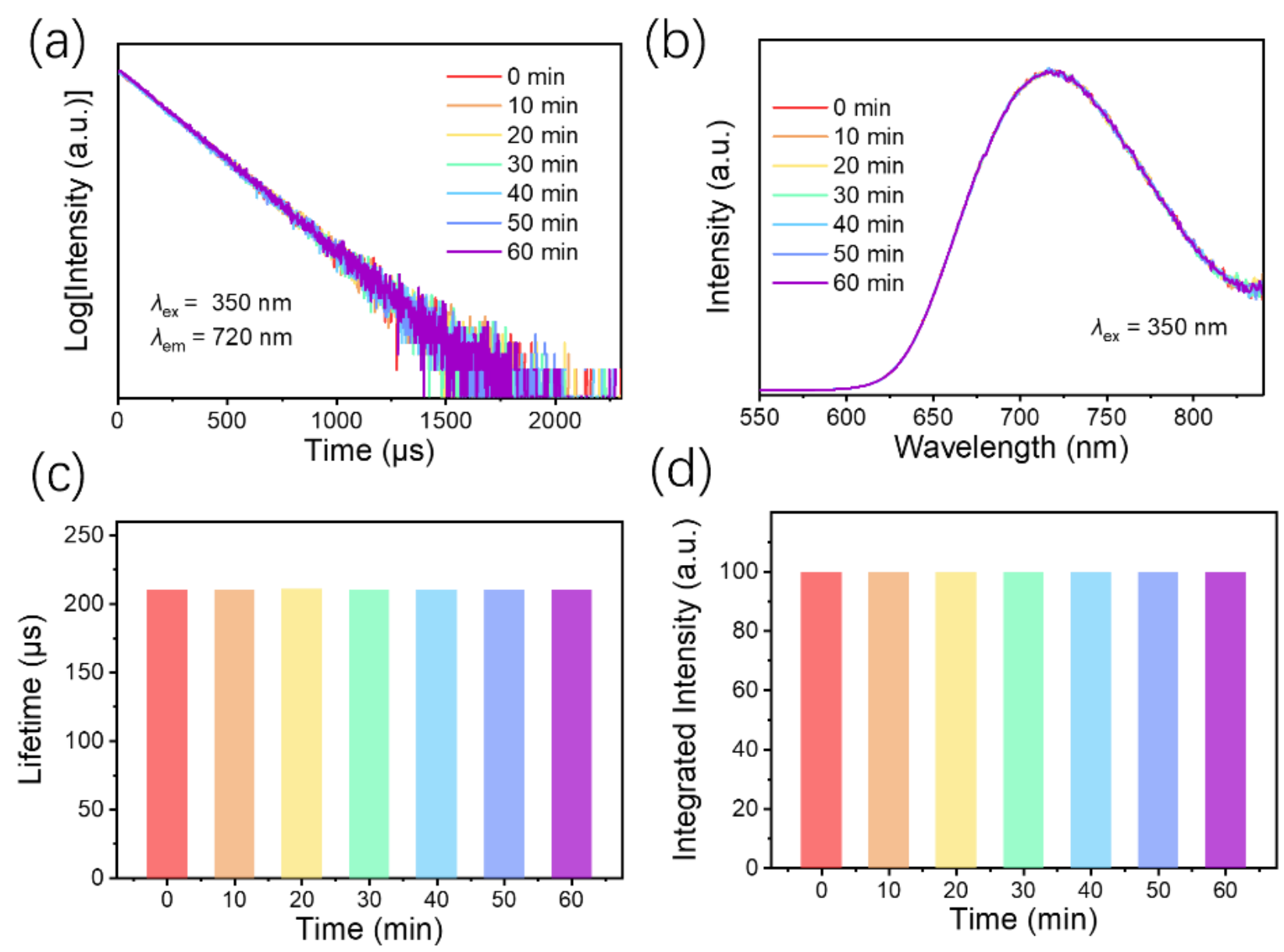


Figure S26 Emission stability of $(ETP)_2CdCl_4:Mo_6$ under continuous 350 nm UV irradiation. (a) Time-dependent PL decay curves. (b) PL spectra recorded at different irradiation times. (c) Evolution of the emission lifetime. (d) Evolution of the integrated PL intensity.

7. Molecular hybrid glasses and the wide photoemission properties are hot topics in materials and chemistry. To arouse a broad interest from readership in this field, some recent related works on recent fabrications of hybrid metal halide glass (Matter, 2024, 7, 1950; Acc. Chem. Res., 2025, 58, 3010; Angew. Chem. Int. Ed. 2026, e8889513) and high-performance luminescent applications (Nat. Commun. 2023, 14, 1654; Angew. Chem. Int. Ed. 2025, 64, e202420139; Sci. China Mater. 2024, 67, 3531) could be included as references.

**Response:** We thank the reviewer for the valuable literature suggestions. We have revised the Introduction to better position our work within the rapidly growing field of molecular hybrid glasses and hybrid metal halide glasses. The suggested references on recent fabrication strategies and high-performance luminescent/radiation-detection applications have been added and discussed. We now explicitly emphasize that molecular hybrid glasses combine the structural designability of molecular/ionic systems with the processability, transparency, and amorphous stability of conventional glasses. This broader context helps clarify why hybrid halide glasses provide a promising platform for converting molecular Mo6 cluster emitters into air-stable, highly efficient, processable NIR-emitting solids.

8. A comparison with other types of near-infrared luminescent materials (such as rare-earth ions, transition metal ions, and quantum dots, please checking Angew. Chem. Int. Ed. 2025, 64, e202505322) is recommended to more comprehensively demonstrate the advantages and characteristics of the present work.

**Response:** We agree with the reviewer. we have cited the recommended recent work (Angew. Chem. Int. Ed. 2025, 64, e202505322) and related literature. The revised discussion now clarifies the respective advantages and limitations of these material families and defines the distinct

characteristics of the present $Mo_6$-cluster-doped molecular hybrid metal halide glass, including strong charge-transfer absorption, high NIR emission efficiency in air, low-temperature melt processing and bulk glass formability.

## Reviewer #2

The manuscript by Feng et al. reports on the preparation and study of the photoluminescence of cluster compounds with general formula $(ETP)_2[Mo_6I_8X_6]$ (X=Cl, Br, and I). The compounds were further embedded in glass matrices leading to increase in photoluminescence quantum efficiency in air atmosphere.

Overall, the study is well conducted and the manuscript is well written, but there is a clear lack of novelty, as it is already known for almost 15 years that $Mo_6$ clusters especially those with iodine inner ligands can exhibit very high quantum yields of photoluminescence, close to unity, even in solution (10.1039/c3dt32863e; 10.1021/ic502144z; 10.1021/acs.inorgchem.9b02569). Most of this study is focused on features of these cluster that were previously demonstrated by other authors (temperature dependence of the photoluminescence, oxygen quenching, DFT calculations of the excited states). The main interest of the article is the ability to limit quenching by oxygen in the solid-state which can dramatically reduce the quantum efficiency in air atmosphere. However, it is not a newly discovered feature of $Mo_6$ cluster compounds (10.1039/C6DT02863B) and no suitable explanation can be found in the manuscript as to why these specific compounds show limited oxygen quenching.

For these reasons I do not recommend publication of the manuscript in Nature Communications and I suggest submission to a more specialized journal.

**Response:** We sincerely thank the reviewer for the careful and expert assessment. We agree with the reviewer that several fundamental photophysical features of $Mo_6$ clusters, including high PLQY in deoxygenated solution, temperature-dependent emission dynamics, oxygen sensitivity, and excited-state calculations, have been established in previous studies. We have therefore revised the manuscript to avoid presenting these known features as the main novelty. The central message of the revised manuscript is now substantially refocused: the advance is not simply that $Mo_6$ clusters can be highly emissive, but that molecular hybrid metal halide glass can serve as a rigid and adaptive ionic host that converts oxygen-sensitive molecular $Mo_6$ triplet emitters into air-stable, molecularly dispersed, processable bulk NIR-emitting solids.

To address the reviewer's concern regarding why the present glass system shows limited oxygen quenching, we have added new air/$N_2$ lifetime measurements and a new mechanistic figure in the revised manuscript (Figure 5). To directly compare the oxygen-quenching resistance of $Mo_6$ emitters in different physical environments, we defined a lifetime retention ratio, $Q_\tau = \tau_{\mathrm{air}}/\tau_{\mathrm{deoxy}} \times 100\%$, based on the lifetime form of the Stern-Volmer quenching relationship (Physikalische Zeitschrift 1919, 20, 183). Here, $\tau_{\mathrm{deoxy}}$ denotes the lifetime measured under the corresponding low-oxygen condition. A larger $Q_\tau$ indicates that the emissive triplet state is less affected by oxygen under ambient air. Specifically, the DMF solution was deoxygenated by $N_2$ bubbling, whereas the solid powder, glass powder and bulk glass samples were measured after vacuum evacuation followed by dry-$N_2$ backfilling. Four representative sample states were directly compared: molecularly dispersed $(ETP)_2[\{Mo_6I_8\}Cl_6]$ in DMF, pure $(ETP)_2[\{Mo_6I_8\}Cl_6]$

molecular powder, $(ETP)_2CdCl_4$ glass powder, and bulk $(ETP)_2CdCl_4$ glass. The resulting $Q_\tau$ values are 3.35%, 91.40%, 95.36%, and 98.96%, respectively.

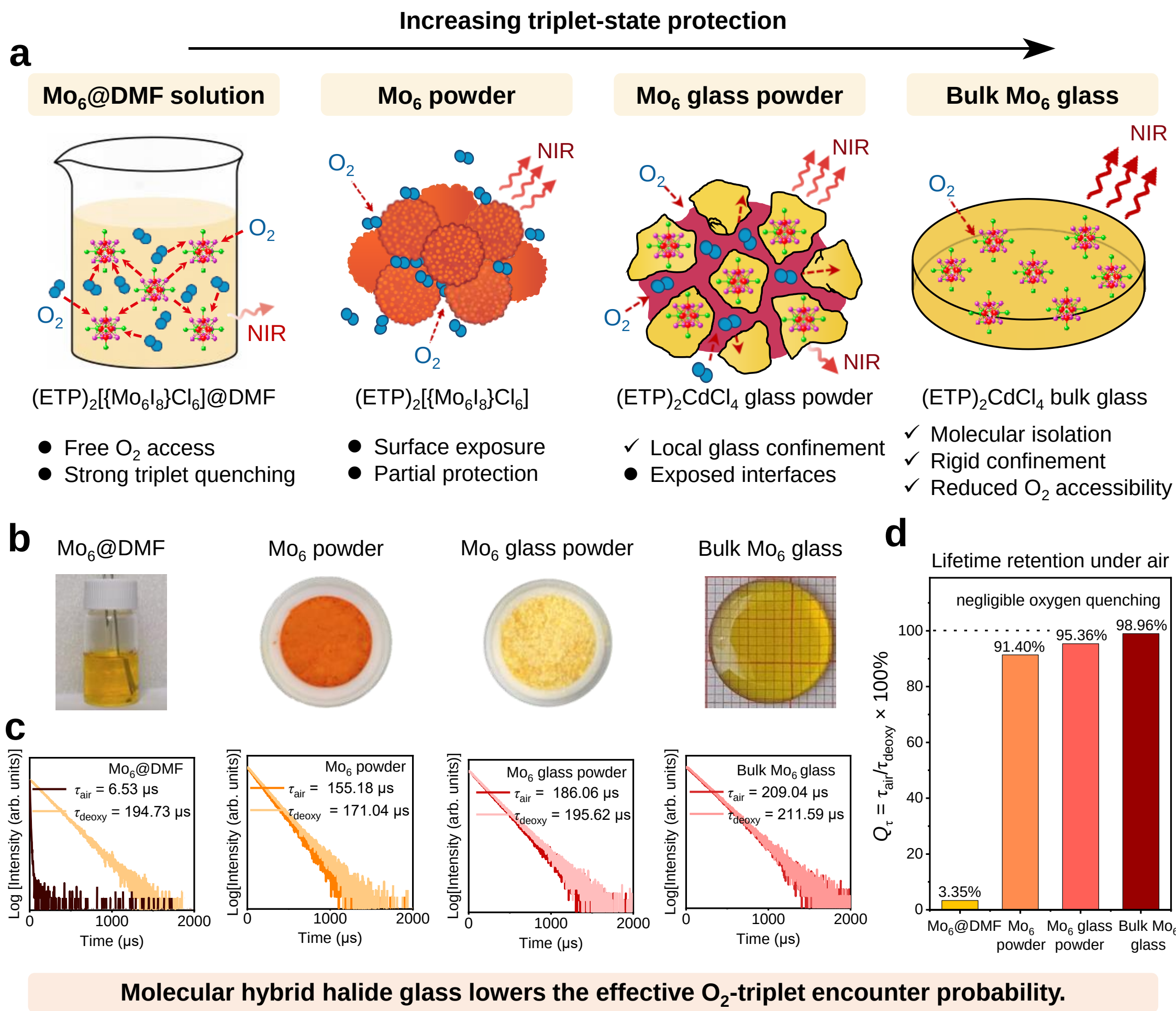


Figure 5. Environment-dependent oxygen-quenching resistance of $Mo_6$ cluster emitters. (a) Schematic illustration of oxygen accessibility and triplet-state protection in DMF solution, molecular powder, glass powder and bulk molecular hybrid halide glass. (b) Representative photographs of the corresponding samples. (c) PL decay curves measured under air and $N_2$ atmospheres. (d) Lifetime retention ratios showing a pronounced increase from $Mo_6$@DMF to bulk $Mo_6$ glass.

This systematic trend provides direct experimental evidence that oxygen-quenching suppression is strongly environment-dependent. In DMF solution, the $Mo_6$ clusters are molecularly dispersed, but their long-lived triplet excited states are freely accessible to dissolved oxygen, resulting in severe dynamic quenching and a very low $Q_\tau$ of 3.35%. In the pure molecular powder, oxygen access is partially restricted by the solid state, increasing $Q_\tau$ to 91.40%, but surface exposure, defects, and cluster–cluster contacts can still contribute to triplet-state deactivation. In the glass powder, the $Mo_6$ centers remain locally confined by the vitrified matrix, further increasing $Q_\tau$ to 95.36%; however, grinding introduces exposed fractured interfaces and increased surface area. The intact bulk molecular hybrid metal halide glass exhibits the highest $Q_\tau$ of 98.96%,

demonstrating that the glass matrix provides the most effective protection against oxygen-induced triplet quenching under ambient air.

These new data directly support the revised mechanistic interpretation. The molecular hybrid metal halide glass does not merely act as a passive physical barrier. During glass formation, the halide-rich ionic melt promotes molecular-level cluster dispersion and matrix-dictated halide-environment redistribution, as evidenced by the convergence of emission spectra, lifetimes, and quantum efficiencies for different initial cluster ligands within the same glass matrix. After vitrification, the rigid amorphous matrix freezes the dispersed emissive centers, suppresses cluster–cluster interactions and vibrationally assisted nonradiative relaxation, and markedly reduces the effective encounter probability between molecular oxygen and the long-lived $Mo_6$ triplet excited state. Therefore, the glass suppresses oxygen quenching through coupled chemical and structural effects: molecular isolation, rigid confinement, reduced oxygen accessibility, and matrix-dictated emissive-center formation.

We have accordingly rewritten the Abstract, Introduction, glass-related Results section, and Conclusion, and have moderated the wording throughout the manuscript. Specifically, we no longer claim that oxygen-quenching suppression itself is a newly discovered phenomenon for $Mo_6$ clusters. Instead, we clarify that the novelty lies in establishing a molecular hybrid metal halide glass strategy that integrates ligand/matrix co-regulation, molecular dispersion, triplet-state protection, and bulk solid-state processability. We believe that the newly added air/low-oxygen lifetime data and the mechanistic figure directly address the reviewer’s concern about the origin of limited oxygen quenching and more accurately define the conceptual advance of the work. Because molecular hybrid metal halide glasses, triplet-state protection, and high-efficiency NIR emitters are active topics across materials chemistry, photophysics, and optoelectronics, we respectfully believe that the revised manuscript is of broad interest to the readership of Nature Communications rather than being limited to a specialized $Mo_6$-cluster audience.

## Reviewer #3

In this study, the authors designed and synthesized a series of isostructural molybdenum clusters $(ETP)_2[\{Mo_6I_8\}X_6]$ (X = Cl, Br, I) via ligand exchange method in high-temperature ionic liquid, and revealing systematic correlations between outer ligands and the electronic and luminescent properties of the $\{Mo_6I_8\}^{4+}$ core. Further, due to the suppresses cluster vibrations and nonradiative transitions, embedding $[\{Mo_6I_8\}Cl_6]^{2-}$ cluster into $(ETP)_2CdCl_4$ glass matrix markedly enhances cluster dispersion and photostability, achieving 96.95% 43 IQE and 91.43% EQE. This study delivers a timely and valuable contribution to the field of next-generation high-efficiency NIR light sources, particularly in the context of structural modulation strategies aimed at enhancing performance of octahedral molybdenum cluster luminescent materials. The reviewer thinks this work is interesting and is worthy of publication. However, the following issues should be well addressed:

1. One of the key points in this manuscript is about the preparation of the hybrid metal halide glasses, and some of the recent advances in this topic should be described, as reported in

Advanced Functional Materials, 2026, 36(23) e21093.; Matter, 2025, 102214.; Chemical Science, 2023, 14, 12238.

**Response:** We thank the reviewer for this important suggestion. We have expanded the Introduction to include recent advances in hybrid metal halide glasses, including solution-evaporation-induced supramolecular glasses, melt-quenched zero-dimensional hybrid metal halide glasses, and glass-ceramic systems formed via in situ recrystallization. The suggested references have been added to the revised manuscript. This additional background helps establish the broader relevance of hybrid metal halide glasses as structurally tunable and processable optical materials, and it better introduces the rationale for developing high-efficiency NIR-emissive hybrid glasses based on $Mo_6$ clusters.

2. Figure S1 demonstrates the absorption spectra, not diffuse reflectance spectra.

**Response:** We thank the reviewer for pointing this out. We have revised Fig. S1 and the corresponding caption. The spectra are now described as absorption spectra derived from diffuse-reflectance measurements, rather than direct diffuse-reflectance spectra. The related text in the manuscript and Supplementary Information has also been corrected accordingly.

3. In Figure 1b, the experimental XRD data of $(ETP)_2[\{Mo_6I_8\}Cl_6]$ is not consistent with simulated data actually. We can observe the additional diffraction peak ~10-15°, therefore, it is necessary to re-test experimental XRD data. In addition, in order to facilitate a clearer observation, the diffraction angle range of the XRD data can be reduced.

**Response:** We agree with the reviewer. We have re-measured the powder XRD pattern of $(ETP)_2[\{Mo_6I_8\}Cl_6]$ and updated Fig. 1b. The revised XRD pattern now shows good agreement with the simulated pattern derived from the single-crystal structure, and no obvious additional peak is observed in the 10-15° region. We have also reduced the displayed diffraction range to 5-40° to make the comparison clearer. The relevant text has been revised accordingly.

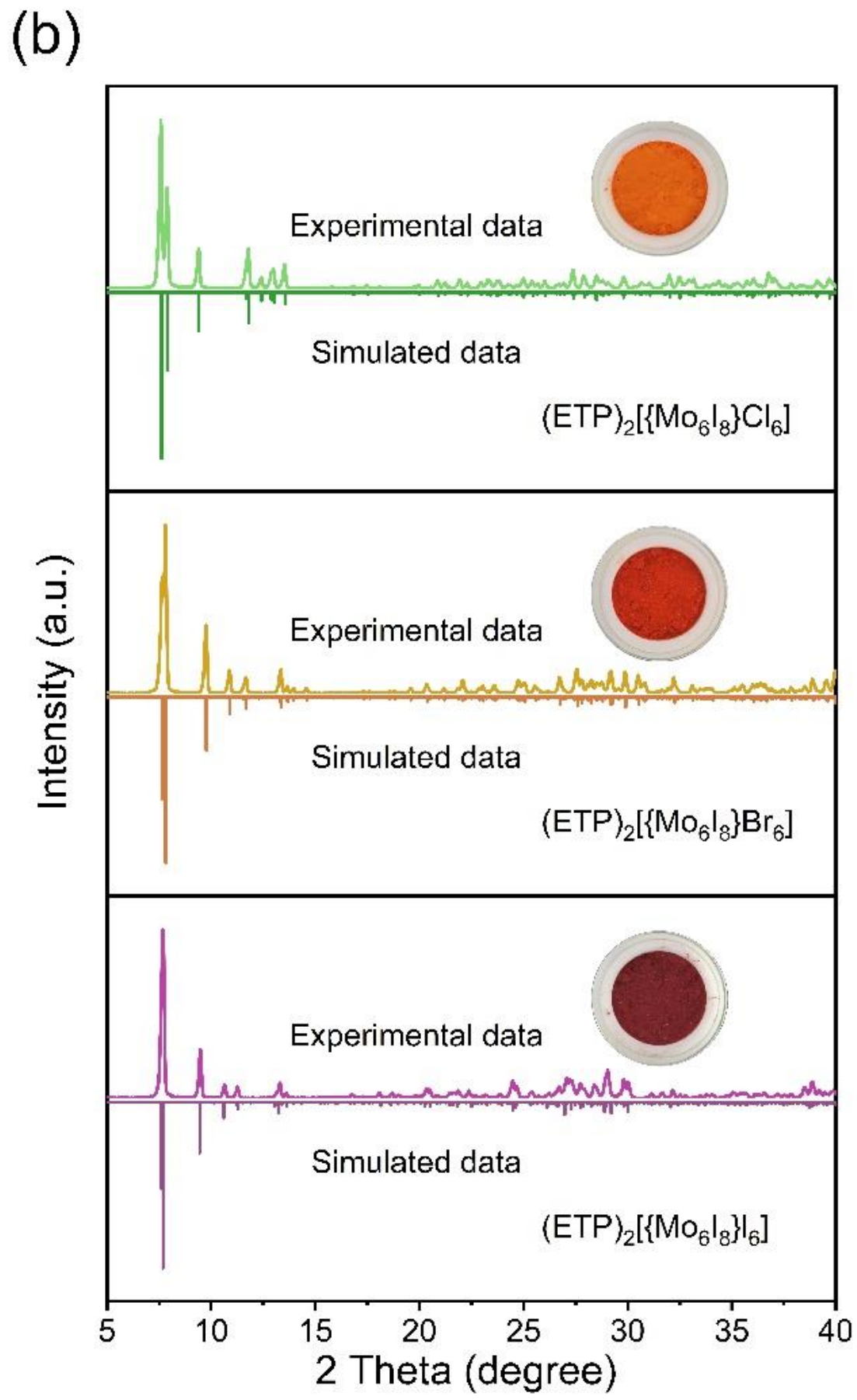


**Figure 1.** (b) Experimental and simulated powder XRD patterns of $(ETP)_2[\{Mo_6I_8\}X_6]$ powders.

4. In Figure 2d, temperature ranges is not given for $(ETP)_2[\{Mo_6I_8\}I_6]$.

**Response:** We thank the reviewer for pointing this out. In the original dataset, all three samples, $(ETP)_2[\{Mo_6I_8\}X_6]$ (X = Cl, Br, I), were measured over the same temperature range of 100-290 K. The confusion arose from the layout of the original figure, in which a single temperature legend was used for multiple panels. In the revised Figure 2d, we have added clear temperature legends for each sample and revised the caption to explicitly state that the temperature-dependent PL spectra of all three samples were collected from 100 to 290 K.

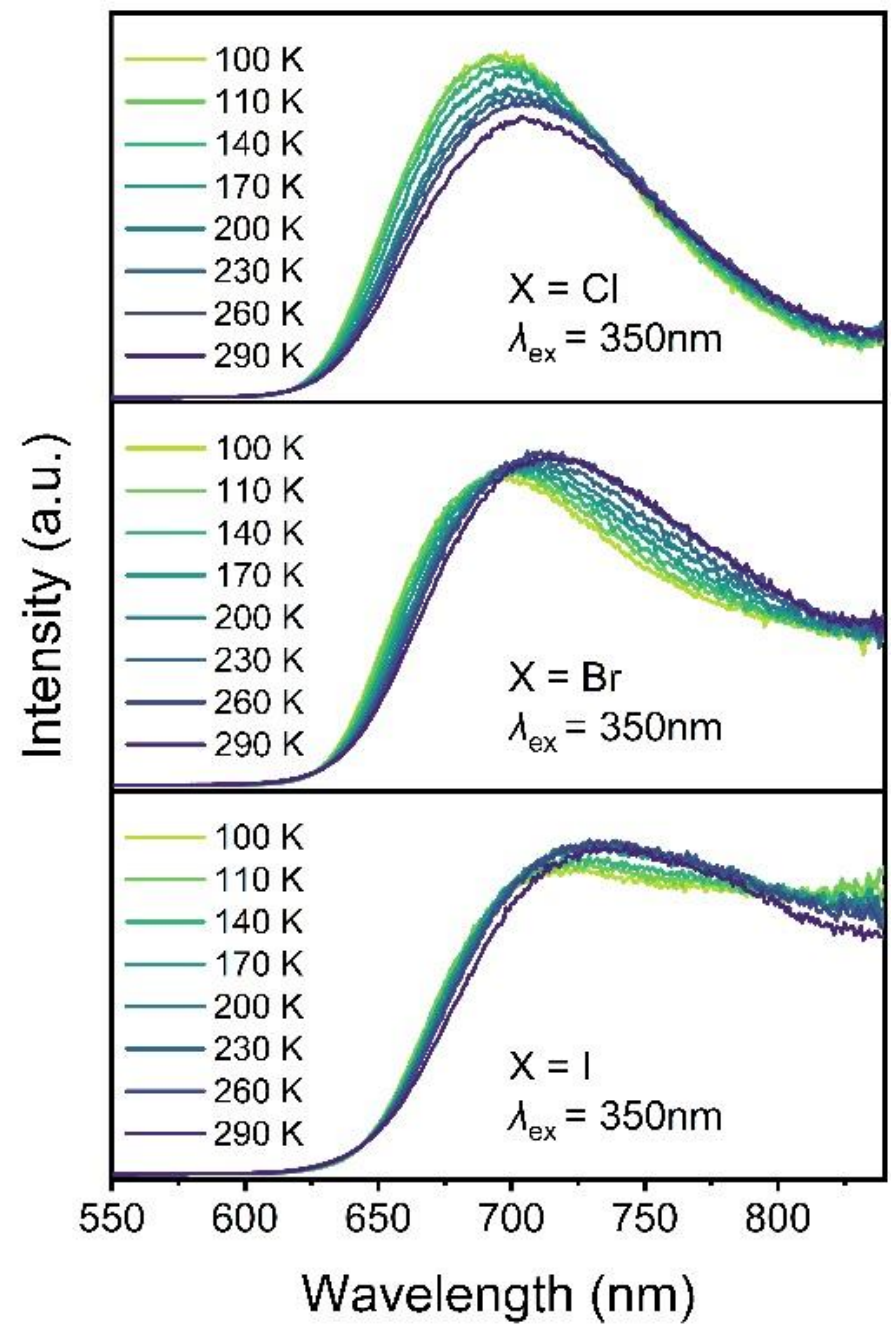


**Figure 2.** (d) Temperature-dependent PL spectra of $(ETP)_2[\{Mo_6I_8\}X_6]$.

5. Figure 2e shows the temperature dependence of integrated PL intensity and lifetime in the temperatures range from ~80 to ~300 K, which is not consistent with the temperature range for temperature-dependent PL spectra in Figure 2d. It is suggested to unify the temperature range in Figure 1d and 1e.

**Response:** We agree. The apparent inconsistency resulted from the unclear labeling in the original figure. The actual temperature range used for the temperature-dependent PL spectra and the integrated intensity/lifetime plots is the same for the three cluster powders. We have revised Fig. 2d and Fig. 2e to use consistent temperature labeling and have corrected the figure caption accordingly.

6. Figure 4a is photographs, Figure 4b is PL spectra.

**Response:** We thank the reviewer for identifying this labeling error. We have corrected the Fig. 4 caption so that Fig. 4a is described as photographs and Fig. 4b as PL spectra. We have also checked the figure labels, captions, and cross-references throughout the manuscript and Supplementary Information to eliminate similar inconsistencies.

7. To demonstrated the dispersion effect, a series of hybrid glass systems have been used, does the PL properties of the glass matrix affect the performance of the final composite glass?

**Response:** This is an important point. We have revised the discussion to distinguish the intrinsic PL contribution of the glass matrix from the matrix effect on Mo6-cluster emission. The final NIR emission of the composite glasses is dominated by the embedded $[\{Mo_6I_8\}Cl_6]^{2-}$ cluster, as shown by the characteristic NIR emission profiles in Fig. 4f and Fig. S18. Under 450 nm excitation, the Sb-containing glass matrices show negligible matrix emission, and the observed emission is mainly from the Mo6 cluster. We also note that some Sb-based matrices can show additional emission under other excitation wavelengths; therefore, the glass matrix may influence

the spectral details under specific excitation conditions. However, the core NIR luminescence performance of the composite glass is governed primarily by the dispersed $Mo_6$ cluster, while the matrix mainly regulates excitation absorption, cluster dispersion, local rigidity, and nonradiative decay pathways. This clarification has been added to the revised manuscript.

8. How to control the thickness of the hybrid metal halide glasses and the effect of glasses thickness on imaging resolution should be supplemented.

**Response:** We thank the reviewer for this useful suggestion. We have added a description of the thickness-control method and the thickness-dependent imaging experiment. The $(ETP)_2CdCl_4{:}Mo_6$ glasses were prepared by melt quenching, and the sample thickness was controlled by adjusting the precursor loading under otherwise identical preparation conditions. Using precursor masses of 1.0, 1.5, 2.0, and 2.5 g, we obtained glass samples with thicknesses of 1.51, 2.62, 4.33, and 5.74 mm, respectively. As shown in Figure S27, we then evaluated the imaging resolution for these samples. Within this thickness range, no obvious degradation in imaging resolution was observed. This is because, in the present UV-excited NIR flashlight configuration, the glass functions as a luminescent illumination source rather than as a direct imaging scintillator layer. The final spatial resolution is therefore determined mainly by the optical imaging system, target geometry, and camera resolution, provided that the glass maintains sufficient transparency and uniform emission. We have added this discussion to clarify the difference between our NIR-light-source configuration and X-ray scintillator imaging, where increased scintillator thickness can directly degrade spatial resolution through lateral light spreading.

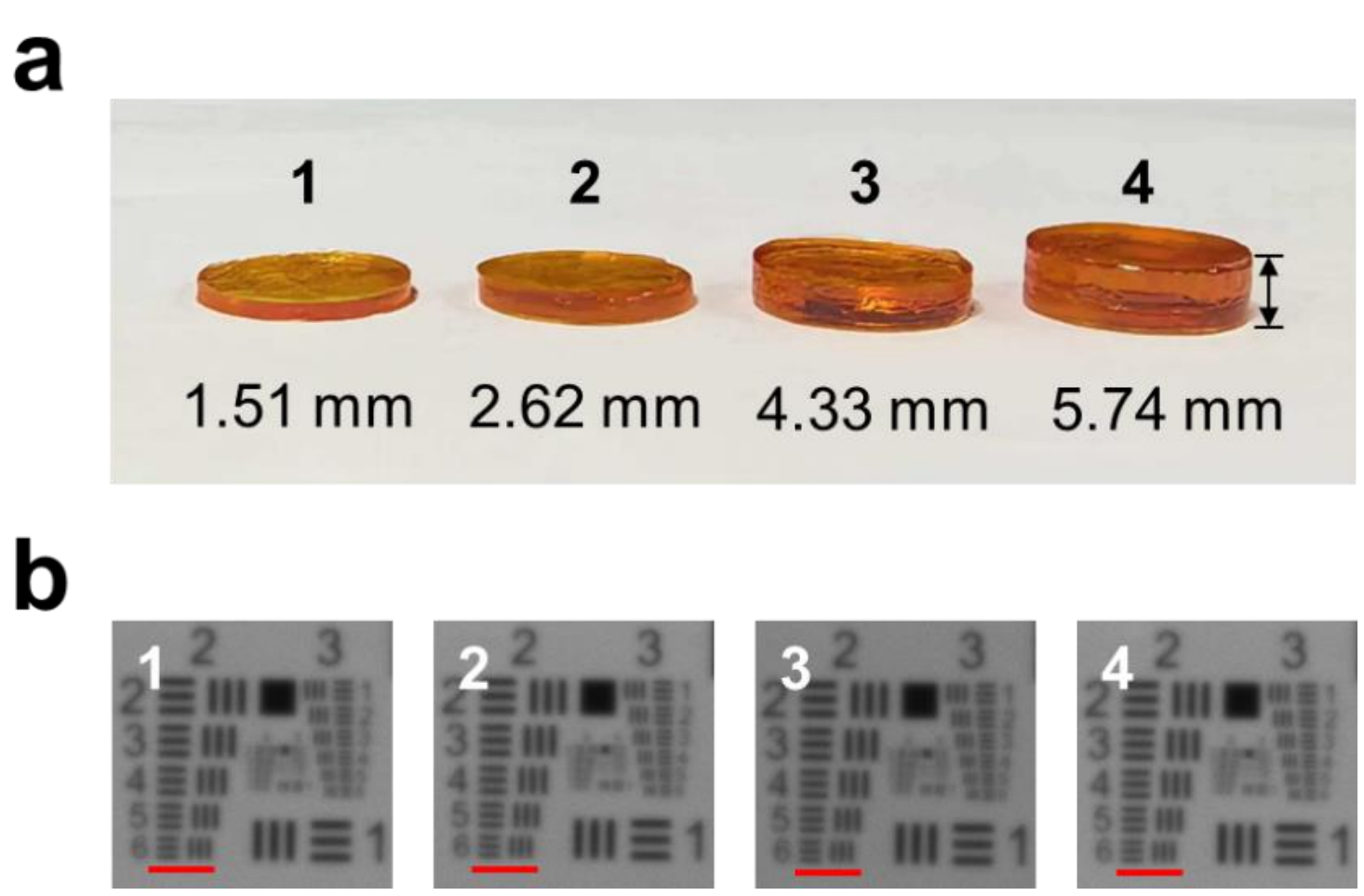


Figure S27. Thickness-dependent imaging performance of $(ETP)_2CdCl_4{:}Mo_6$ glasses. (a) Photographs of $(ETP)_2CdCl_4{:}Mo_6$ glass samples with thicknesses of 1.51, 2.62, 4.33 and 5.74 mm, controlled by adjusting the precursor loading during melt-quenching preparation. (b) NIR images of a USAF 1951 resolution target acquired using the corresponding glass samples as UV-excited NIR illumination sources. The red lines indicate the same reference region used for visual comparison of the imaging resolution.

9. To disperse $Mo_6$ clusters in zero-dimensional hybrid metal halide glasses to resolve the aggregation-induced quenching (ACQ) and $^1O^{2-}$mediated quenching, this is new way to improve

the luminescent properties of the $\{Mo_6I_8\}^{4+}$ core. However, the advantages of this way is not reflected in this paper, and it should be reflected in the introduction.

**Response:** We fully agree with the reviewer. This comment is closely related to the major concern raised by Reviewer #2, and it prompted us to substantially rewrite the Abstract and Introduction. In the revised manuscript, we now explicitly state that dispersing $Mo_6$ clusters in molecular hybrid halide glasses offers several advantages over solution, powder, and conventional polymer-film environments: (i) molecular-level isolation of the cluster emitters, which suppresses aggregation-induced quenching; (ii) rigid confinement within an amorphous ionic glass, which suppresses vibrationally assisted nonradiative relaxation; (iii) reduced oxygen accessibility to the long-lived $Mo_6$ triplet state, which mitigates oxygen-induced quenching in air; (iv) matrix-dictated halide-environment redistribution during melt processing, which allows the glass composition to regulate the final emissive center; and (v) bulk processability and device compatibility. We have introduced this design rationale in the Introduction and further illustrated it in a new mechanistic schematic Figure 5.

10. The authors fabricated the NIR flashlight based on their new materials, and some recent advances on the new kinds of NIR emitters can be mentioned as the research background, as reported in Angew. Chem. Int. Ed., 2022, 61, e202208881.; Nature Communications, 2019, 10, 5267.

**Response:** We thank the reviewer for the helpful suggestion. We have added the recommended references and expanded the Introduction to discuss recent advances in NIR emitters and NIR light-source materials. The revised background now better compares the present $Mo_6$-cluster-doped hybrid glass with other emerging NIR emitters and clarifies why high absorption efficiency, air-stable NIR emission, and low-temperature processability are important for practical NIR lighting and imaging applications.

# Response to the Reviewers

## Reviewer #1 (Remarks to the Author):

In my view, the authors have answered all the questions and revised related points, and thus this revised work can be published as it is.

Response: We sincerely thank the reviewer for the positive evaluation and for recommending publication of our revised manuscript.

## Reviewer #2 (Remarks to the Author):

I have carefully reviewed the revised manuscript and I believe the authors have put good effort into highlighting the main feature of the study, which is control over oxygen quenching of the phosphorescence of the $Mo_6$ clusters which greatly decrease their efficiency. They presented the challenges and rational to reach low oxygen quenching. They also added suitable references related to Mo6 clusters. I suggest acceptance of the manuscript in its current state.

Response: We sincerely thank the reviewer for the careful assessment and positive comments. We are grateful that the revisions and additional discussion were considered satisfactory.

## Reviewer #3 (Remarks to the Author):

This manuscript has been properly revised and it is ready for the publication now.

Response: We sincerely thank the reviewer for the positive evaluation and for supporting publication of our revised manuscript.
